# Supplementary material for: Repeat controlled human Plasmodium falciparum infections delay bloodstream patency and reduce symptoms
Source: Nat Commun. 2024 Jun 18;15:5194. doi: 10.1038/s41467-024-49041-2 (PMC11189388; doi:10.1038/s41467-024-49041-2)
Supplement: Supplementary file 1 — Supplementary Information [file 41467_2024_49041_MOESM1_ESM.pdf]

Supplementary Materials

Repeat controlled human *Plasmodium falciparum* infections delay bloodstream patency and reduce symptoms

Patricia Ferrer[1,2], Andrea A. Berry[3], Allison N. Bucsan[1,2], Surendra K. Prajapati[1,2], Karthik Krishnan[1,2], Michelle C. Barbeau[1,2], David M. Rickert[1,2], Sandra Mendoza Guerrero[1,2], Miho Usui[1,2], Yonas Abebe[4], Asha Patil[4], Sumana Chakravarty[4], Peter F. Billingsley[4], Faith Pa'ahana-Brown[3], Kathy Strauss[3], Biraj Shrestha[3], Effie Nomicos[5], Gregory A. Deye[5], B. Kim Lee Sim[4], Stephen L. Hoffman[4], and Kim C. Williamson[1]*, Kirsten E. Lyke[3]*

*corresponding authors contributed equally,

[1]Department of Microbiology and Immunology, Uniformed Services University of the Health Sciences, Bethesda, MD, USA. [2]Henry M. Jackson Foundation for the Advancement of Military Medicine, Inc., Rockville, MD, USA. [3]Center for Vaccine Development and Global Health, University of Maryland School of Medicine, Baltimore, MD, USA.

[4]Sanaria Inc., Rockville, MD, USA. [5]Parasitology and International Programs Branch, Division of Microbiology and Infectious Diseases, NIAID, NIH, Bethesda, MD, USA

Table of Contents

Figures 1-10....................................pgs 1 - 14

Inclusion and Exclusion Criteria.....pgs 15 - 16

Study Protocol.................................pgs 17 - 126

NUMBER OF CHALLENGES

CHMI 1

Symptom	002	004	009	017	010	016	027	019
Abdominal pain		3	1		2			
Joint pain		3	2		3			
Chills		3	1		2	1	1	
Dizziness		2			3			
Headache	1	5	3	3	4	1	3	1
Malaise	2	3	2		3	1	1	
Myalgia		3	2	1	2	1		
Nausea		2	1		2			
Vomiting								
Fever		38.9		39.4	39.3			
**Total # symptoms**	**2**	**9**	**7**	**3**	**9**	**4**	**3**	**1**
**Total # times**	**3**	**25**	**12**	**5**	**22**	**4**	**5**	**1**
**Day of symptom onset**	12	9	11	12	8	12	12	11
**Day of parasite appearance (detected by TBS)**	12	9	12	12	12	12	12	10
**Parasitemia level (PCR) (p/ul)**	2.3	0.8	41.3	6.2	28.4	3.2	2.4	2.5
**Parasitemia level (TBS) (p/ul)**	4.7	4.7	63.5	49.4	37.6	4.7	14.1	7.1
**Total # subjects with any symptom**	8/8 = 100%							

CHMI 2

Symptom	002	004	009	017	010	016	027	019
Abdominal pain							5	
Joint pain								
Chills			2			3	1	1
Dizziness		4						
Headache		6	1	1	3	2	3	4
Malaise	1					1		1
Myalgia		2	2		2			
Nausea		3				1		
Vomiting						1		
Fever	38.3							38.8
**Total # symptoms**	**2**	**4**	**3**	**1**	**2**	**5**	**3**	**4**
**Total # times**	**2**	**15**	**6**	**1**	**5**	**8**	**9**	**7**
**Day of symptom onset**	14	7	9	12	7	14	9	13
**Day of parasite appearance (detected by TBS)**	14	12	9	12	13	15	10	13
**Parasitemia level (PCR) (p/ul)**	3.4	3.1	0.4	4.2	5.1	34.4	0.3	7.5
**Parasitemia level (TBS) (p/ul)**	23.5	4.7	9.4	4.7	4.7	16.5	7.1	25.9
**Total # subjects with any symptom**	8/8 = 100%							

CHMI 3

Symptom	002	004	009	017	010	016	027	019
Abdominal pain								
Joint pain			2					
Chills						4	5	3
Dizziness								
Headache		2	4	2	3	2	6	2
Malaise						3	3	
Myalgia			1			2		2
Nausea								
Vomiting						1		
Fever						38	39	
**Total # symptoms**	**0**	**1**	**3**	**1**	**1**	**6**	**4**	**3**
**Total # times**	**0**	**2**	**7**	**2**	**3**	**13**	**14**	**7**
**Day of symptom onset**		12	12	12	13	15	13	13
**Day of parasite appearance (detected by TBS)**	12	12	12	12	13	15	15	15
**Parasitemia level (PCR) (p/ul)**	0.7	1.4	3.3	3.9	0.7	20.8	5.4	4.5
**Parasitemia level (TBS) (p/ul)**	4.7	7.1	21.2	7.1	4.7	32.9	23.5	21.2
**Total # subjects with any symptom**	7/8 = 87.5%							

CHMI 4

Symptom	002	004	009	017	010
Abdominal pain					1
Joint pain					1
Chills					5
Dizziness					2
Headache					1
Malaise				2	3
Myalgia					1
Nausea					
Vomiting					
Fever					
**Total # symptoms**	**0**	**0**	**0**	**1**	**7**
**Total # times**	**0**	**0**	**0**	**2**	**14**
**Day of symptom onset**				8	13
**Day of parasite appearance (detected by TBS)**	17	12	12	12	16
**Parasitemia level (PCR) (p/ul)**	7.0	1.6	1.6	2.1	1.5
**Parasitemia level (TBS) (p/ul)**	28.2	11.8	4.7	14.1	18.8
**Total # subjects with any symptom**	2/5 = 40%				

Supplementary Figure 1. Severity and number of symptoms decreased after repetitive CHMI. (A) The number of times a participant (CHMI1 n= 8, CHMI2 n= 8, CHMI3 n= 8, CHMI4 n=5) reported a symptom per CHMI as well as the total number and frequency of symptoms and the day of symptom onset is is shown. Days are counted from the most recent mosquito exposure and only symptoms reported after Day 6 until parasite clearance are shown. Symptom severity is indicated by color, Red = severe, orange = moderate, yellow = mild. An infectivity summary including the day of parasite detection by thick blood smear (TBS) and peak parasitemia quantified by quantitative polymerase chain reaction (PCR) and TBS is also shown.

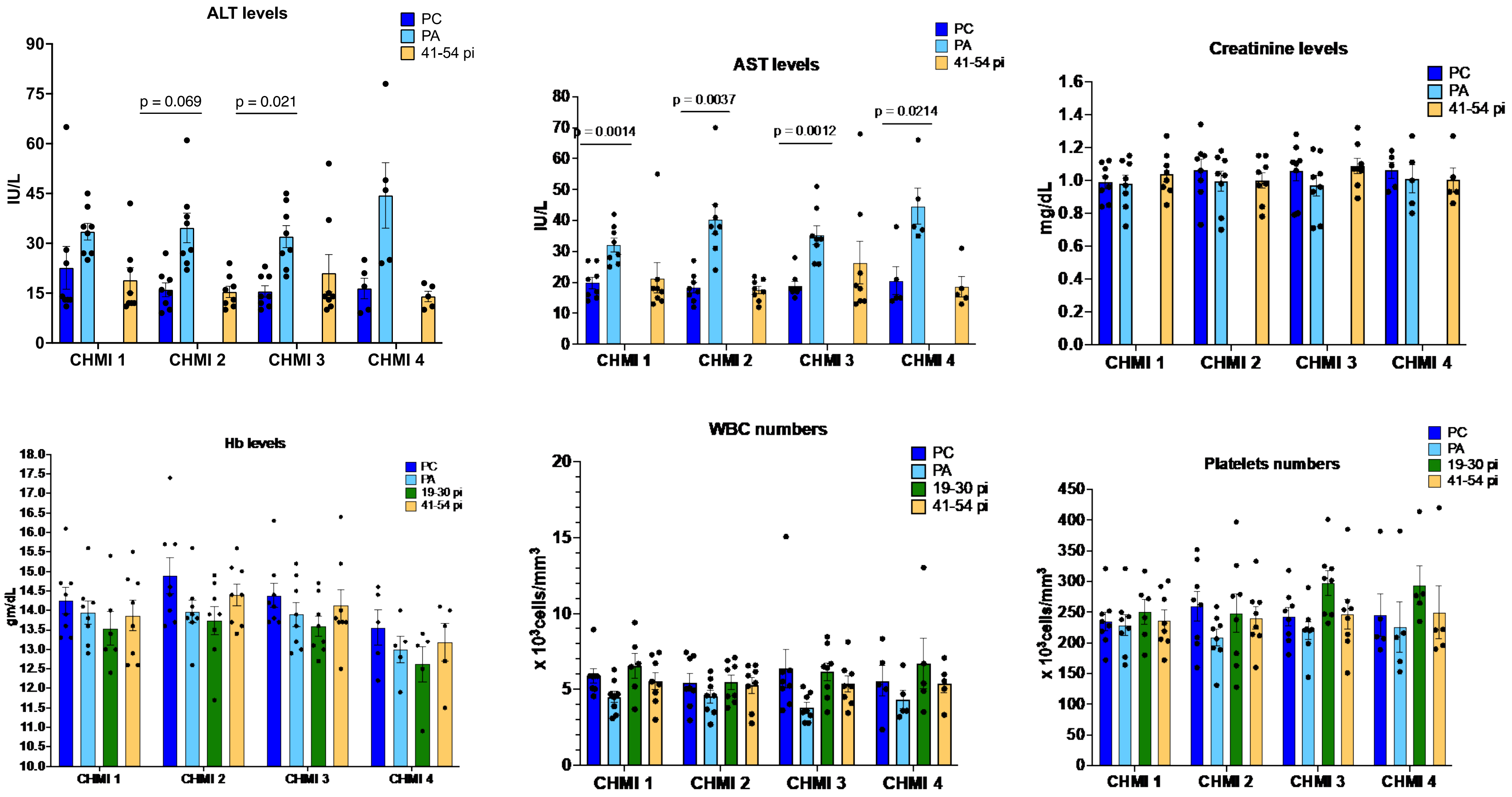

Supplementary Figure 2. ALT and AST levels increase at peak parasitemia. Plots show six clinical laboratory parameters quantified before infection = PC (dark blue), on patency day = PA (light blue), 19-31 days post infection = 19-30 pi (green) and 41-55 post infection = 41-54 pi (yellow), for all participants per CHMI (CHMI1 n= 8, CHMI2 n= 8, CHMI3 n= 8, CHMI4 n=5, ALT = alanine aminotransferase, AST = aspartate aminotransferase). Data are presented as mean and SEM. Statistical differences within each CHM between PC and each other day were tested using Dunnett's multiple comparisons test, with individual variances computed for each comparison. All P values are two-sided and source data are provided as a Source Data file.

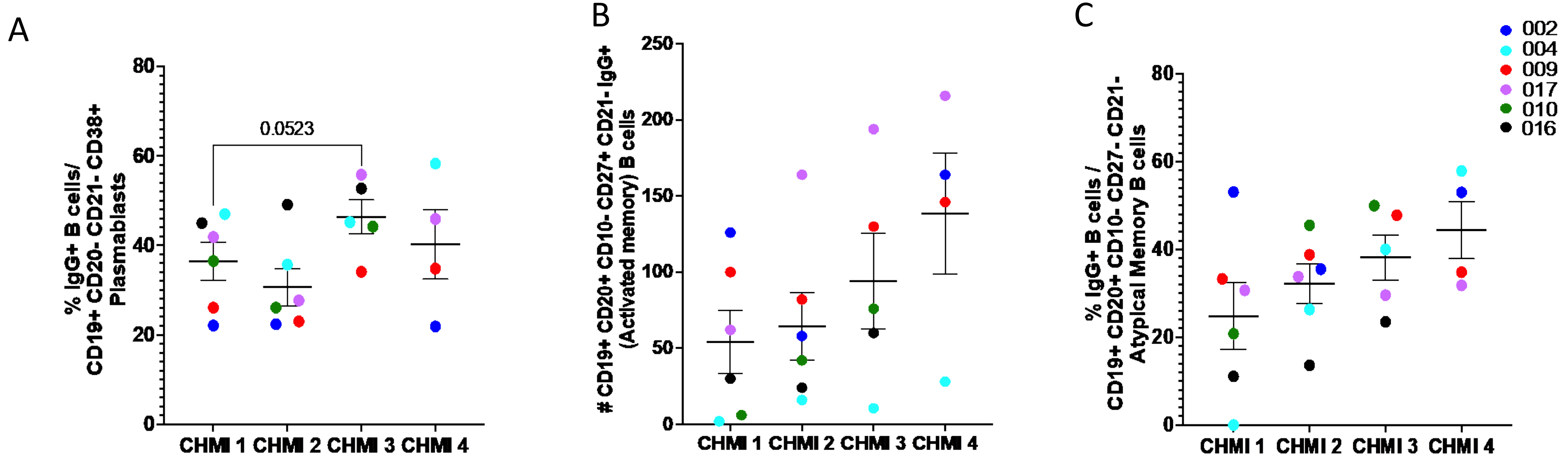

Supplementary Figure 3. B cell maturation through repetitive CHMI. PBMC from each participant collected 7 days after treatment (DRx + 7) from each CHMI were analyzed by flow cytometry without exogenous stimulation. A) Plots represent percent of IgG+ plasmablasts (CD19+CD20-CD21-CD38+) (A), activated memory cells (CD19+CD20+CD27 + CD21-) (B) or atypical memory B cells (CD19+CD20+CD10-CD27-CD21-) (C) for the 6 participants that completed the mock CHMI (CHMI1 n= 6, CHMI2 n= 6, CHMI3 n= 5, CHMI4 n=4). Means and standard error of the mean (SEM) at each time is indicated. Statistical differences were determined using Dunnett's multiple comparisons test between CHMI 1 and CHMI 2-4. Data that did not pass the normality test, was first logarithmically transformed to conform to normality. All P values are two-sided and source data are provided as a Source Data file.

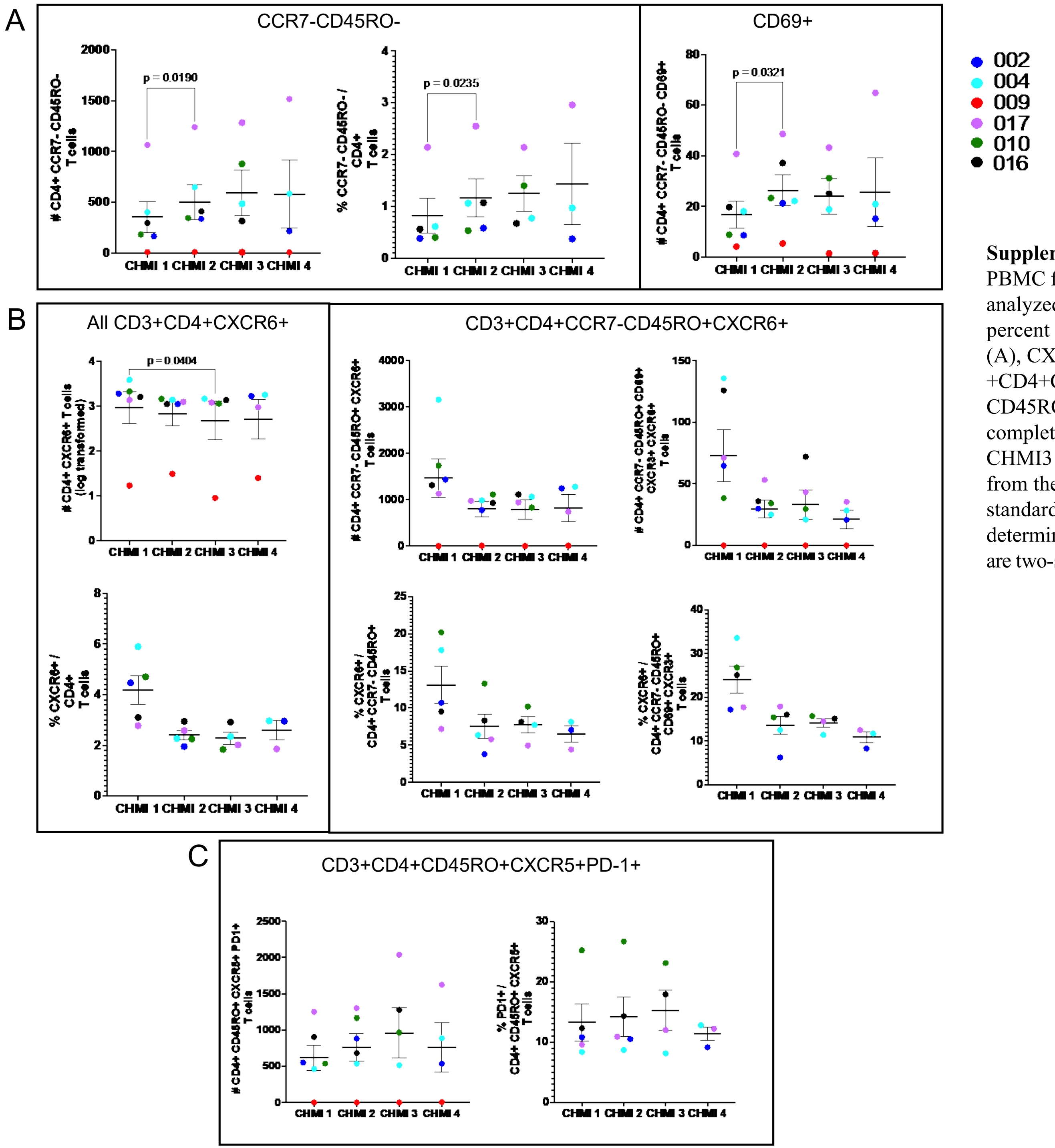

Supplementary Figure 4. CD4+CXCR6+ T cell populations decrease after repetitive CHMI. PBMC from each participant collected 7 days after treatment (DRx + 7) from each CHMI were analyzed by flow cytometry without exogenous stimulation. The number per 200,000 total cells and percent of CD3+CD4+CCR7-CD45RO- (effector) T cells and the subpopulation expressing CD69+ (A), CXCR6 positive CD3+CD4+ T cells, $CD3^{+}CD4^{+}CCR7^{-}CD45RO^{+}$ (effector memory) and CD3 +CD4+CCR7-CD45RO+CD69+CXCR3+ (activated effector memory) T cells (B), and CD3+CD4+ CD45RO+CXCR5+PD-1+ (T follicular helper) cells (C) are shown. All six participants that completed the mock CHMI were included in the flow cytometry analysis (CHMI1 n= 6, CHMI2 n= 6, CHMI3 n= 5, CHMI4 n=4), but the participant with a low CD3+CD4+ T cells count was excluded from the percent calculations (CHMI1 n= 5, CHMI2 n= 5, CHMI3 n= 4, CHMI4 n=3). Means and standard error of the mean (SEM) are shown for each cell type. Statistical differences were determined using Dunnett's multiple comparisons test between CHMI 1 and CHMI 2-4. All P values are two-sided and source data are provided as a Source Data file.

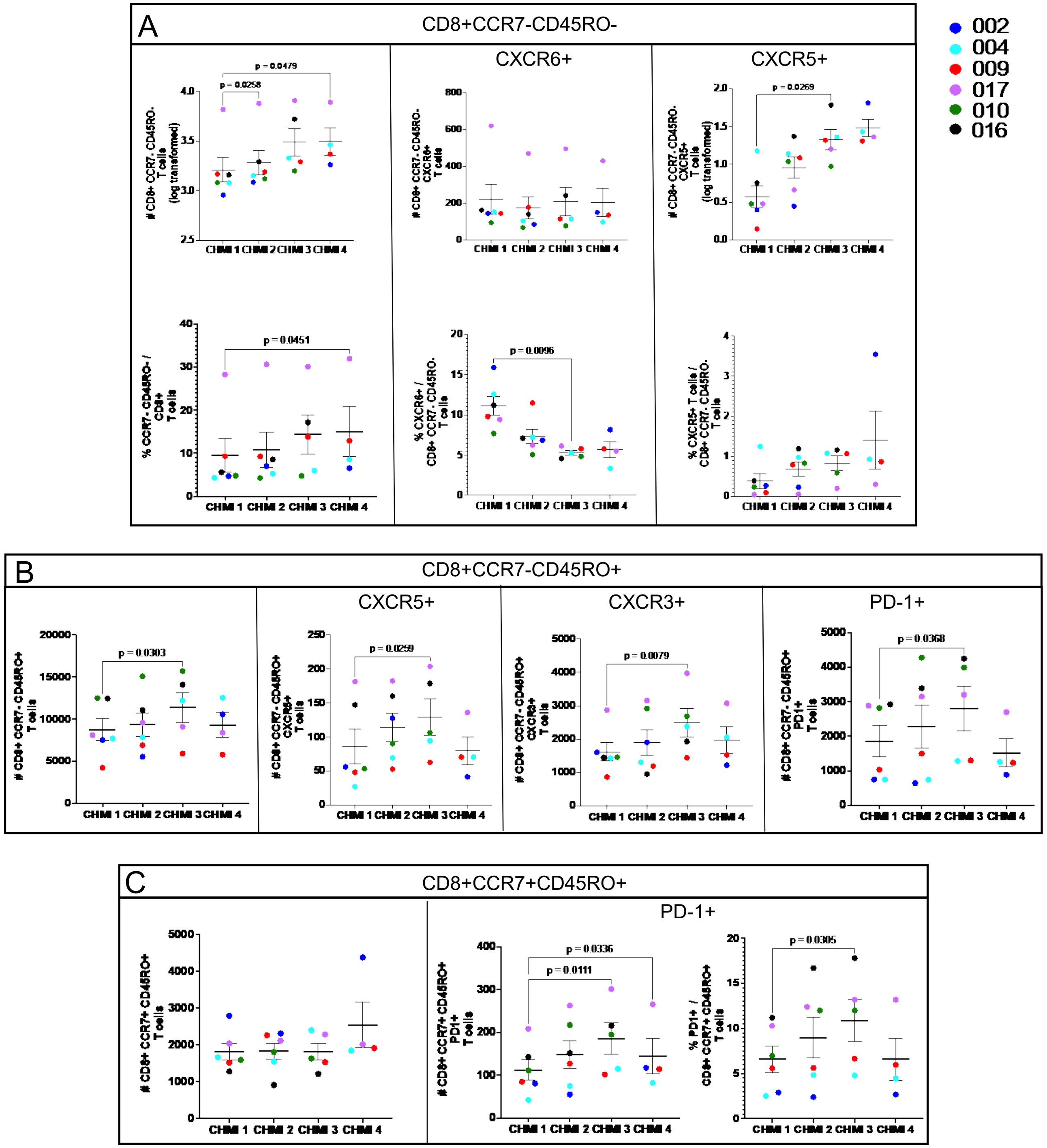

Supplementary Figure 5. CD8+ CCR7-CD45RO- (effector) and CCR7-CD45RO+ (effector memory) T cells increased after repetitive CHMI. The number per 200,000 total cells and percent of CD3+CD8+ $CCR7^{-}CD45RO^{-}$ T cells and the CXCR6+ and CXCR5+ subpopulations (A), the number of CD3+CD8+CCR7-CD45RO+ T cells and the CXCR5+, CXCR3+ and PD-1+ subpopulations (B), the number of CD3+CD8+CCR7+CD45RO T cells and the PD-1+ subpopulations (C) detected in the peripheral blood mononuclear cells (PBMC) collected on day 7 after treatment (DRx+7) after each of the controlled human malaria infections is shown. Samples from the 6 participants that completed the mock CHMI were included in the analysis (CHMI1 n= 6, CHMI2 n= 6, CHMI3 n= 5, CHMI4 n=4). Means and standard error of the mean (SEM) are shown for each cell type. Statistical differences were determined using Dunnett's multiple comparisons test between CHMI 1 and CHMI 2-4 and all P values were two-sided. Data that did not pass the normality test was first logarithmically transformed to conform to normality. Source data are provided as a Source Data file.

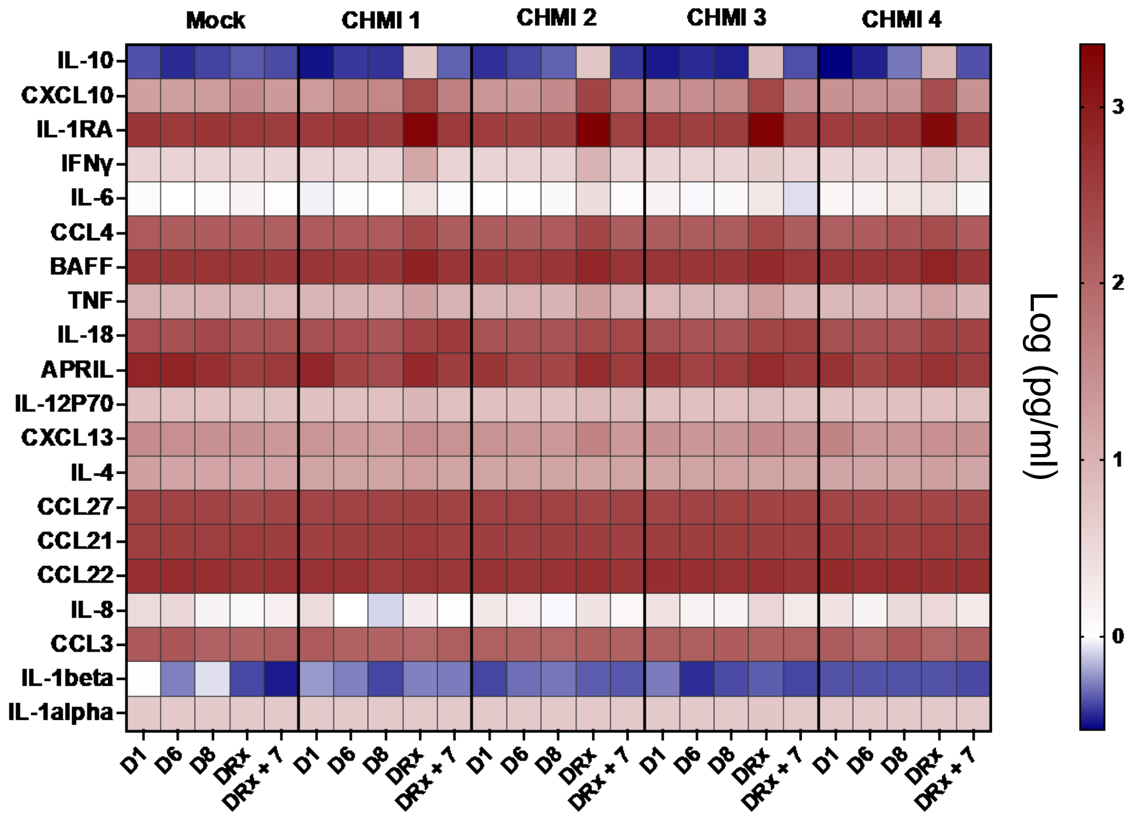

Supplementary Figure 6. Cytokines levels from the start of the mock mosquito challenge through the 4th exposure to Pf-infected mosquitoes. Plasma cytokine levels were measured by multiplex bead analysis for each participant (CHMI 1 n= 8, CHMI 2 n= 8, CHMI 3 n= 8, CHMI 4 n=5) on D1 before exposure to mosquitoes, D6, D8, D13 and D20 or on day of treatment (DRx) and 7 days after treatment (DRx+7) for each CHMI. A heatmap of the average cytokine concentration (pg/ml) on the indicated day is shown for all participants and source data are provided as a Source Data file.

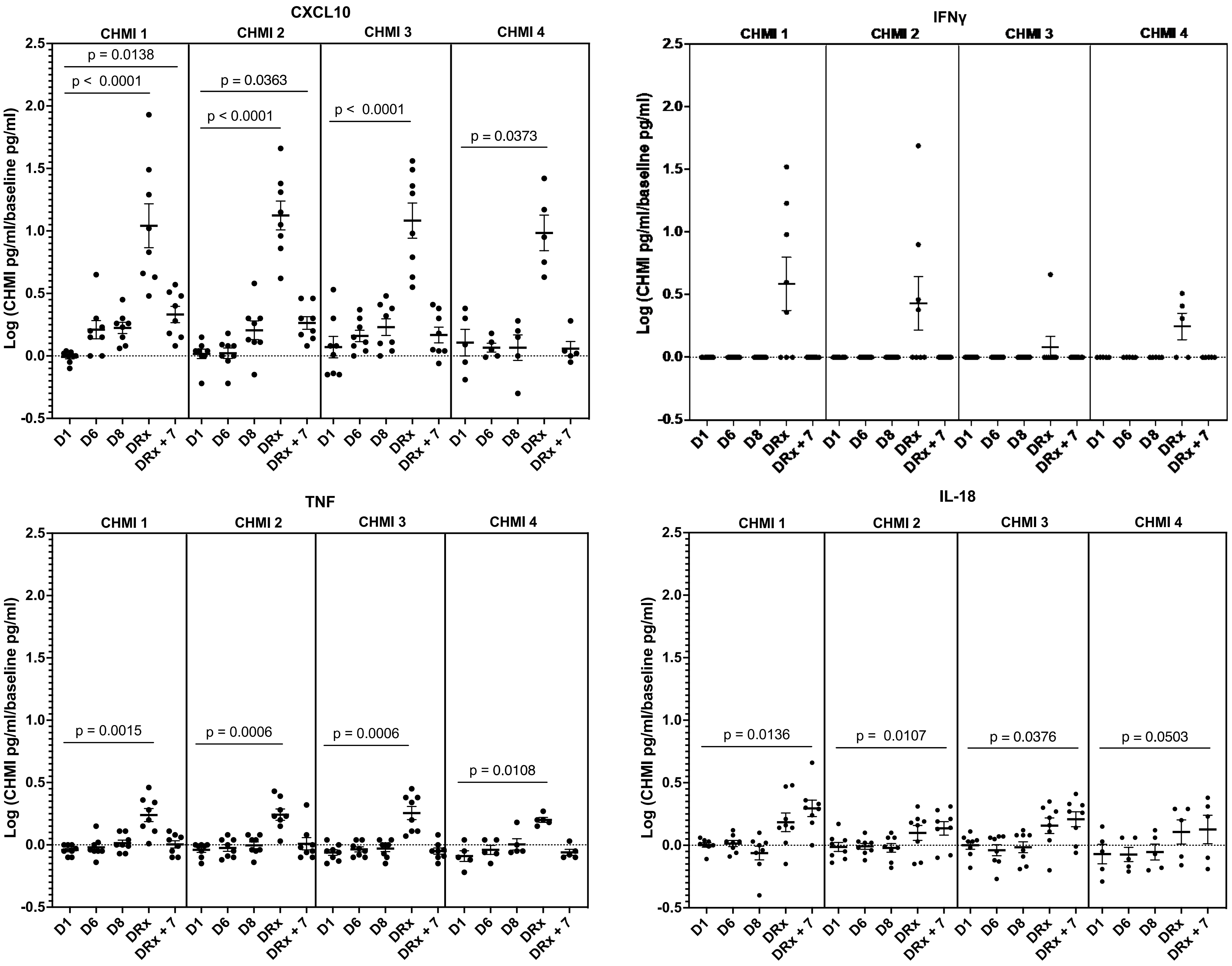

Supplementary Figure 7. Cytokine level time course Plasma cytokine levels were measured by multiplex bead analysis for each participant (CHMI1 n= 8, CHMI2 n= 8, CHMI3 n= 8, CHMI4 n=5) on D1 before exposure to Pf-infected mosquitoes, D6, D8, on day of treatment (DRx) and 7 days after treatment (DRx + 7) for each CHMI. Log scale plots of the ratios of the CXCL10, TNF, IL-18 and IFNγ levels at each time point to the baseline concentration during the mock CHMI (CHMI pg/ml)/(baseline cytokine pg/ml) for each participant are shown. Data are presented as mean and standard error of the mean (SEM) of 2-3 separate multiplex assays. Statistical differences were determined relative to D1 for each CHMI using Dunn's multiple comparisons test for CXCL10, IFNγ and TNF and Dunnett's multiple comparisons test for IL-18, which was normally distributed. All P values are two-sided and source data are provided as a Source Data file.

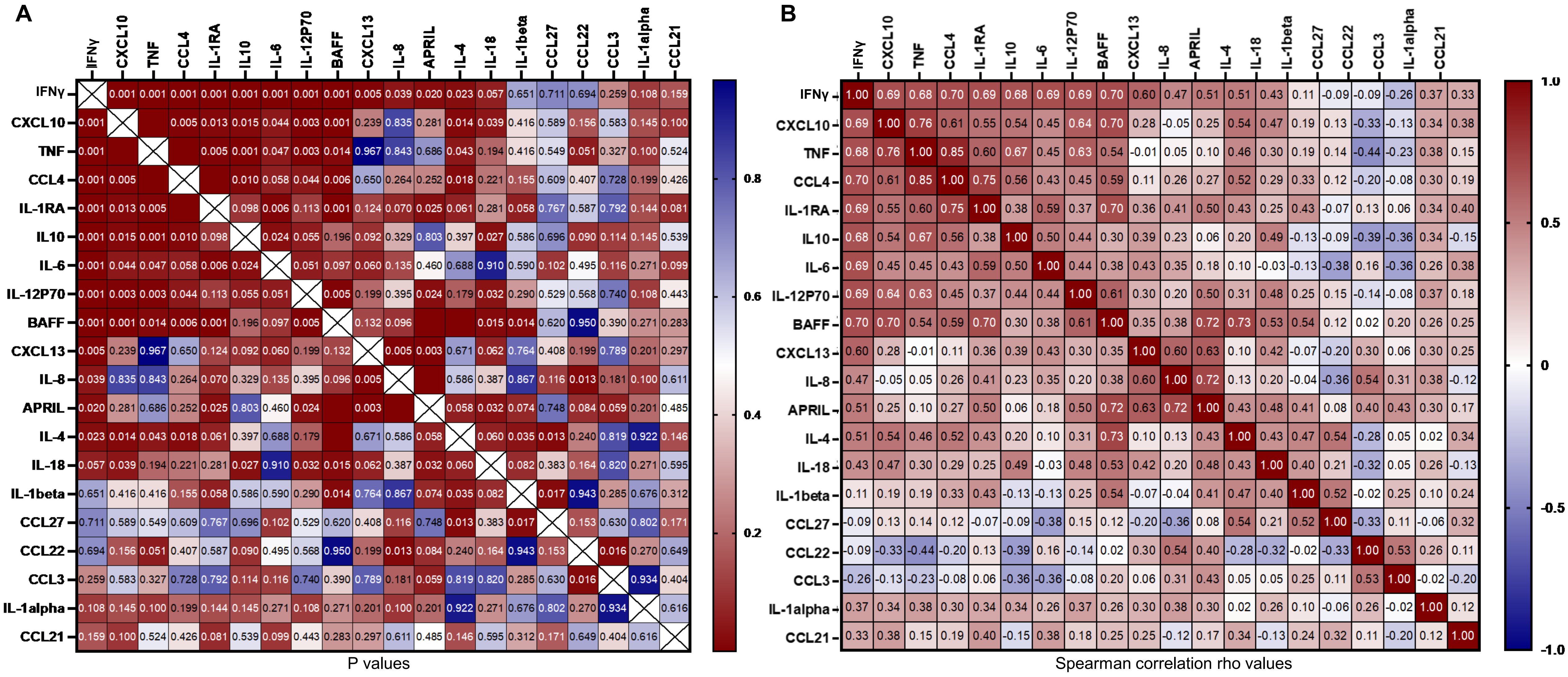

Supplementary Figure 8. Correlations between cytokines. The Spearman Correlation analysis of the plasma cytokine levels measured by multiplex bead analysis for each participant (CHMI1 n= 8, CHMI2 n= 8, CHMI3 n= 8, CHMI4 n=5) on D1 before exposure to Pf-infected mosquitoes, D6, D8, on day of treatment (DRx) and 7 days after treatment (DRx + 7) for each CHMI is shown. The P values (A) and Spearman correlation coefficients (B) are plotted separately. Source data are provided as a Source Data file.

	Parasitemia		Temperature	
	rho	P value	rho	P value
CXCL10	0.7964	<0.0001	0.4666	0.0002
IL-10	0.7561	<0.0001	0.486	0.0001
IL-1RA	0.7524	<0.0001	0.6197	<0.0001
TNF	0.7248	<0.0001	0.4015	0.0018
APRIL	0.6902	<0.0001	0.6646	<0.0001
BAFF	0.6516	<0.0001	0.3811	0.0032
CCL4	0.6083	<0.0001	0.6579	<0.0001
IFNγ	0.5303	<0.0001	0.3484	0.0074
IL-18	0.5247	<0.0001	0.5208	<0.0001
CXCL13	0.3022	0.0212	0.1138	0.3950
IL-12	0.2599	0.0488	0.3269	0.0122
IL-6	0.2425	0.0667	0.0564	0.6739
IL-8	0.2174	0.1012	0.2453	0.0634

Supplementary Fig 9. Cytokine levels are positively correlated with parasitemia and temperature. A) The correlations of the plasma cytokine levels (pg/ml) on D8 and on treatment day for each of the 8 participants with either their corresponding parasitemia (log scale) or the participant's maximum temperature after each challenge. Spearman's rank correlation was used for the analysis (rho value = correlation coefficient). Source data are provided as a Source Data file.

A

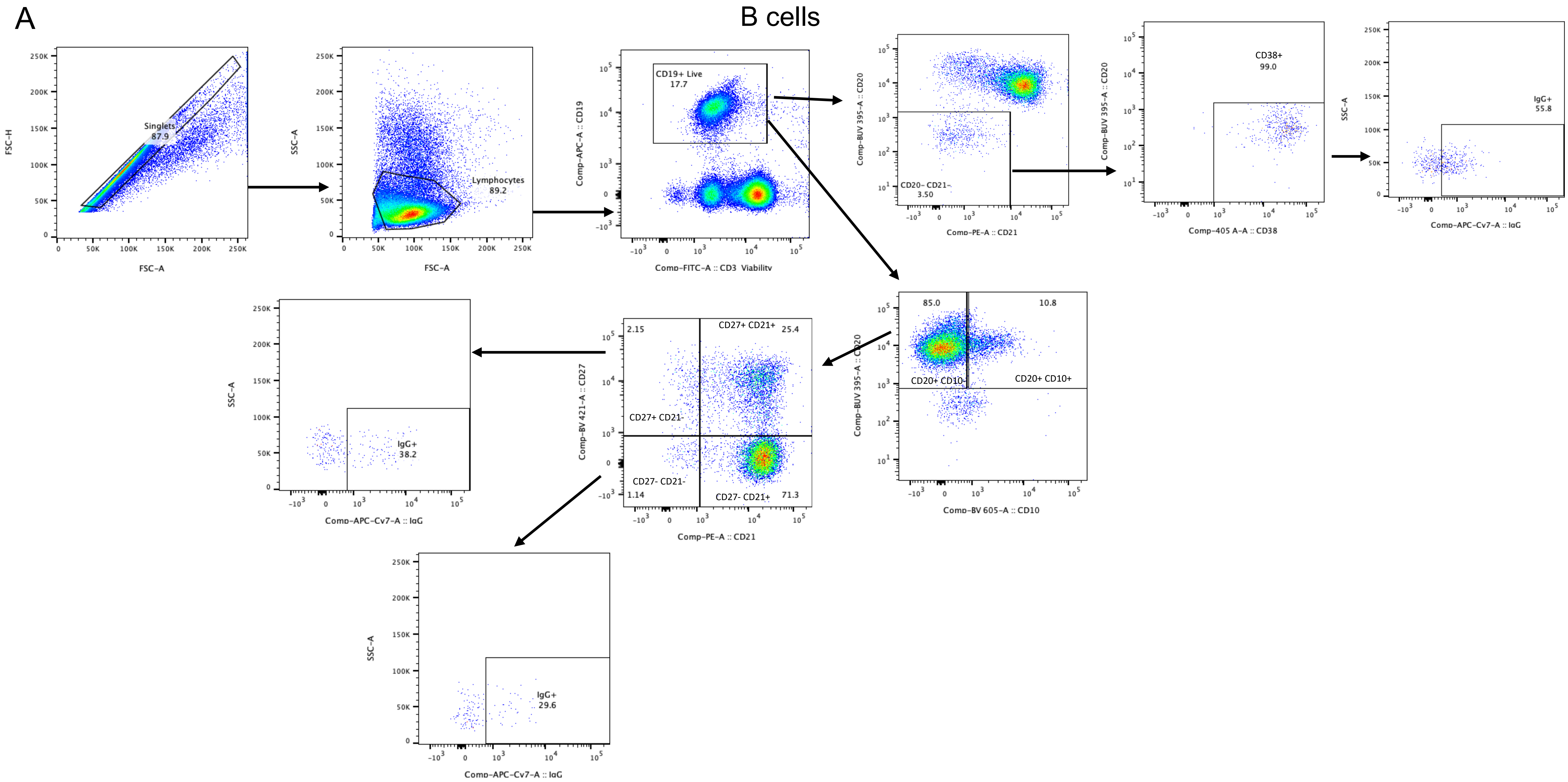

Supplementary figure 10: **Flow cytometry gating strategies**. Plots from one subject after CHMI 3 are shown to represent the gating strategy used to for B cells (A), T cells (B), CD4+ T cells (C) and CD8+ T cells (D).

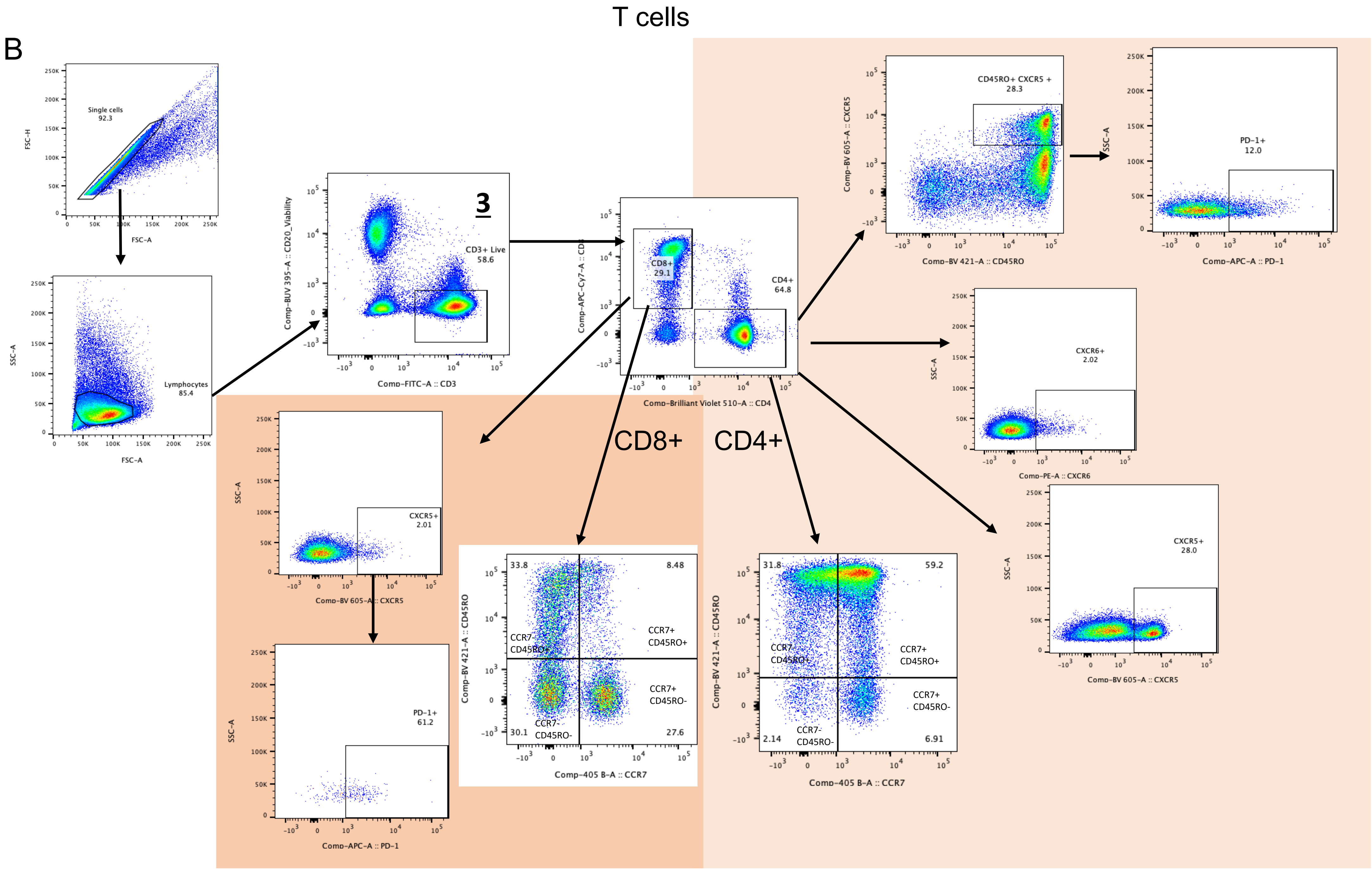
T cells
B
Single cells
92.3
FSC-H
FSC-A
Lymphocytes
85.4
SSC-A
FSC-A
3
CD3+ Live
58.6
Comp-BUV 395-A :: CD20_Viability
Comp-FITC-A :: CD3
CD8+
29.1
CD4+
64.8
Comp-Brilliant Violet 510-A :: CD4
CD45RO+ CXCR5 +
28.3
Comp-BV 605-A :: CXCR5
Comp-BV 421-A :: CD45RO
PD-1+
12.0
SSC-A
Comp-APC-A :: PD-1
CXCR6+
2.02
SSC-A
Comp-PE-A :: CXCR6
CXCR5+
28.0
SSC-A
Comp-BV 605-A :: CXCR5
CD8+
CD4+
CXCR5+
2.01
SSC-A
Comp-BV 605-A :: CXCR5
PD-1+
61.2
SSC-A
Comp-APC-A :: PD-1
33.8
8.48
CCR7-
CD45RO+
CCR7+
CD45RO+
CCR7+
CD45RO-
CCR7-
CD45RO-
30.1
27.6
Comp-BV 421-A :: CD45RO
Comp-405 B-A :: CCR7
31.8
59.2
CCR7-
CD45RO+
CCR7+
CD45RO+
CCR7+
CD45RO-
CCR7-
CD45RO-
2.14
6.91
Comp-BV 421-A :: CD45RO
Comp-405 B-A :: CCR7

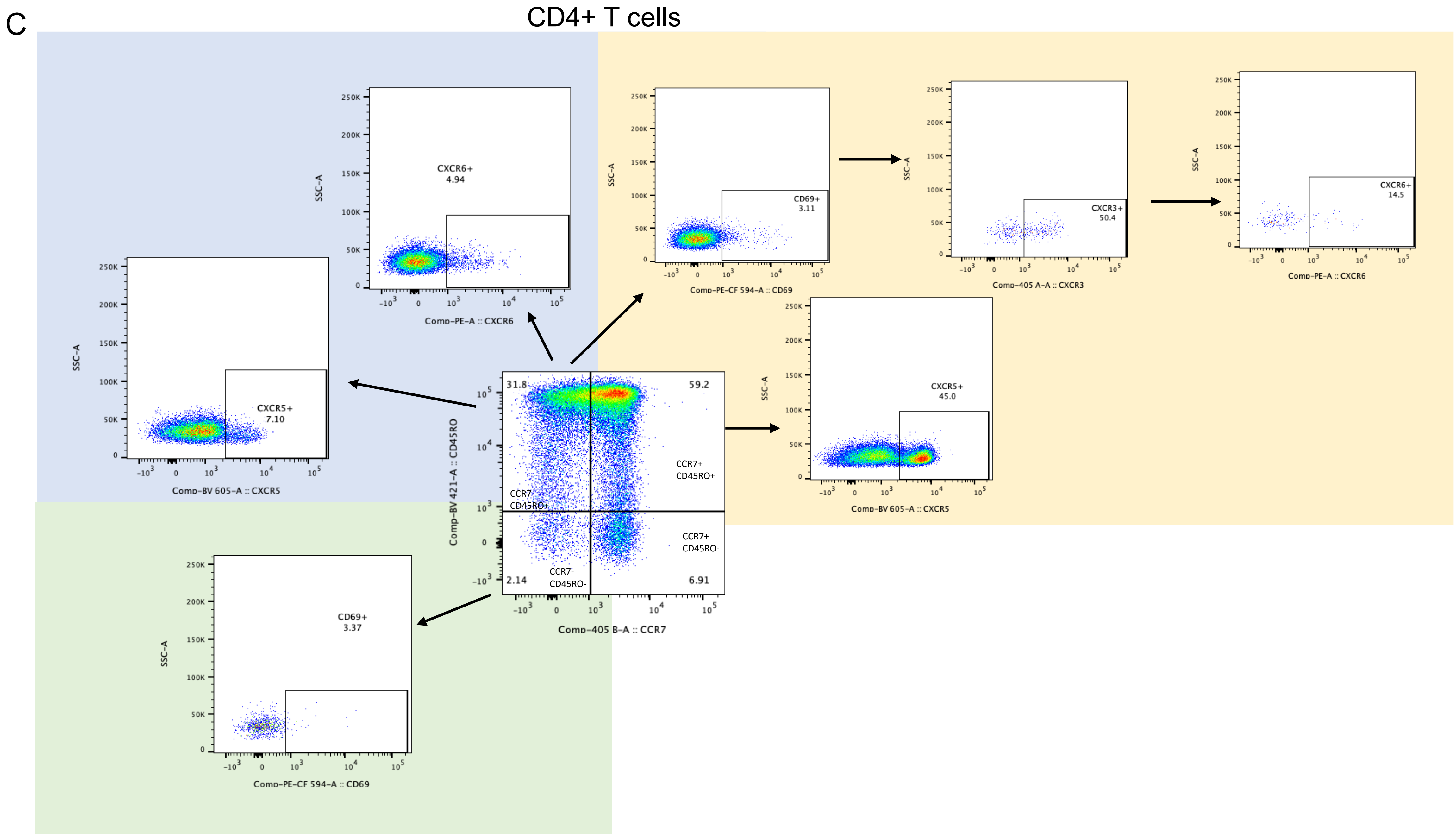
C
CD4+ T cells
31.8
59.2
2.14
6.91
CCR7- CD45RO+
CCR7+ CD45RO+
CCR7- CD45RO-
CCR7+ CD45RO-
Comp-BV 421-A :: CD45RO
Comp-405 B-A :: CCR7
CXCR6+ 4.94
Comp-PE-A :: CXCR6
CXCR5+ 7.10
Comp-BV 605-A :: CXCR5
CD69+ 3.37
Comp-PE-CF 594-A :: CD69
CD69+ 3.11
Comp-PE-CF 594-A :: CD69
CXCR3+ 50.4
Comp-405 A-A :: CXCR3
CXCR6+ 14.5
Comp-PE-A :: CXCR6
CXCR5+ 45.0
Comp-BV 605-A :: CXCR5
SSC-A

D

CD8+ T cells

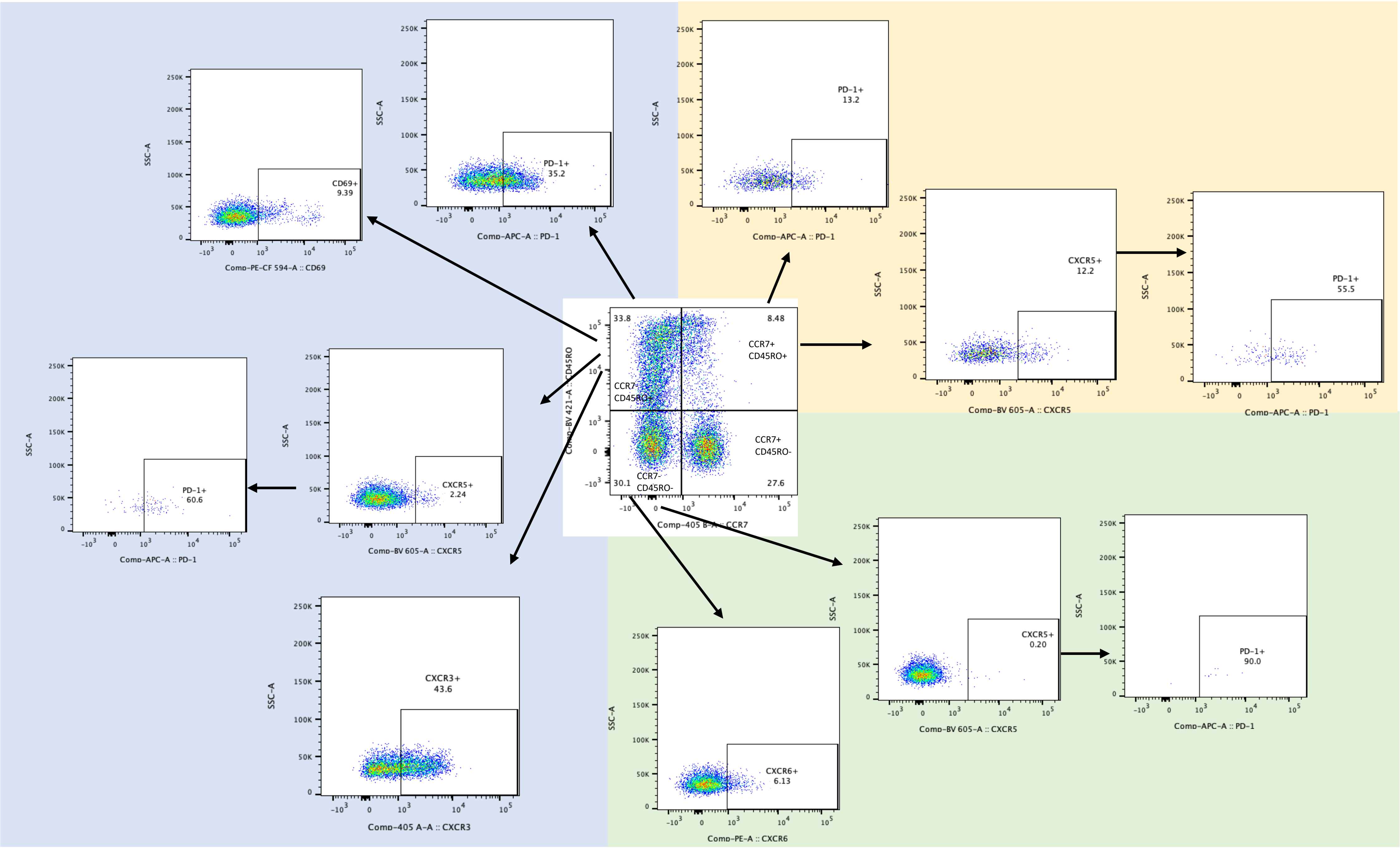
33.8
8.48
CCR7+
CD45RO+
CCR7-
CD45RO+
CCR7+
CD45RO-
CCR7-
CD45RO-
30.1
27.6
Comp-405 B-A :: CCR7
Comp-BV 421-A :: CD45RO
CD69+
9.39
Comp-PE-CF 594-A :: CD69
PD-1+
35.2
Comp-APC-A :: PD-1
PD-1+
13.2
CXCR5+
12.2
Comp-BV 605-A :: CXCR5
PD-1+
55.5
CXCR5+
2.24
PD-1+
60.6
CXCR3+
43.6
Comp-405 A-A :: CXCR3
CXCR6+
6.13
Comp-PE-A :: CXCR6
CXCR5+
0.20
PD-1+
90.0
SSC-A

Inclusion and Exclusion Criteria

Subject Inclusion Criteria

Both repeat CHMI subjects and the infectivity controls meeting all of the following inclusion criteria will be eligible to participate in this study: Refer to Appendix B in the protocol for reference ranges for normal laboratory values.

1. Male or non-pregnant/non-lactating female between the ages of 18 and 50 years, inclusive.
2. Able and willing to participate for the duration of the study.
3. Able to provide proof of identity to the satisfaction of the study clinician completing the enrollment process.
4. Able and willing to complete the informed consent process.
5. Willing to donate blood for sample storage to be used for future research (Note: refusal to allow future use is exclusionary).
6. Willing to refrain from blood donation to blood banks for 3 years following P. falciparum CHMI.
7. Agrees not to travel to a malaria endemic region during the entire course of study participation.
8. Physical examination and laboratory results without clinically significant findings and a body mass index (BMI) ≤35.

Laboratory Criteria within 56 days prior to enrollment:

9. Hemoglobin ≥11.2 g/dL for women; ≥12.6 g/dL for men.
10. Platelet count within institutional normal range
11. Alanine aminotransferase (ALT) ≤ upper limit of normal
12. Serum creatinine ≤ upper limit of normal.
13. Negative for HIV and Hepatitis B/C infection.

Laboratory Criterion documented any time prior to enrollment:

14. Negative sickle cell screening test.

Female-Specific Criteria:

15. Negative β-HCG pregnancy test (serum) on day of screening or urine pregnancy test at subsequent time points for women of childbearing potential.
16. Women of childbearing potential (exclusive of women in a same sex relationship) must agree to use effective means of birth control.*

* (e.g. oral or implanted contraceptives, IUD, female condom, diaphragm with spermicide, cervical cap, abstinence, use of a condom by the sexual partner or sterile sexual partner). Women with a history of amenorrhea (> 1 year duration) or surgical or chemical sterilization (e.g. tubal ligation, hysterectomy, other) must provide written documentation of infertility from a health care provider).

Subject Exclusion Criteria

Subjects meeting any of the following exclusion criteria will be excluded from study participation.

1. Women who are breast-feeding or planning to become pregnant during the time interval needed to complete the study.
2. Receipt of a malaria vaccine in a prior clinical trial.
3. Any history of malaria infection.
4. Evidence of increased cardiovascular disease risk; defined as >10% five year risk by the non-laboratory method.
5. Current use of systemic immunosuppressant pharmacotherapy.
6. History of a splenectomy, sickle cell disease or sickle cell trait.
7. Known history of anaphylactic response to mosquito-bites; or known allergy to artemether lumefantrine or atovaquone or proguanil or other component of the product.
8. Participation in any study involving investigational vaccine or drug within 4 weeks prior to enrollment, or expects to receive vaccine or drug during the 2-month post-challenge period.
9. Use or planned use of any drug with anti-malarial activity that would coincide with challenge.
10. Anticipated use of medications known to cause drug reactions with atovaquone-proguanil (Malarone®)

such as cimetidine, metoclopramide, antacids, and kaolin.

11. Plans to undergo surgery (elective or otherwise) between enrollment and 4 weeks (28 days) after any of the challenges.
12. Received a licensed vaccine within 1 month prior to enrollment in this study or expects to receive one during the 28 day post challenge period.
13. History of psychiatric disorders or behavioral tendencies (including active alcohol or drug abuse) that in the opinion of the investigator would make compliance with the protocol difficult*

*Medical and psychiatric illness defined as personality disorders, anxiety disorders, or schizophrenia or social condition, occupational reason or other responsibility that, in the judgment of the investigator, is a contraindication to protocol participation or impairs a volunteer's ability to give informed consent or to comply with the protocol schedule.

Justification for Exclusion of Pregnant Women and Children
Malaria infection during pregnancy can have adverse effects on both mother and fetus, including maternal anemia, fetal loss, premature delivery, intrauterine growth retardation and delivery of low birth-weight infants. Additionally, the inherent risk of a malaria infection would exclude persons < 18 years of age. Women who are pregnant or plan to become pregnant during the study period and persons <18 years of age are excluded from the study.

A systems biology approach to malaria immunity

Repetitive Controlled Human Malaria Infection (CHMI) Study in Malaria-Naïve Adults using NF54 strain Plasmodium falciparum (Pf)

DMID Protocol Number: 15-0058

DMID Funding Mechanism: *Grant # U01-AI-110852*

Other Identifying Numbers:

IND Sponsor: DMID

Grant Principal Investigator: Kim C. Williamson, PhD

Clinical Site Principal Investigator: Kirsten E. Lyke, M.D.

DMID Medical Monitor: Mo Elsafy, MD

DMID Clinical Project Manager: Effie Nomicos, MSN CCRP

DMID Regulatory Affairs Specialist: Blossom Smith, MS

Draft or Version Number: 9.0

12 May 2021

STATEMENT OF COMPLIANCE

This study will be carried out in accordance with the US Code of Federal Regulations (CFR), local regulations, and Good Clinical Practice (GCP) as required by the following:

- 45 CFR 46; 21 CFR 50, 21 CFR 56, and 21 CFR 11, 21 CFR 812, 21 CFR 312
- International Conference on Harmonization (ICH E6); 62 Federal Register 25691 (1997)
- All individuals responsible for the design and conduct of this study have completed Human Participants Protection Training and are qualified to be conducting this research prior to the enrollment of any participants. CVs for all investigators and sub-investigators participating in this trial are on file in a central facility (21 CFR 312.23 [a] [6] [iii] [b] edition).

The signature on the following page constitutes approval of this protocol and the attachments, and provides the required assurances that this trial will be conducted according to all stipulations of the protocol, including all statements regarding confidentiality, and according to local legal and regulatory requirements, applicable US federal regulations and (ICH E6) guidelines.

SIGNATURE PAGE

The signature below constitutes the approval of this protocol and the attachments, and provides the necessary assurances that this trial will be conducted according to all stipulations of the protocol, including all statements regarding confidentiality, and according to local legal and regulatory requirements and applicable US federal regulations and ICH guidelines.

Clinical Investigator:

Signed: ____ Date: _________________

Name: Kirsten E. Lyke, MD
Title: Professor of Medicine

TABLE OF CONTENTS

Statement of Compliance ii
Signature Page iii
Table of Contents iv
List of Abbreviations vii
Protocol Summary ix
1 Key Roles 13
2 Background Information and Scientific Rationale 16
  2.1 Background Information 16
    2.1.1 Background and Rationale of Controlled Human Malaria Infection Studies 16
    2.1.2 University of Maryland Malaria CHMI Experience 17
    2.1.3 Safety of the Controlled Human Malaria Infection by the Bite of Mosquito 18
  2.2 Rationale 20
    2.2.1 Acquisition of Malaria Immunity 20
    2.2.2 Transcription profiling and immunophenotyping 23
    2.2.3 Humoral Immune Response 23
    2.2.4 Summary 24
  2.3 Potential Risks and Benefits 25
    2.3.1 Potential Risks 25
    2.3.2 Known Potential Benefits 29
3 Objectives 30
  3.1 Study Objectives 30
    3.1.1 Primary Objective: 30
    3.1.2 Secondary Objectives: 30
    3.1.3 Exploratory Objectives: 30
  3.2 Study Outcome Measures 30
    3.2.1 Primary Outcome Measures 30
    3.2.2 Secondary Outcome Measures 31
    3.2.3 Exploratory Outcome Measures 31
4 Study Design 32
5 Study Enrollment and Withdrawal 35
  5.1 Subject Inclusion Criteria 36
  5.2 Subject Exclusion Criteria 37
    5.2.1 Justification for Exclusion of Pregnant Women and Children 37
  5.3 Treatment Assignment Procedures 38
    5.3.1 Randomization Procedures 38
    5.3.2 Masking Procedures 38
    5.3.3 Reasons for Withdrawal 38
    5.3.4 Handling of Withdrawals 39
    5.3.5 Termination of Study 40
6 Study Intervention/Investigational Product 41
  6.1 Study Product Description 41
    6.1.1 Acquisition 41
    6.1.2 Formulation, Packaging, and Labeling 41
    6.1.3 Product Storage and Stability 41

6.2 Dosage, Preparation and Administration of Study Intervention/Investigational Product 42
6.2.1 Storage and Stability (Shelf-life) 42
6.3 Modification of Study Intervention/Investigational Product for a Participant 42
6.4 Accountability Procedures for the Study Intervention/Investigational Product(s) 43
6.5 Assessment of Subject Compliance with Study Intervention/Investigational Product 43
6.6 Concomitant Medications/Treatments 43
7 Study Schedule 44
7.1 Screening 44
7.1.1 Repeat CHMI Subjects 44
7.1.2 Infectivity Control Subjects 44
7.2 Enrollment/Baseline 45
7.2.1 CHMI 45
7.3 Follow-up 46
7.3.1 CHMI and outpatient follow-up 46
7.3.2 Inpatient follow-up 46
7.3.3 Outpatient daily follow-up 47
7.3.4 Outpatient follow-up 47
7.3.5 Malaria Infection 48
7.3.6 CHMI Conclusion Visit 20 (+28 days post-therapy (+/- 3 days) 50
7.4 Final Study Visit 50
7.5 Early Termination Visit 50
7.6 Unscheduled Visit 51
8 Study Procedures/Evaluations 52
8.1 Clinical Evaluations 52
8.1.1 Screening and enrollment: 52
8.1.2 Subject follow-up: 54
8.2 Laboratory Evaluations 59
8.2.1 Clinical Laboratory Evaluations 59
8.2.2 Special Assays or Procedures 60
8.2.3 Specimen Preparation, Handling, and Shipping 62
9 Assessment of Safety 63
9.1 Specification of Safety Parameters 63
9.1.1 Primary safety measurements: 63
9.1.2 Adverse Events 63
9.1.3 Relationship to CHMI 64
9.1.4 Reactogenicity – solicited events related to administration of CHMI 64
9.1.5 Malaria events and Clinical malaria infections 65
9.1.6 Serious Adverse Events 65
9.1.7 Abnormal Laboratory Test Values 66
9.2 Timing for Assessing, Recording, and Analyzing Safety Parameters 66
9.2.1 Uninfected mosquito challenge (repeat CHMI cohort) 66
9.2.2 Infected mosquito challenge periods (repeat CHMI and Control Cohorts) .66
9.3 Reporting Procedures 67
9.3.1 Reporting of Serious Adverse Events 67
9.3.2 Regulatory Reporting for Studies Conducted Under DMID-Sponsored IND 68

9.3.3 Regulatory Reporting for Studies Not Conducted Under DMID-Sponsored IND 68
9.3.4 Other Adverse Events (if applicable) 68
9.3.5 Reporting of Pregnancy 68
9.4 Type and Duration of Follow-up of Subjects after Adverse Events 68
9.5 Halting Rules 69
9.6 Safety Oversight (ISM plus SMC) 70
9.6.1 Safety Monitoring Committee (SMC) 70
9.6.2 Independent Safety Monitor (ISM) 70
10 Clinical Monitoring 72
10.1 Site Monitoring Plan 72
11 Statistical Considerations 73
11.1 Study Hypotheses 73
11.2 Sample Size Considerations 73
11.3 Planned Interim Analyses (if applicable) 73
11.3.1 Safety Review 73
11.3.2 Efficacy Analysis 73
11.4 Final Analysis Plan 74
11.4.1 Demographic Summaries 74
11.4.2 Safety Analysis 74
11.4.3 Protection against *P. falciparum* parasites (Primay Objective) 74
11.4.4 Immune response analysis (Secondary Objective) 75
12 Source Documents and Access to Source Data/Documents 79
13 Quality Control and Quality Assurance 80
14 Ethics/Protection of Human Subjects 81
14.1 Ethical Standard 81
14.2 Institutional Review Board 81
14.3 Informed Consent Process 81
14.3.1 Informed Consent/Assent Process (in Case of a Minor) 82
14.4 Exclusion of Women, Minorities, and Children (Special Populations) 83
14.5 Subject Confidentiality 83
14.6 Study Discontinuation 83
14.7 Future Use of Stored Specimens 84
15 Data Handling and Record Keeping 85
15.1 Data Management Responsibilities 85
15.2 Data Capture Methods 86
15.3 Types of Data 86
15.4 Timing/Reports 86
15.5 Study Records Retention 86
15.6 Protocol Deviations 86
16 Publication Policy 88
17 Literature References 89
Supplements/Appendices 96
Appendix A: Informed Consent quiz 97
Appendix B: Toxicity Table 100
Appendix C: Schedule of Events 103
Appendix D. Brighton Case definition of Anaphylaxis[74] 110

LIST OF ABBREVIATIONS

Ab	Antibodies
ACT	Artemisinin-based Combination Therapy
Ad	Adenovirus
AE	Adverse Event/Adverse Experience
CFR	Code of Federal Regulations
CHI	Center for Human Immunology, Autoimmunity and Inflammation
CHMI	Controlled Human Malaria Infection
CMI	Cell Mediated Immunity
CONSORT	Consolidated Standards of Reporting Trials
CRF	Case Report Form
CQ	Chloroquine
CQMP	Clinical Quality Management Plan
CROMS	Clinical Research Operations Management and Support
CS	Circumsporozoite
Ct	Cycle threshold
CVD	Center for Vaccine Development
DHHS	Department of Health and Human Services
DMID	Division of Microbiology and Infectious Diseases, NIAID, NIH,
ECG	Electrocardiogram
eCRF	Electronic Case Report Form
EDC	Electronic Data Capture
ELISA	Enzyme-linked immunosorbent assay
FDA	Food and Drug Administration, DHHS
FWA	Federal wide Assurance
GCP	Good Clinical Practice
HLA	Human Leukocytic Antigen
IATA	International Air Transport Association
IB	Investigator's Brochure
ICF	Informed Consent Form
ICH	International Conference on Harmonisation
ICMJE	International Committee of Medical Journal Editors
ICS	Intracellular Cytokine Secretion
ID_{100}	100% Infective Dose
IEC	Independent or Institutional Ethics Committee
IND	Investigational New Drug Application
IRB	Institutional Review Board
ISM	Independent Safety Monitor
LFT	Liver Function Test
LLN	Lower Limit of Normal
MedDRA ®	Medical Dictionary for Regulatory Activities
MHC	Major Histocompatibility Complex
MOP	Manual of Procedures
N	Number (typically refers to number of participants)

NF54	*P. falciparum* strain NF54
NIAID	National Institute of Allergy and Infectious Diseases, NIH,
NIH	National Institutes of Health, DHHS
NLM	National Library of Medicine
NMRC	Naval Medical Research Center
OCRA	Office of Clinical Research Affairs, DMID, NIAID, NIH, DHHS
OHRP	Office for Human Research Protections, DHHS
OHSR	Office for Human Subjects Research, NIH, DHHS
ORA	Office of Regulatory Affairs, DMID, NIAID, NIH, DHHS
PCR	Polymerase Chain Reaction
Pf	*Plasmodium falciparum*
PfSPZ	*Plasmodium falciparum* Sporozoites
PI	Principal Investigator
PE	Physical Examination
PBMC	Peripheral Blood Mononuclear Cells
QA	Quality Assurance
QC	Quality Control
QM	Quality Management
RUNMC	Radboud University Nijmegen Medical Center
SAE	Serious Adverse Event/Serious Adverse Experience
SDCC	Statistical and Data Coordinating Center
SMC	Safety Monitoring Committee
SOP	Standardized Operating Procedure
SPZ	Sporozoites
ULN	Upper Limit of Normal
US	United States
Us qPCR	Ultra-sensitive quantitative PCR
VTEU	Vaccine Training and Evaluation Unit
WRAIR	Walter Reed Army Institute of Research

PROTOCOL SUMMARY

**Title:**	A systems biology approach to malaria immunity: Repetitive Controlled Human Malaria Infection (CHMI) Study in Malaria-Naïve Adults using NF54 strain *Plasmodium falciparum* (Pf)
**Phase:**	Phase 1
**Population:**	Approximately 34 healthy male and female volunteers (including 10 active study volunteers and 18 naïve controls to confirm *Pf* infectivity during the 2nd -5th CHMI challenges) aged 18 to 50 years, inclusive, from the greater Baltimore community
**Number of Sites:**	Institute for Global Health, Center for Vaccine Development, University of Maryland School of Medicine, Baltimore, Maryland
**Study Duration:**	48 months
**Subject Participation Duration:**	Including a 2 month recruitment/screening period, repeat CHMI volunteers subjects will participate in the study for 36-48 months during which time they will be actively involved for a total of 10 months, 1 month for the screen, 1 month for the uninfectious challenge and 2 months for each of the 5 infectious challenges. The CHMI control volunteers will only participate in one CHMI which will take 3 months including an ~1 month screening period.
**Description of Agent or Intervention:**	**Sanaria® Challenge Mosquitoes** (The bite of five (5) non-aseptic *Anopheles stephensi* mosquitoes infected with *P. falciparum* NF54 strain sporozoites)
**Objectives:**	Primary:<br>Determine whether protective immunity against parasite infection develops following repeat CHMI<br>Secondary:<br>1. Determine whether clinical signs and symptoms of malaria decrease in intensity and duration following repeat CHMI.<br>2. Track the production of antibodies that react with *P. falciparum* sporozoites and blood stage parasites on Study days 6 and 8, treatment day and 7 days post treatment initiation or, if aparasitemic, Study Days 13 and 21 to compare responses over time and to sequential CHMI.

Exploratory:

1. Identify which PBMCs are activated and proliferate on Study days 6 and 8, treatment day and 7 days post-treatment initiation or, if aparasitemic, Study Days 13 and 21 to compare responses over time and to sequential CHMI.

2. Determine the RNA expression profile of blood cells collected on Study Days 1, 2 and every other day until 7 days after inpatient (or outpatient daily fu) PBMC collection to compare responses over time and to sequential CHMI.

3. Determine the plasmablast immunoglobulin gene repertoire 21 days after the uninfectious challenge and 7 days after the inpatient (or outpatient daily fu) PBMC collection to compare responses over time and to sequential CHMI.

Description of Study Design: This study will assess the acquisition of immunity to Pf malaria over the course of 5 sequential CHMI over 3-4 years, in 10 healthy adult participants. 10 subjects will initially be challenged with 5 uninfected mosquitoes (mock), followed by 5 challenges (CHMI 1-4) with 5 mosquitoes infected with drug sensitive, *P. falciparum* parasites (strain NF54) (e.g., 2, 8, 14-20, 20-32, and 32-36 months later). For the final four infective CMHIs (CHMI #s 2-4), six additional immunologic malaria-naïve subjects will be enrolled and challenged as infectivity controls. If dropouts occur within the original 10 person cohort, and two or more CHMI remain, back-up replacement volunteers will be recruited to undergo successive CHMI with the core group. The goal will be to determine if repeat CHMI induces immunity by monitoring clinical responses including pre-patent periods, parasitemia and clinical symptomatology during successive malaria episodes as well as to systematically examine immune responses over time in malaria-naive adult volunteers.

All volunteers (repeat CHMI subjects and infectivity controls) will be evaluated as part of an inpatient stay (or outpatient daily fu) to diagnose Pf malaria infection and treat with Coartem® (artemether/lumefantrine) or Malarone® (Atovaquone/proguanil). Daily observation will occur from Study Days 9-19 or until three-day directly observed therapy for *P. falciparum* infection is complete and two negative smears separated by a time interval >12 hours have been documented. A

	third negative smear >12 hours after the previous two daily smears will be documented to affirm malaria cure. This can be done as an outpatient. Infectivity Controls enrolled as part of CHMI #5 will be treated based on concomitant us qPCR results. Smears can be performed if necessary but the reliability and sensitivity of PCR allows for early (2-5 days) detection of malaria. Additional outpatient, post-malaria infection follow-up for all volunteers will occur 20 and 28 days after challenge and 28 days after treatment. The repeat CHMI subjects will have additional outpatient visits days 1, 3, 5, and 7 after the challenge (Study Days 2, 4, 6 and 8) to obtain blood samples to monitor the development of immunity.
**Estimated Time to Complete Enrollment:**	60 days

SCHEMATIC OF STUDY DESIGN

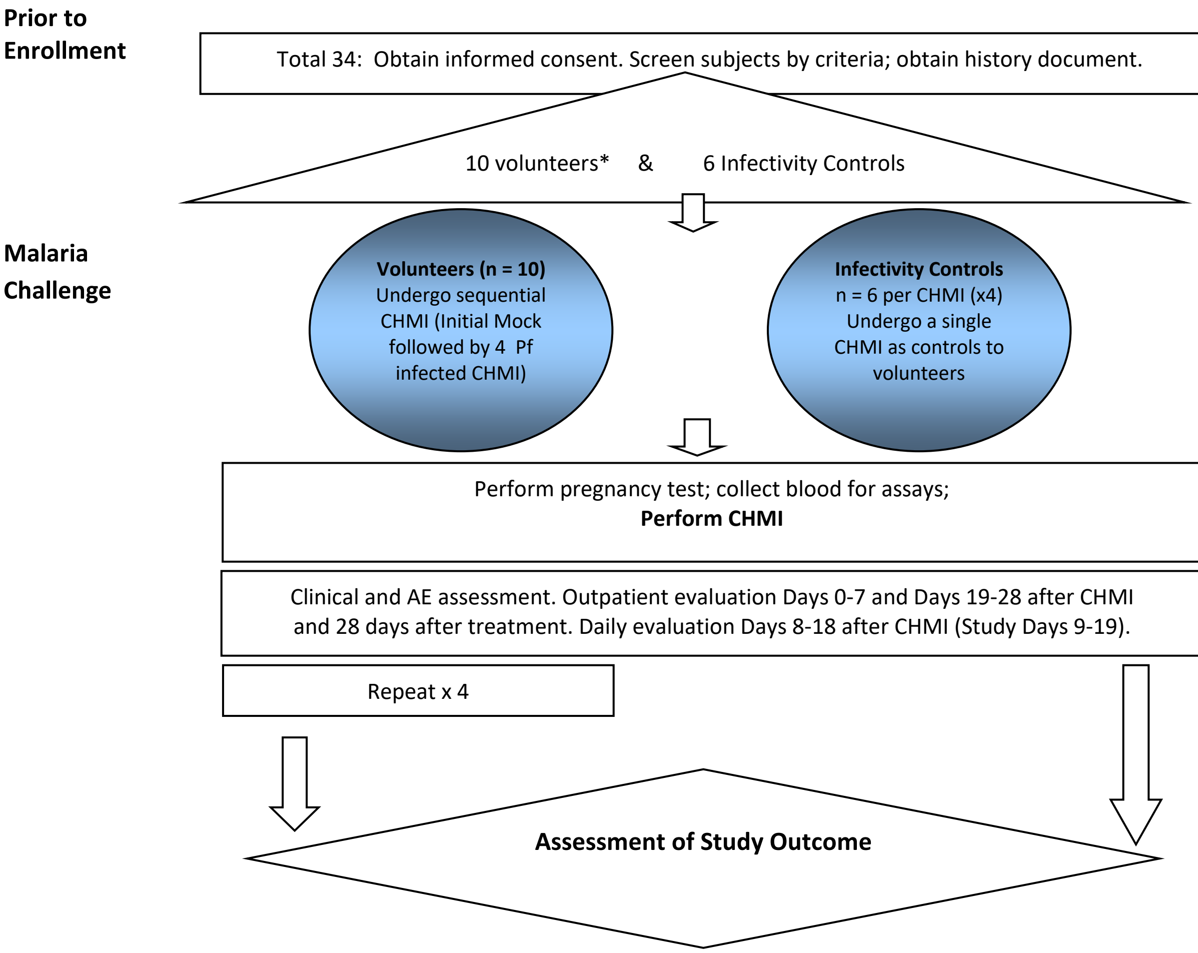

	**Recruit** Days -56-1	**Mock** Days 1-28	**CHMI 1** Months 2 & 3	**Recruit** Months 6 & 7	**CHMI 2** Months 8 & 9	**Recruit** 5-12 Mo post-CHMI 2	**CHMI 3** 6-12 Mo post-CHMI 2	**Recruit** 5-12 Mo post-CHMI 3	**CHMI 4** 6-12 Mo post-CHMI 3	**Recruit** 5-12 Mo post-CHMI 4	**CHMI 5** 6-12 Mo post-CHMI 4
**Repeat CHMI cohort (n=10)**	X	X	X		X		X		X		X
**Control cohort 1 (n=6)**				X	X						
**Control cohort 2 (n=6)**						X	X				
**Control cohort 3 (n=6)**								X	X	X	X

*Back-up volunteers can replace dropouts if ≥ 2 infected CHMI remain in study.

1 KEY ROLES

Individuals:

Grant Principal Investigator:

Kim Williamson, PhD
Professor of Microbiology and Immunology
Uniformed Services University of the Health Sciences
Department of Microbiology and Immunology
4301 Jones Bridge Road
Bethesda, Maryland 20814-4799
Phone: 301-295-3951
FAX: (301) 295-3773
Email: kim.williamson@usuhs.edu

Clinical Site Principal Investigator:

Kirsten E. Lyke, MD
Professor of Medicine
Director, Malaria Vaccine and Challenge Unit
Center for Vaccine Development and Global Health
University of Maryland School of Medicine
685 W Baltimore St, Room 480
Baltimore, MD 21201
Phone: 410 706 7376/0462
Fax: 410 706 1204
Email: KLyke@medicine.umaryland.edu

Clinical Project Manager:

Effie Y. H. Nomicos RN MSN CCRP
Parasitology and International Programs Branch (PIPB)
HHS/NIH/NIAID/DMID
Room 8A35, MSC 9825
5601 Fishers Lane
Bethesda, MD. 20892
Phone: 240-267-3329
Blackberry: 240-507-0343
Internet: enomicos@mail.nih.gov

Program Officer/Medical Officer:

Gregory Deye, MD
Parasitology & International Programs Branch (PIPB)
DMID
5601 Fishers Lane, Room 8A39, MSC 9825
Bethesda, MD 20892-9825 (Express delivery: Rockville, MD 20852)
Phone: 240-292-4199

E-mail: gregory.deye@nih.gov

Medical Monitor:

Mo Elsafy, MD, MSc
NIH/NIAID/DMID
5601 Fishers Lane
7E56 MSC 9826
Bethesda, MD 20852
Phone: 240-292-4241
Email: elsafymm@nih.gov

Statistical and Data Coordinating Center:

The Emmes Corporation
401 N. Washington St.
Rockville, MD 20850
Phone: 301-251-1161
Fax: 301-251-1355

Regulatory Affairs Specialist:

Blossom T. Smith, MS
Office of Regulatory Affairs
National Institute of Allergy & Infectious Diseases
National Institutes of Health
BG 5601FL RM 7F45
Mail Stop 9826
5601 Fishers Lane
Bethesda, MD 20852
Tel: 240-627-3376
E-mail: blossom.smith@nih.gov

Independent Safety Monitor:

Wilbur Chen, M.D.
Center for Vaccine Development and Global Health
University of Maryland School of Medicine
685 W. Baltimore St, Room 480
Baltimore, MD 21201
Phone: 410 706 7376
Fax: 410 706 1204
Email: WChen@medicine.umaryland.edu

Study agent provider and personnel:

Sanaria, Inc.
9800 Medical Center Drive, Ste A209
Rockville, MD 20850
Tel: 301-770-3222
Fax : 301-770-5554
Primary Contact : Anusha Gunasekera
E-mail : agunasekera@sanaria.com

Chief Executive and Scientific Officer : Stephen L. Hoffman, MD

E-mail: slhoffman@sanaria.com

IRB Contact:

Human Research Protections Office
University of Maryland, Baltimore
800 W. Baltimore Street, Suite 100
Tel: 410-706-5037
Fax: 410-706-4189
E-mail: HRPO@som.umaryland.edu

and

Human Research Protections Program Office
Uniformed Services University of the Health Sciences
4301 Jones Bridge Road
Room A2051
Bethesda, MD 20814
Phone: (301) 295-9534
E-mail: hrpo@umaryland.edu

Study Site:

Center for Vaccine Development and Global Health
University of Maryland School of Medicine
685 W. Baltimore St, HSF I, Room 480
Baltimore, MD 21201
Phone: 410 706 5328
Fax: 410 706 6205

Inpatient Facility:

Pharmaron Biomedical Laboratories
Clinical Pharmacology Center
University of Maryland BioPark Facility
800 W. Baltimore St., 5th Floor
Baltimore, MD 21201
Phone: 410-760-8812 Contact: Bridget McMahon, Director

Co-Investigator:

Andrea A. Berry, M.D.
Assistant Professor of Pediatrics and Medicine
Division of Infectious Diseases and Tropical Pediatrics
Center for Vaccine Development and Global Health
University of Maryland School of Medicine
685 W. Baltimore Street, Room 480
Baltimore, MD 21201
+1-410-706-1123 (office)
+1-410-706-6205 (fax)
aberry@som.umaryland.edu

2 BACKGROUND INFORMATION AND SCIENTIFIC RATIONALE

2.1 Background Information

2.1.1 Background and Rationale of Controlled Human Malaria Infection Studies

Controlled human malaria infection studies (formerly termed malaria challenge trials) have been used as a powerful tool to evaluate malaria vaccine and prophylactic drug efficacy since the 1980s, generating extensive safety datasets. Step by step improvements in the partially protective vaccine based upon the *Plasmodium falciparum* (Pf) circumsporozoite protein (RTS,S), have been made using data acquired from the challenge model.[1-3] Controlled human malaria infection (CHMI) provides the data for or against further clinical development of candidate vaccines, including the decision to test in a pediatric population. Both negative and positive results of malaria challenge trials conducted in malaria-naïve individuals have been predictive of field efficacy results to date. There has to date been no vaccine that has demonstrated reproducible protective efficacy in malaria-endemic settings without first showing efficacy against controlled human malaria infection.[4]

Data from CHMI studies is therefore useful to groups funding large malaria vaccine trials in their decision as to which vaccine candidate will be prioritized for testing in endemic areas. It is generally accepted that prevention of infection in a proportion of malaria-naïve persons (as with the RTS,S/AS01 vaccine) represents evidence of partial efficacy, and reason for advancement of vaccine candidates to advanced clinical development pathways. Because of the critical importance of CHMI in assessing candidate malaria vaccines, there have been recent efforts to try to further standardize the model across institutions. The World Health Organization, in collaboration with the multiple centers currently conducting CHMI studies, recently produced a consensus document for this purpose.[5]

CHMI is regarded as safe and well tolerated. Common symptoms reported in participants in malaria challenge trials include fatigue, fever, headache, malaise, chills, myalgia, nausea and dizziness.[6,7] During the modern era of CHMI, which began in 1986, using mosquitoes fed on cultures containing Pf-infected erythrocytes more than 1800 volunteers have been challenged, and there have been no deaths.

Malaria therapy and drug efficacy studies from the 1940s through the 1970s provided detailed observations on the course of *P. falciparum* malaria in volunteers. In these studies, volunteers were allowed to remain parasitemic for several days prior to the initiation of effective therapy.[8-18]. Mean parasitemias were approximately 1000-fold greater than in current studies, reaching 88,713 parasites/mL as compared to 46.9 parasites/mL in more recent studies.[6]

The clinical methods used to assess malaria vaccine efficacy in humans have been established through trials evaluating sporozoite-based, or *pre-erythrocytic stage*, malaria vaccines.[19-22] During the *pre-patent period* (time before parasites are detectable in peripheral blood smears), volunteers are monitored closely for parasitemia and clinical sequelae. At *patency* (detectable parasites in

peripheral blood smears), volunteers are treated promptly with a curative course of an oral anti-malarial drug. Immunized volunteers not developing parasitemia (that is not developing patent malarial infection) are considered protected provided the non-immunized volunteers (controls), bitten at the same time and with the same group of infective-mosquitoes, experience a 100% attack rate.

Sterile immunity to *Plasmodium spp.* has been achieved in mice, monkeys and humans after irradiated sporozoite injection.[23-27] Irradiated sporozoite development is arrested at the level of the hepatocyte, abrogating processing to the erythrocytic stage of parasite development. Evidence points to hepatic stage, MHC-restricted $CD8^+$ T-cell-mediated responses as being crucial in mediating immunity to malaria, though $CD4^+$ T-cells, cytokines and antibodies may act in concert to prevent disease.[28,29] Repetitive CHMI will mimic acquisition of immunity through multiple controlled malaria exposures and this can be closely analyzed through advanced immunologic techniques.

The CHMI model effectively predicted clinical efficacy for the RTS,S malaria vaccine prior to clinical field trials.[30] As new candidate malaria vaccines reach clinical trial, the practice of these methods allows the protective efficacy of different vaccines and vaccine trials to be directly compared.

2.1.2 University of Maryland Malaria CHMI Experience

The University of Maryland Center for Vaccine Development (CVD) has extensive human clinical trials experience with malaria challenge studies from the early 1970s through to the present, and the evolution of the malaria vaccine testing program and CHMI are intertwined at the institution.[23,25,27,31,32] Formerly known as the International Health Program at the University of Maryland School of Medicine (UMSOM), a malaria research program was initiated in the late 1960s which included developing experimental malaria challenge models for *P. falciparum* and *P. vivax*; investigations into the biologic, immunologic, and drug response characteristics of strains of *P. falciparum* and *P. vivax*; and evaluating the potential for malaria transmission by several species of domestic and foreign Anopheles mosquitoes. Within a short period of time, the UMB malaria challenge model in volunteers became one of the most active programs of its kind in the country. In the early 1970s the Maryland program was expanded to include a trial of immunization against human malaria. The embryonic unit of what was to become the CVD was established at UMB to explore the possibility of carrying out volunteer studies using community volunteers. A notable achievement to emerge from this period was the demonstration that immunization of volunteers by the bite of hundreds of mosquitoes with irradiation-attenuated sporozoites elicited measurable rises of anti-sporozoite antibody and conferred protection for several months against challenge with sporozoites.[23,25,33] These research studies provided a critical, irrefutable demonstration that anti-sporozoite immunity in humans was protective and that a practical anti-sporozoite vaccine may be feasible.

The malaria challenge suites and insectary were updated and CHMI studies resumed in 2008, under the direction of the co-PI of this study. ***Since 2009, the CVD has conducted nine separate malaria challenge events using mosquitoes/sporozoites to deliver PfSPZ to >200 participants. No non-governmental research program in the U.S. approaches this experience.*** A study entitled, "An

Interventional *Plasmodium falciparum* Malaria Challenge Model Utilizing the NF54 Strain of Parasite Transmitted by Aseptic *A. stephensi* Mosquitoes to Healthy Malaria-Naïve Adult Volunteers (DMID 05-0053)", and a study evaluating parenteral ID injection of PfSPZ in 30 volunteers (DMID 11-0027) were both funded by the NIH Vaccine Testing and Evaluation Units (VTEU) and completed at the CVD. UMB has, in collaboration with the Naval Medical Research Center (NMRC) and Sanaria, Inc., conducted the first in human PfSPZ vaccine study with funding from PATH-MVI: "Phase 1/2A trial of the PfSPZ vaccine administered subcutaneously (SC) or intradermally (ID) to malaria-naïve adult volunteers".[34] Moreover, two trials were conducted with the Vaccine Research Center of NIH with one trial conducted at the VRC,[35] and another ongoing at UMB (VRC 314) involving multiple CHMI. A final study is currently underway assessing heterologous CHMI with the 7G8 strain of PfSPZ to assess the PfSPZ Vaccine in collaboration with NMRC and Sanaria, Inc.

2.1.3 Safety of the Controlled Human Malaria Infection by the Bite of Mosquito

A select number of institutions employ the use of the CHMI technique as a tool to evaluate antimalarial drugs and vaccines efficacy.[6,30,36,37] Presently, the centers with active challenge programs include The University of Maryland's CVD, the Walter Reed Army Institute of Research (WRAIR), Naval Medical Research Center (NMRC), Oxford University, Radboud University Nijmegen Medical Centre (RUNMC) and Seattle Biomedical Research Institute (SBRI). The Johns Hopkins School of Medicine (JHU) has conducted malaria challenge trials in past years. WRAIR has successfully used a similar CHMI model in over 1000 volunteers; experiencing no deaths, episodes of severe malaria, anaphylactic emergencies, serious or unexpected complications or recrudescent malaria infections in any of these individuals (Kent Kester, personal communication). The NMRC and CVD have a robust experience with CHMI studies and an equivalent safety record.[32,38]

In the cumulative published experience with which we are familiar, involving ~2,000 challenged volunteers, no deaths have been reported. The collective experience has clearly established that the *in vivo* malaria challenge model can be safely employed to test the efficacy of drugs, subunit vaccines and whole parasite vaccines. Risks associated with malaria challenge include local inflammatory reactions, pruritus, larger local reactions involving the whole forearm, allergic reactions to mosquito bites, and the development of malaria infection. Additional risks include possible side effects of the anti-malarial medication taken (particularly chloroquine (CQ)) following challenge with CQ-sensitive *P. falciparum*. CQ side effects include nausea, vomiting, diarrhea, abdominal pain, dizziness, headaches, sleep disturbances, blurred vision, pruritus, skin rash, exacerbation of psoriasis or porphyria, tinnitus, and photosensitivity. Coartem (artemether/lumefantrine) side effects are mild in most cases and include mild headache, dizziness and anorexia, tinnitus, tremor, palpitation, as well as unspecific reactions like arthralgia, myalgia, vertigo, gastrointestinal disorders, itch, sleep disruption and nasopharyngitis. Other fairly common side effects (more than 3% of patients) include sleep disorder, tinnitus, tremor, palpitation, as well as unspecific reactions like vertigo, gastrointestinal disorders, itch and nasopharyngitis. Atovaquone/proguanil (Malarone®) side effects include nausea, vomiting, abdominal pain, anorexia, diarrhea, headache, cough, dizziness, and rarely, anemia, oral ulcerations, insomnia, fever, edema, rash and alopecia. The study team extensively discusses these medications and their

possible side effects in detail both as part of the informed consent process and before initiation of treatment for participants who become infected after malaria-infection challenge.

Over the past 30 years, CHMI with sporozoites delivered via the bite of infected mosquitoes has been well tolerated. However, a number of SAEs have been documented with a trend in cardiac events (Table 1). An RUNMC male participant, who remained malaria-free as evidenced by sequential negative PCR tests, had a myocardial infarction 2 days after treatment with CQ; in retrospect, this participant appeared to have had coronary risk factors.[31,39] In addition, a second cardiac event was reported in a female participant at RUNMC.[40] This 20-year-old participant received three doses of a recombinant malaria protein vaccine containing aluminum hydroxide. The volunteer became parasitemic on Day 11 after challenge. Two days after completing a course of treatment with Riamet® (artemether/lumefantrine) for malaria infection, the participant was hospitalized with acute chest pain (Day 16 post-challenge). A diagnosis of acute coronary syndrome with limited myocardial necrosis of the inferior wall was made based on the reported pain, electrocardiogram (ECG) findings of <1mm ST segment elevation inferiorly and cardiac enzyme profile. A cardiac MRI was negative for evidence of atherosclerotic disease. The etiology of the event remains unclear. Subsequent evaluation revealed low hemoglobin on the day of challenge (6.9 mmol/L (normal range 8-12 mmol/L), and an abnormally elevated lumefantrine metabolite (desbutyl-lumefantrine) levels. Cholesterol, coagulopathy and autoantibody studies were normal or negative. Most recently (December 2014), a 23 year old male participant at RUNMC was diagnosed with asymptomatic myocarditis in the context of exposure to PfSPZ Challenge under cover of anti-malarial medication (taken with CQ); this diagnosis was via serial troponin measurements. Considering the sub-patent level of parasitemia during pretreatment with CQ, it was considered unlikely that parasitemia-induced microangiopathy was the underlying cause of the mild myocarditis.

Table 1

Table 1: Known number of volunteers sustaining a Serious Adverse Event after undergoing controlled human malaria infection

Institution	Year	Volunteer Age (yr)	Study Product	Anti-malarial	Serious Adverse Event
RUNMC	1999-2003	39	None	Terminally treated - CQ	The volunteer did not develop malaria. Received terminal CQ. Sustained inf-lateral MI post-treat Day 1
	2009	20	PfLSA-3	Riamet®	Chest pain D16 post-CHMI, troponin 11 µg/L, MRI 1 (-), MRI 2 (myocarditis?). Dx- Acute Cardiac Syndrome
	2013	23	PfSPZ-CVac ID	Day 2 of Malarone®	Chest/arm pain D14 post-CHMI, MRI – abnl, troponin – 1,115 ng/L, myocarditis
	2014	23	PfSPZ-Cvac by mosquito	CQ	10 day post-mosquito exposure, asymptomatic troponin elevation 168 ng/L, MRI-myocarditis
NMRC					None
WRAIR					None
UMD-CVD	2012	44	PfSPZ-ID	Malarone®	Treated on Day 15-17 post-CHMI, asymptomatic qPCR (+) D28 (290 para/µL), D33 (31 para/µL), D35 PCR (-)

CQ = chloroquine, PfLSA-3 – Pf Liver stage antigen 3, PfSPZ – Pf Sporozoite, PfSPZ Cvac – Controlled Pf experimental infection under CQ prophylaxis, ID – intradermal, MI – myocardial infarction

No reports of cardiovascular pathology or events have been reported at other sites with CHMI capabilities or with natural malaria and autopsy studies which reveal sequestration of parasites in all organs but no evidence of myocardial damage (n = 99).[41,42] One episode of coronary spasm in a 17 year old male was reported by Novartis after treatment of falciparum malaria with Coartem® (artemether-lumefantrine). A cannabis positive urine test was reported concomitantly. There have been no other published cases with complications resulting in severe disability or death. Serial troponin levels performed at the request of the DMID at the CVD revealed no increase or abnormal values in U.S. malaria-naïve, challenged volunteers.[43]

The CVD has extensive human clinical trials experience with irradiated sporozoite immunization and CHMI from the late 1960s through to the present.[7,27,31,34,38,44] Moreover, investigators at the CVD are part of a consortium of international scientists working with the WHO to standardize CHMI methodologies and standard operating procedures.[4,5] The challenge methodology at UMB uses several procedures to diminish the effects of confounding clinical and laboratory variables, and strives to improve the safety of CHMI in volunteers. Only one SAE has been reported. A male volunteer, who had been treated with Malarone® for symptomatic Pf malaria, re-developed a low, detectable qPCR at Day 28 post-CHMI, remaining smear-negative and asymptomatic. This asymptomatic event was deemed an SAE (Table 1) by the funding sponsor, DMID, but refuted as such by an independent study SMC. The CVD team feels that this low-level detection likely represented transient gametocytemia and the experimental qPCR reverted spontaneously to negative by Day 35 post-challenge.

2.2 Rationale

2.2.1 Acquisition of Malaria Immunity

In endemic malaria areas, individuals can be infected with *P. falciparum* multiple times although they are more likely to have severe, life-threatening symptoms on their first exposure to malaria than subsequent infections.[45] This observation has led to the hypothesis that the severe response to

the first exposure is due to a strong inflammatory/Th1 response and that this is down modulated during subsequent infections.[46] However this has never been demonstrated directly, because the initial response is difficult to study in the field where parasite exposure cannot be controlled, and patients do not arrive at clinics until they are already symptomatic. For the same reasons, symptomless exposures are difficult to track in the field. Over time, susceptibility to infection is also reduced after multiple repetitive exposures,[47] and this protection can be passively transferred from adults to children by administering purified immunoglobulin (Ig),[48,49] but neither the specific protective Igs nor the target antigens have been identified. To rigorously address these questions, we propose using an experimental malaria challenge model.[43] We will monitor the parasitemia and the corresponding immune response from the initial sporozoite inoculation to the detection of parasites in the blood and compare the responses obtained after 4 sequential challenges. These results will be used to evaluate whether prior malaria exposure affects parasite development, peripheral blood mononuclear cell (PBMC) activation and antibody production during subsequent infections. Our hypothesis is that repeat parasite exposures will induce changes in the immune response leading first to a reduction in fever/symptoms following parasite exposure and then after 3-4 infectious challenges a reduction in parasite growth.

The use of a well-established malaria challenge model using *Plasmodium falciparum (Pf)*-infected mosquitoes to initiate an infection at a defined time with a known parasite isolate, NF54, will allow the first systematic assessment of the response to the initial infection and subsequent parasite challenges.[43] This model was selected because of its similarity to natural infection in that a person is infected through mosquito exposure and the infection progresses in the absence of antiparasitic agents until parasites are detected in the peripheral blood. Taking this approach avoids the potential immunomodulatory effects of anti-malarials on the development of the immune response and the parasite. Since 1985, mosquitoes infected in vitro with chloroquine/Malarone/Coartem-sensitive *Pf* parasite lines have been used to challenge over 1800 volunteers to test vaccine efficacy.[25,32]In these studies, volunteers were closely monitored following infectious bites and treated with anti-malarials as soon as parasites were observed in a thick blood smear; however, *Pf* challenge has not previously been repeated in the absence of prophylaxis to directly evaluate the effect of parasite exposure on the immune response.

After deposition in the skin by a mosquito bite, some parasites quickly enter circulation, migrate to the liver and begin to replicate, while the remaining parasites are cleared from the skin and can be transported to the draining lymph nodes where they stimulate an immune response.[50] Six days later, infected liver cells rupture, releasing parasites that invade red blood cells, thereby initiating the erythrocytic stage of the life cycle, which produces 16-32 new parasites every 48 hours. After the first infection, blood stage parasite levels increase rapidly and can usually be detected by Giemsa-stained smear within 9-12 days and are accompanied by an increase in oral temperature. In vaccine trials, a delay in or lack of the appearance of parasites is taken as a measure of relative immunity, and it is expected that the interval required to detect parasites will lengthen after repeated challenges with the same strain[32] Since the vaccine target is the parasite, fever has not been used as an outcome in these previous studies.

Field studies have reported an increase in the levels of *Pf*-specific antibodies and memory B-cells in the response to natural malaria exposure.[51-53] However, in contrast to most viral and bacterial infections, the response is short lived and decreases significantly in the absence of transmission

during the six month dry season.[53] After each subsequent malaria season, there is a small increase in the anti-malaria Ig titer that begins to plateau after age 18 when most individuals are protected against clinical episodes. Interestingly, individuals that have grown up in a malaria-endemic area have fewer circulating immature B-cells and naïve B- cells and an expanded population of activated and atypical memory B-cells relative to malaria naïve individuals.[51] The mechanisms underlying the slow onset of immunity and the shift in B-cell populations remain unknown, but could be related to over stimulation induced by repetitive parasite exposure. The role of these changes in the decrease in severe symptoms observed following repeated exposures to the parasite is also unknown. The balance between pro and anti-inflammatory cytokines have been suggested to play an important role in modulating the immune response against malaria, particularly IFNy, TNFα and IL-10.[45] An increase in CD4+ T-cells that secrete IFNy and IL-10, as opposed to FoxP3+ regulatory T-cells, has been associated with a reduction in severe disease in human and mice.[54,55] However, symptoms in mice are exacerbated if IL-10 is administered at the time of infection, suggesting it plays a later role modulating the initial pro-inflammatory response.[56]

One study used the *Pf* challenge model described above to evaluate cytokine levels in naïve volunteers starting 4 days after a single *Pf* challenge and found that volunteers responded with either an anti-inflammatory response characterized by an increase in TGFβ levels 12-24 hours before blood stage parasites were detectable by PCR or a pro-inflammatory response with IFNy, TNFα, and IL-10 levels rising 2-3 days after parasite detection.[57] The pro-inflammatory response was associated with a decrease in parasite growth rate at the cost of higher fevers. The reasons for these different initial responses remain unknown, as does the volunteers' response to uninfected mosquito bites and subsequent *Pf* exposures. Another study compared the transcriptomes of peripheral blood obtained from *Pf* challenge volunteers when parasites were detected by Giemsa-stained smear with the transcriptomes of symptomatic adult malaria patients living in malaria endemic areas.[58] A number of genes were up regulated in both groups, including those in the NFKβ pathway and the inflammatory cytokines TNFα, IFNy, and IL1β. In contrast, genes involved in IL-10 regulation and MAP kinase pathways, which could play a role in modulating the immune response, were differentially up regulated in the natural response. The lack of additional time points in these studies makes it difficult to know whether the differences observed were related to the repeated parasite exposures of adults in endemic areas or other unrecognized differences associated with natural infections. It is possible that repeated malaria exposure enhances the production of IFNy- and IL-10-secreting CD4+ cells that suppress the development of symptoms and perhaps the formation of memory B- and T-cells or long lived plasma cells.

The use of repeated malaria challenges will allow the analysis of the early response to sporozoite inoculation and liver stage development and the subsequent changes that take place as merozoites are released from the liver to begin the blood stage of the infection. As in the single malaria challenge model, which has been used for years to test vaccine efficacy, treatment will not be initiated until parasites are detected in peripheral blood smears. This protocol is an attempt to simulate a natural infection where treatment is not started until parasites are detected in the peripheral blood. In addition to analyzing the cellular and humoral immune response, circulating plasma cells will be collected to determine Ig repertoire by sequencing and molecularly characterizing the maturation of humoral immunity following repeated challenges. Recent advances in high-throughput sequencing technology have made this comprehensive Ig repertoire analysis possible. Complementary determining region 3 (CDR3) sequences from the Ig variable

region can then be used to track B-cell production and maturation, as well as identify specific Ig variable regions that can be generated recombinantly and tested directly for antigen recognition and further functional evaluation. Ig sequence maturation and expansion will also be compared with changes in clinical symptoms and cellular and humoral immune responses to develop an integrated picture of the development of the immune response over time. These assays allow direct analysis of the early stages of the infection, which cannot be done in the field due to the inability to control parasite exposure.

2.2.2 Transcription profiling and immunophenotyping

In the present protocol blood cells and parasites will be isolated on Study Days 1, 2 and every other day until 7 days after the malaria-associated PBMC collection after uninfected and Pf-infected mosquito challenges and cryopreserved for leukocyte immunophenotyping after the conclusion of the 5th CHMI.[34] The initial immunophenotyping will include markers for naïve and memory B-cells, NK cells, dendritic cells, monocytes and T-cells, including $T_{h1/2}$, T_{h17} and T_{reg} cells and the corresponding cytokines, IFNγ, TNFα, IL10 and TGFβ. The maturation of the immune response will be followed through defined sequential malaria exposures in the same individual. The leukocyte immunophenotyping data from repetitive *Pf* challenges will provide a time course for the immune response to malaria that can be analyzed for correlates with distinct life cycle stages and peripheral parasitemia as well as allow comparison with data from field samples. In the future, immunophenotyping following antigen stimulation will also be performed to inform immune response maturation. In addition, the transcriptional profile of the samples will be evaluated using the Illumina Human HT-12 Expression BeadChip to identify changes in RNA levels of cytokines, transcription factors, signaling molecules and developmental markers. The transcriptional profile data will be compared with the immunophenotyping and antigen stimulation results to better understand the signaling molecules involved in defining the response.

2.2.3 Humoral Immune Response

The antibodies generated in response to the initial parasite exposure, as well as subsequent exposures, will also be directly evaluated. From passive transfer studies, protective antibodies are known to form over time during natural exposure,[48,49,59] but the Ig repertoire has not been fully evaluated. For example, It is not known whether a repeat exposure primarily stimulates new naïve B-cells, which would result in Ig with novel V(D)J exon rearrangements, or whether memory cells are activated, which would result in an expansion of existing V(D)J exon rearrangements and the accumulation of point mutations. It is also not known whether the diversity of the Ig repertoire varies over time, either diversifying from a limited initial pool, indicating expansion in the number of malaria specific clones or starting with an expansive repertoire and becoming more focused over time, possibly indicating selection of memory cells expressing high affinity antibodies. The development of next generation sequencing techniques (Illumina or Roche, 454) now allows direct analysis of the entire IgG variable region repertoire,[60,61] which allows a comprehensive and systematic study of IgG sequence maturation. With this advance, the sequences of Ig heavy (IgH) and light chain (IgK/IgL) variable regions expressed in plasma cells that are produced in response to a malaria infection can be systematically determined and the development of molecular techniques for recombinant antibody production and B-cell cloning allow the functional

characterization of sequences of interest. Plasma cells will be used for sequence determination because they are only produced after direct response to antigen and circulate for a short time (6-9 days) in the peripheral blood after exposure. Therefore, harvesting plasma cells 6-9 days after the detection of parasites in Giemsa-stained blood smears enriches plasma cell clones expressing infection-specific Ig repertoire, excluding antibodies unrelated to the experimental infection.

The IgH and IgK/IgL variable regions will be sequenced from RNA isolated from plasma cells obtained after the first uninfected exposure and each subsequent infection. The sequence repertoire obtained with high throughput sequencing will be evaluated to determine V, D, J, exon usage, the sequence of complementary determining region 3 (CDR3), and the presence of non-germline nucleotides introduced during the maturation of the immune response. An increase in new CDR3 regions over time will suggest the activation of new naïve B-cells, since the sequence of the CDR3 region is determined by recombination during B-cell development. In contrast, expansion of Ig with CDR3 sequences observed in samples from previous Pf challenges will suggest the activation of memory cells and an accumulation of point mutations could indicate affinity maturation. This data will be compared with transcriptome and immunophenotyping to evaluate changes in cytokines and PBMC populations that precede changes in the Ig repertoire.

Changes in the repertoire will also be compared with the ability of the antibodies in the corresponding plasma samples to bind to the surface of the sporozoites, merozoites or infected liver or red blood cells by immunofluorescence. The antigens recognized at different time points could be further identified using protein microarray or immunoprecipitation.[62] Ig variable region sequences that are specific for the corresponding samples could be selected for production of recombinant antibodies and tested for the ability to recognize the parasite surface and block growth in *in vitro* culture.[63,64] Use of a well-characterized parasite strain with complete genome sequence and annotation will facilitate identification of immunoprecipitated proteins by mass-spectrometry. Due to the slow onset of relative protection observed in field studies, it is anticipated that it will take 3 or 4 exposures to generate high affinity Ig that bind to the surface of the iRBC or merozoite.

2.2.4 **Summary**

The malaria challenge model will be used to characterize the immune response generated against sequential malaria infections. The initial activation will be monitored during the first days following the first exposure and this data will be compared with the response generated against an uninfected mosquito bite, as well as after repetitive feeds. The results will indicate if a volunteer's response to the parasite changes over time--specifically, whether during repeat infections there is an increase in inhibitory signals that could contribute to the decrease in severe symptoms and the delayed development of a sustained antibody response observed during a natural infection. Antibody response will also be evaluated to determine the contribution of naïve and memory B-cells by tracking V, D, J exon usage and CDR3 sequence repertoire. This work will provide insights into the complex interaction between the parasite and host response that will be used to inform the development of intervention and control strategies.

2.3 Potential Risks and Benefits

2.3.1 Potential Risks

The risks of a malaria challenge study to U.S., malaria-naïve volunteers include the discomfort sustained by mosquito bite challenge, the discomfort associated with periodic blood draws including large volume leukapheresis and the risk of acquiring clinical falciparum malaria. Additional details that are presented within the informed consent to volunteers include the theoretical risk of allergy or anaphylaxis to the infected mosquitoes, injection site reactions, complications from drugs used to treat malaria, drug resistant malaria, confidentiality concerns and the risk to both the mother and fetus should a volunteer become pregnant. These are discussed in the order in which they are presented in the informed consent below.

2.3.1.1 Risk of the Malaria Challenge

The goal of this study to is repeatedly challenge volunteers with the NF54 strain of *P. falciparum* sporozoites administered by the bite of mosquitoes. This is the first of its kind repetitive CHMI study. However, a study performed at the Radboud University Medical Center in Njimegen (RUNMC) exposed volunteers to the bites of mosquitoes on a monthly basis while they concomitantly took antimalarial medication, and symptoms as well as rise in qPCR appeared to become blunted over time. Subclinical or mild malaria infections are expected. Vigilant analysis of the malaria smears (threshold of detection is approximately 2-50 parasites/mm^3) will detect the presence of malaria infection in many participants before significant symptoms develop in the volunteer. Ultrasensitive (us) qPCR results (threshold of detection is approximately 16 parasites/mL) will be compared to malaria smears in real time. Because therapy will be initiated with the detection of the first parasite by blood smear, dangerously high levels or prolonged duration of parasitemia will not occur. Mild malaria symptoms include malaise, chills/rigors, nausea, vomiting, dizziness, arthralgia, abdominal pain, fever, myalgia, and headache (Section 8.1.2.4). The average time to finding parasites on a volunteer's blood smear in past studies was 10.5 days, with a range of 9-14 days. Only 50% of volunteers infected with malaria developed fever, and fever usually lasted less than 12-24 hours. Symptoms of headache, nausea, vomiting, and anorexia were well tolerated, lasting only an average of 2.5 days, with a range of 1 to 6 days. We anticipated that these risks would diminish with sequential CHMI. By CHMI 4, the symptoms associated with clinical malaria in the core group of volunteers had dissipated considerably, and the pre-patent period had become significantly prolonged (range 12-17 days). In anticipation of continued dimunition of symptoms and prolonged pre-patent periods (possibly no pre-patent period if protection against NF54 strained challenge occurs), the team will move towards outpatient daily follow-up after CHMI #5.

Severe malaria evidenced by neurological manifestations such as coma and/or seizure and obtundation, profound anemia due to hemolysis and bone marrow suppression, acidosis, respiratory distress, acute renal failure, splenic rupture and vascular collapse with shock and death is highly unlikely and has never been described in a CHMI. Manifestations of severe malaria are well described[65] and researchers at the University of Maryland have extensive expertise in the diagnosis and treatment of severe malaria. The pathogenesis of these conditions is multifactorial

but a major factor is sequestration of parasite-infected red blood cells, which is proportional to the degree of parasitemia.[65]

Researchers at the CVD have extensive experience with CHMI and the care of clinical malaria and have shown conclusively that although volunteers often become symptomatic with mild malaria, rapid diagnosis and treatment quickly attenuates the illness so that the challenge does not place the volunteer at undue risk.[31] The threshold of parasite detection on smear using the CVD methodology has been shown to be 2 parasites/µl which is the lower limit of human detection reported.[7] Furthermore, the University of Maryland Medical Center is located across the street from the Pharmaron facility and within blocks of the Lord Baltimore Hotel allowing for volunteers to be transported to this facility in the event that more intensive medical care is required. For CHMI #5, the core volunteers will be followed by us-qPCR as well as blood smear. The dimunition of symptoms allows for the conduct of the daily follow-up portion of the study to be moved to an outpatient status. The CVD has extensive experience in the conduct of CHMI as an outpatient having conducted six separate challenge events utilizing us-qPCR (VRC 314, Warfigher II, DMID 14-0040). Because infectivity controls are embedded within the challenge, and due to the fact that they have no underlying immunity to malaria, the controls will be followed with us-qPCR and treated based upon two sequential positive positive tests (to mitigate the low possibility of laboratory cross-contamination and a false positive PCR). However, blood smear detection is also available and can be performed for any reason. This is standard procedure for prior trials utilizing us-qPCR as the primary diagnostic assay. The core volunteers will continue to be treated based upon smear positivity in order to provide a basis of comparison to CHMIs #1-4, but we do not perceive this to be an increased risk due to the dimunition of malaria symptoms with acquisition of immunity.

Cardiovascular events have been described at a single site (RUNMC) and are described in depth (**Section 2.1.3**). A review of safety and clinical outcome of 47 volunteers who underwent experimental challenge with NF54 strain at NMRC, revealed that one subject experienced pleuritic chest pain lasting 2 days (onset of symptoms occurred one day before onset of parasitemia), and another subject reported shortness of breath lasting 1 day (onset 2 days after parasitemia and initiation of chloroquine). These 2 events were reported as unsolicited symptoms, and no further clinical laboratory information was obtained.[6] Troponin and serial ECGs were followed surrounding a recent challenge study (DMID 05-0053). No elevation in troponin level or abnormality in ECGs were noted in the 38 volunteers who underwent challenge (unpublished data).

2.3.1.2 **Drug Resistant Malaria**

The strain of *P. falciparum* malaria to be used in this study is pan-sensitive to antimalarials including CQ so there is no risk for drug resistance within the parasite. This means that the drugs used to treat malaria in this study, Malarone® (Atovaquone/proguanil) and Coartem® (artemether/lumefantrine), will rapidly and effectively remove all parasites and cure malaria. Coartem® and Malarone® are very well tolerated pharmaceutical agents that are second-line agents for chloroquine sensitive parasites. Both have advantages and disadvantages with Malarone® demonstrating a prolonged half-life and Coartem® requiring a more complicated

dosing regimen. Investigators will weigh the risks and benefits for each CHMI and the interval between the next CHMI to determine the optimal drug for use in volunteers.

2.3.1.3 Complications from Drugs Used to Treat Malaria

Side effects to antimalarial medication are a potential complication to participants engaged in this study. At the doses of drug used to treat experimental malaria, toxic manifestations of Coartem® (artemether/lumefantrine) are rare. Mild side effects including mild headache, dizziness and anorexia, tinnitus, tremor, palpitation, as well as unspecific reactions like arthralgia, myalgia, vertigo, gastrointestinal disorders, itch, sleep disruption and nasopharyngitis. Atovaquone/proguanil (Malarone®) will be used in the event of intolerance or allergy to artemether/lumefantrine (Coartem®) and vice versa. Minor side effects of atovaquone/proguanil (Malarone®) include abdominal pain, nausea, vomiting, headache, diarrhea and dizziness, anorexia, cough and rarely, anemia, oral ulcerations, insomnia, fever, edema, rash and alopecia. Acetaminophen and ibuprofen will be offered for minor symptom relief. Side effects of acetaminophen and ibuprofen include mild hepatotoxicity, nausea, abdominal pain, and dizziness.

2.3.1.4 Risks of Leukapheresis

The three major risks of leukapheresis using peripheral venous access are, in order of frequency:

1. Citrate reactions consisting of tingling or numbness (paresthesias) around the mouth and in the fingers and toes; nausea, chills, and possible vomiting; and muscle cramping. Mild citrate symptoms are very common and are expected to occur in up to one-third of leukapheresis procedures. These symptoms will be mitigated by asking volunteers to take Tums® tablets (oral calcium carbonate supplementation) prior to study procedure. Leukapheresis operators will be trained to ask donors every 10-15 minutes if they are experiencing any facial or digital tingling, and to promptly lower the blood flow rate, or temporarily halt the procedure until the symptoms completely resolve. Calcium carbonate (liquid or IV) as well as intravenous potassium and/or magnesium may be administered and the procedure restarted at a slower anticoagulant infusion rate after the symptoms resolve. Moderate to severe citrate toxicity should be very rare if attention is paid to eliciting milder symptoms and treating them promptly. Severe citrate toxicity will be treated by immediately stopping the procedure and administering calcium gluconate by slow intravenous push, with titration to donor symptoms, under the supervision of the medical staff of the Department of Transfusion Medicine.

2. Vasovagal reactions with lightheadedness and dizziness, more rarely fainting, and very rarely, progression to seizures. These reactions may occur in 2-4% of procedures, and will be prevented by giving all donors a 16-oz water beverage immediately prior to donation. If they occur, vasovagal reactions will be treated by postural manipulation and oral or intravenous fluid administration.

3. Hematoma and bruising at the needle insertion sites, and possible loss of less than 1 pint of blood due to needle infiltration and inability to return the blood in the machine. Large hematomas involving greater than 10 mL of blood occur in 1-3% of leukapheresis procedures and are directly related to the skill and experience of the leukapheresis operator and the adequacy of the donor's veins. Careful pre-donation venous assessment by the leukapheresis operator will ensure that only donors with adequate peripheral

venous access are accepted into this protocol. Inability to return the blood from the device to the person happens in less than 1% of procedures, and impact on the donor is minimized by ensuring a pre-donation hemoglobin of >9 g/dL. If the blood in the machine cannot be returned, at least 56 days must elapse before a subsequent leukapheresis procedure can be performed.

2.3.1.5 **Pregnancy**

Because of the known adverse effects that malaria has on a pregnant woman and her unborn child (such as spontaneous abortion, low birth weight and premature births), significant effort will be made to screen or detect pregnancy at multiple stages of the study. Women will have their serum pregnancy test run at the time of screening, and a urine pregnancy test prior to each CHMI, the day of the first malaria positive smear, and on Study Day 29. Additionally, all women who do not have written medical proof of sterilization or amenorrhea (defined as $\geq$ 1 year in duration with medical evaluation) will be asked to practice adequate contraception. Both Malarone and Coartem are categorized as Class C for use in pregnancy, which is to say that the benefits must outweigh the risks to the fetus. Some data suggests that Malarone may be safe for the fetus in the first trimester. Coartem has been associated with birth defects.

2.3.1.6 **Allergic Reactions and Anaphylaxis**

Theoretical risks that volunteers will be warned of include the risk of allergy and anaphylaxis to the mosquito bites themselves. The presence of salivary gland proteins has been known to stimulate histamine release and volunteers will be queried as to allergic reactions associated with mosquito bites.

2.3.1.7 **Blood Drawing**

Periodic blood draws will be necessary to closely monitor the volunteers and to perform malaria smears and PCR analysis for early detection of *P. falciparum* parasitemia. Universal precautions will be maintained for the protection of the volunteer and the study personnel. Alcohol swabs will be used to minimize the potential of a cutaneous bacterial infection being introduced by needle sticks. Throughout this study, the amount of blood collected will be much less than 35 tablespoons (525 mL) in any 8-week period (which is the amount of blood allowed to be drawn under the American Association of Blood Banks standards). One unique aspect of potential needle sticks to study personnel is the requirement for malaria prophylaxis in the event that this event occurs. Study personnel will be issued Atovaquone/proguanil (Malarone®) prophylaxis and will be referred to the University of Maryland travel clinic for outpatient follow-up in the event of an inadvertent needle stick.

2.3.1.8 **Confidentiality**

Volunteers will be alerted to the fact that confidential personal information is provided as part of the participation and that it may be disclosed to the IRB at the University of Maryland or Uniformed Services University of the Health Sciences. There is a theoretical risk that an

unauthorized person could achieve access to study records. All study records will remain locked in a secure office/facility, and data will be password protected.

2.3.2 **Known Potential Benefits**

There are no known health benefits to be gained by the volunteer participating in this study. Free medical treatment related to the CHMI will be provided to enrolled participants during the active malaria challenge phase and the surveillance period. Medical issues not related to the study will be referred to the volunteer's Primary Care Provider.

3 OBJECTIVES

3.1 Study Objectives

3.1.1 Primary Objective:

Determine whether protective immunity against parasite infection develops following repeat CHMI

3.1.2 Secondary Objectives:

1. Determine whether clinical signs and symptoms of malaria decrease in intensity and duration following repeat CHMI.

2. Track the production of antibodies that react with *P. falciparum* sporozoites and blood stage parasites.

3.1.3 Exploratory Objectives:

1. Identify which PBMCs are activated and proliferate on Study days 6 and 8, treatment and 7 days post-treatment initiation or, if aparasitemic, Study Days 13 and 21 to compare responses over time and to sequential CHMI.

2. Determine the RNA expression profile of blood cells collected on Study Days 1, 2 and every other day until 7 days after the inpatient PBMC collection or Study Day 21 if aparasitemic to compare responses over time and to sequential CHMI.

3. Determine the plasmablast immunoglobulin gene repertoire 21 days after the uninfectious challenge and 7 days after the malaria-associated PBMC collection or on Study Day 21 if still aparasitemic to compare responses over time and to sequential CHMI.

3.2 Study Outcome Measures

3.2.1 Primary Outcome Measures

1a. Occurrence of a positive malaria smear during the surveillance period after infected CHMI (+ 8-18, 20, and 28 days after challenge or until malaria detection (corresponding to Study Days 9-19, 21, and 29). For infectivity controls enrolled into infective CHMI #5, malaria

detection will rely upon us-qPCR to ensure safety while conducting this portion of the study as an outpatient.

1b. Occurrence and quantification of parasites using real-time quantitative PCR during the surveillance period after infected CHMI (+ 5-18, 20, 28 days after challenge or until malaria detection (Study Days 6-19, 21, 29).

3.2.2 Secondary Outcome Measures

1a. Occurrence of fever (>38ºC) attributable to malaria during the surveillance period (Study Days 6-29 (±2 days) post-CHMI).

1b. Occurrence of signs and symptoms attributable to malaria (malaise, chills/rigors, nausea, vomiting, dizziness, arthralgia, abdominal pain, myalgia, and headache; see section 8.1.2.4) during the surveillance period (Study Days 6-29).

2. Antibody reactivity against Pf pre-erythrocytic antigen, CSP, and Pf liver- and erythrocytic-stage antigen, GLURP, measured using plasma obtained during the surveillance period Study Day 6, Study Day 8, treatment day or Study Day 13 if aparasitemic, and 7 days after treatment initiation or Study Day 21 if aparasitemic.

3.2.3 Exploratory Outcome Measures

1. Percentage of naïve and activated effector and memory B and T-cell, NK cell, monocyte and dendritic cell populations during the surveillance period (Study Day 6, Study Day 8, treatment day or Study Day 13 if aparasitemic, and 7 days after treatment initiation or Study Day 21 if aparasitemic).

2. Gene expression profiles as measured in fluorescent intensity units using an Illumina Human HT-12 Expression BeadChip during the surveillance period (Study Days 1,2, 4, 6-19, 21 (+3), and 29 (±2)).

3. Plasmablast immunoglobulin gene repertoire profile as measured in log counts per million mapped reads per antibody species 21(+4) days after the uninfectious challenge and 7(+3) days after treatment or on Day 21 (+3) if aparasitemic on D13 after CHMI #1-5.

4 STUDY DESIGN

A prospective Phase 1 cohort study, assessing the initial induction and subsequent maturation of the immune response during sequential parasite exposures (x2-5)

Healthy, malaria-naïve, U.S. volunteers ages 18-50 years, inclusive

- Single clinical center
- The test agent will be non-aseptic *Anopheles stephensi* mosquitoes infected with *P. falciparum* NF54 strain sporozoites by membrane feeding upon erythrocytes infected with *P. falciparum* gametocytes.
- The vector will be laboratory-raised, female, *A. stephensi* mosquitoes
- An initial uninfected Pf malaria challenge will utilize mosquitoes not infected with Pf sporozoites.
- The initial infective malaria challenges (#1) will involve the 10 core volunteers (naïve to Pf exposure). Each subsequent CHMI cohort (#2-5) will consist of two groups comprised of the core group of primary study participants (n = 10) and infectivity controls (n = 6) per challenge. In the event of primary study cohort dropouts, replacement volunteers can be recruited if ≥2 CHMI remain in the study. These volunteers will not undergo mock challenge.
- Screening will be done within 56 days prior to the uninfected mosquito challenge.
- Screening safety labs will be considered valid if obtained within 56 days of the *Pf* NF54 malaria challenge and will be repeated if the window elapses. A urine pregnancy test will be performed prior to each controlled human malaria infection, the day of first positive malaria smear, and 28 days following challenge (Study Day 29).
- Route of malaria challenge will be Anopheles bites to the forearm of volunteers allowing the mosquitoes to completely engorge
- Entomologists and research staff will be present to ensure that each volunteer receives 5 infectious mosquito bites. Mosquitoes will be removed for salivary gland dissection after a 5 minute interval. An infectious bite is defined as a single blood meal, or observation of red blood cells in the midgut, accompanied by the presence of parasites within the salivary glands of the individual Anopheles mosquitoes. A blood meal by a mosquito that lacks *P. falciparum* parasites within the salivary gland will not be quantified as an infectious bite and a replacement mosquito will be added to the challenge (until 5 infectious bites are administered). For the uninfected mosquito challenge, a bite will be defined as a single blood meal (without presence of parasites).
- Study duration will be approximately 2-3 years of active follow-up per core participant (induction CHMI followed by infective CHMI x 5) and approximately 56 days for infectivity controls (newly recruited for each infective CHMI). Note: Replacement core participants will be followed for less than 3 years dependent upon the timing of recruitment.

- The initial infectious CHMI will be 2 months after the start of the uninfected CHMI and the 2nd CHMI will be scheduled 6 months after the start of the 1st CHMI. The subsequent infectious CHMIs (3-4) will be scheduled at approximately 6-12 months intervals.
- Study surveillance will include days 1, 3, 5-18, 20 (+3), and 28 (±2) post-challenge (Study Days 2, 4, 6-19, 21 (+3) and 29 (±2)) and 28 (±3) days after therapy (can range from Study Day 37-56 depending on day of malaria diagnosis and is timed from the day of last treatment dose). qPCR detection will commence on +5 days post challenge (Study Day 6). Blood smears (concomitantly performed from same EDTA tube) to evaluate the development of a positive malaria smear will be performed days 8-18, 20 (+3), and 28 (±2) post-challenge (Study Days 9-19, 21 (+3), and 29 (±2)). "Ultra-sensitive real-time" quantitative PCR results will be compared to the current "gold standard" malaria blood smear-day surveillance data. For the purposes of CHMI #5, it will used as the primary diagnostic assay for the infectivity control group, allowing for earlier malaria treatment and enhancing safety as part of outpatient follow-up. Blood smears may be made if symptoms dictate but will not be used as the threshold for malaria treatment. The quantification and kinetics of the PCR will also be used to evaluate the growth of parasite liver and blood stages after repeat CHMI and the results will be compared between core volunteers and the infectivity controls. PCR will not be used as the primary mode of diagnosing blood-stage malaria infection for treatment of core volunteers during this study.
- Coartem® or Malarone® will be used to treat all blood-stage malaria infections and outpatient surveillance will continue until 4 weeks after completion of antimalarial therapy. The companion drug will serve as second line therapy in the event of intolerance or allergy to the first chosen agent. Agents will be chosen based upon the anticipated interval until the next CHMI with Malarone® anticipated to have a slightly longer half-life.
- Malarone® will be offered as first line therapy (with Coartem® as a second line agent) in the event of volunteer withdrawal from the study prior to onset of a clinical malaria episode. This volunteer will be strongly urged to consent to malaria therapy for safety reasons.
- Follow-up of adverse events (AEs) and serious adverse events (SAEs) until resolved or stable.
- Laboratory analysis (CBC (WBC, hemoglobin and platelets) and biochemistries (creatinine, AST, and ALT) will be performed on the day of challenge, the day of first positive blood smear or +20 days (+3) after challenge (Study Day 21 (+3)) if aparasitemic and 28 (±3) days after treatment.
- Analysis of cardiovascular risk and safety evaluation will be performed at screening in all volunteers. A 12-lead ECG will be performed in each volunteer. Subjects with abnormal cardiovascular symptoms or findings (Refer to **Section 5** for definitions) will be referred to a cardiologist for further evaluation. Both a study investigator and a study cardiologist will review study ECGs.

The primary analysis for each challenge cycle will be conducted for all primary data (i.e., safety and parasitologic endpoints) one-month post each malaria challenge (Study Day 29 (±2)).

- Data for continued safety, secondary and exploratory endpoints will be collected through the follow up visit that occurs 28 days after the end of treatment

- Safety oversight per DMID guidelines will be provided by a SMC and local independent safety monitor (ISM) from the University of Maryland Medical System who will provide local medical consultation for the study. (Refer to **Section 9.6.1**)

5 STUDY ENROLLMENT AND WITHDRAWAL

The study population will be enrolled from a CVD database of volunteers from the greater Baltimore-Washington metropolitan area and will include 28 U.S. malaria-naïve, volunteers aged 18-45 years. The sample size of 28 reflects the core group of volunteers undergoing repeated CHMI and a unique group of 6 infectivity controls for infective CHMI #2-5. The total number of 28 may increase dependent upon withdrawals within the core group. Replacement volunteers will be added to the core group if $\geq$ 2 infectious CHMI remain over the entire course of the study. The outcome measure is discrete; whether the volunteer becomes parasitemic or not. Enrollment will occur over a 1-2 month (~4-8 weeks) period and the target population will reflect the community at large at the study site. Information regarding study materials will be mailed to potential subjects who have previously participated in vaccine trials or expressed willingness to participate at the enrollment sites and have signed an authorization form that they are willing to be contacted for future studies. This has been done with Institutional Review Board (IRB) approval. Educational meetings will be offered to the public both within and outside the university system for the purposes of informing individuals of the study. Every effort will be made to achieve a balanced volunteer population while avoiding the enrollment of vulnerable populations. Careful screening will be conducted to avoid enrollment of individuals with substance abuse or psychiatric difficulties.

Due to the occurrence of cardiovascular events (described in **Section 2.1.3**), screening for cardiovascular disease risks and sub-clinical cardiovascular disease will be performed, similar to those instituted in challenge centers worldwide (WRAIR (reference to SAE included in informed consent, cardiovascular risk screening and baseline ECGs), NMRC (reference to SAE included in informed consent, cardiovascular risk screening and baseline ECGs), and Oxford, U.K., (ECG and cardiovascular disease risks screened in volunteers prior to enrollment). Participants will be assessed according to the non-invasive criteria for assessing cardiac risk provided by Gaziano in 2008.[66] Participants falling into either of the two "low risk" categories defined as $\leq$5% risk for fatal or non-fatal cardiovascular event within 5 years and >5% risk but $\leq$10% risk for fatal or non-fatal cardiovascular event within 5 years, are eligible for inclusion. It is generally accepted as reasonable to exclude from challenge participants falling into the three "moderate risk" or "high risk" categories. Importantly, no evidence of cardiovascular compromise has been established as a result of the malaria challenge to date. Volunteers will be closely examined and questioned at all follow-ups for presence of cardiovascular-related signs or symptoms. Signs or symptoms of an event suggestive of a cardiac etiology will prompt a cardiology work-up.

The Center for Vaccine Development and Global Health (CVD) will be the site for volunteer enrollment, malaria challenge events, and outpatient follow-up. The inpatient/hotel phase follow-up (CHMI #1-4) will be performed at one designated site per CHMI (either Pharmaron or the Lord Baltimore Hotel).The General Clinical Research Center (GCRC) will serve as a back-up facility for outpatient follow-up.

5.1 Subject Inclusion Criteria

Both repeat CHMI subjects and the infectivity controls meeting all of the following inclusion criteria will be eligible to participate in this study: Refer to **Appendix B** for reference ranges for normal laboratory values.

1. Male or non-pregnant/non-lactating female between the ages of 18 and 50 years, inclusive.
2. Able and willing to participate for the duration of the study.
3. Able to provide proof of identity to the satisfaction of the study clinician completing the enrollment process.
4. Able and willing to complete the informed consent process.
5. Willing to donate blood for sample storage to be used for future research (Note: refusal to allow future use is exclusionary).
6. Willing to refrain from blood donation to blood banks for 3 years following P. falciparum CHMI.
7. Agrees not to travel to a malaria endemic region during the entire course of study participation.
8. Physical examination and laboratory results without clinically significant findings and a body mass index (BMI) ≤35.

Laboratory Criteria within 56 days prior to enrollment:

9. Hemoglobin ≥11.2 g/dL for women; ≥12.6 g/dL for men.
10. Platelet count within institutional normal range
11. Alanine aminotransferase (ALT) ≤ upper limit of normal
12. Serum creatinine ≤ upper limit of normal.
13. Negative for HIV and Hepatitis B/C infection.

Laboratory Criterion documented any time prior to enrollment:

14. Negative sickle cell screening test.

Female-Specific Criteria:

15. Negative β-HCG pregnancy test (serum) on day of screening or urine pregnancy test at subsequent time points for women of childbearing potential.
16. Women of childbearing potential (exclusive of women in a same sex relationship) must agree to use effective means of birth control.*

    * (e.g. oral or implanted contraceptives, IUD, female condom, diaphragm with spermicide, cervical cap, abstinence, use of a condom by the sexual partner or sterile sexual partner). Women with a history of amenorrhea (> 1 year duration) or surgical or chemical sterilization (e.g. tubal ligation, hysterectomy, other) must provide written documentation of infertility from a health care provider).

5.2 Subject Exclusion Criteria

Subjects meeting any of the following exclusion criteria will be excluded from study participation.

1. Women who are breast-feeding or planning to become pregnant during the time interval needed to complete the study.
2. Receipt of a malaria vaccine in a prior clinical trial.
3. Any history of malaria infection.
4. Evidence of increased cardiovascular disease risk; defined as >10% five year risk by the non-laboratory method[66].
5. Current use of systemic immunosuppressant pharmacotherapy.
6. History of a splenectomy, sickle cell disease or sickle cell trait.
7. Known history of anaphylactic response to mosquito-bites; or known allergy to artemether lumefantrine or atovaquone or proguanil or other component of the product.
8. Participation in any study involving investigational vaccine or drug within 4 weeks prior to enrollment, or expects to receive vaccine or drug during the 2-month post-challenge period.
9. Use or planned use of any drug with anti-malarial activity that would coincide with challenge.
10. Anticipated use of medications known to cause drug reactions with atovaquone-proguanil (Malarone®) such as cimetidine, metoclopramide, antacids, and kaolin.
11. Plans to undergo surgery (elective or otherwise) between enrollment and 4 weeks (28 days) after any of the challenges.
12. Received a licensed vaccine within 1 month prior to enrollment in this study or expects to receive one during the 28 day post challenge period.
13. History of psychiatric disorders or behavioral tendencies (including active alcohol or drug abuse) that in the opinion of the investigator would make compliance with the protocol difficult*

    *Medical and psychiatric illness defined as personality disorders, anxiety disorders, or schizophrenia or social condition, occupational reason or other responsibility that, in the judgment of the investigator, is a contraindication to protocol participation or impairs a volunteer's ability to give informed consent or to comply with the protocol schedule.

5.2.1 Justification for Exclusion of Pregnant Women and Children

Malaria infection during pregnancy can have adverse effects on both mother and fetus, including maternal anemia, fetal loss, premature delivery, intrauterine growth retardation and delivery of low birth-weight infants. Additionally, the inherent risk of a malaria infection would exclude persons < 18 years of age. Women who are pregnant or plan to become pregnant during the study period and persons <18 years of age are excluded from the study.

5.3 Treatment Assignment Procedures

5.3.1 Randomization Procedures

Ten participants (with 2-4 alternates) will be recruited sequentially for participation into the core group for repeated exposure to Pf malaria by the bite of mosquito. Prior to each subsequent infective challenge (#2-5), 6 unique infectivity controls (with 2-4 alternates) will be enrolled for the purposes of undergoing a single CHMI. Volunteers who withdraw prior to their initial malaria challenge will be replaced by an alternate. Volunteers who withdraw after their first infected challenge can be replaced if_ 2 CHMI remain in the overall study (Replacements will have between 2-4 infected CHMI during the overall study). Screening records will be kept on subjects who have signed the consent form. Study numbers will be assigned to participants of each cohort in the order in which they provide written informed consent to be enrolled in the trial. Volunteers will not be randomized.

5.3.2 Masking Procedures

Volunteers will be assigned an anonymized study number. The nature of the challenge is such that the CHMI and participant identity will be clearly visible to both the study participant and the administrator; however the anonymized volunteer number will be unknown to study personnel involved in diagnostics and immunologic assessment.

5.3.3 Reasons for Withdrawal

The following criteria will be checked at each visit. If any become applicable during the study, the volunteer's continued participation in the study will be evaluated on a case-by-case basis.

- Use of any antibiotic or antimalarial drug not approved by the study personnel beginning 28 days prior to the malaria challenge and extending to Day 29 of study surveillance.
- Use of any investigational drug or investigational vaccine post-malaria challenge during the study surveillance (Day of challenge until +28 days after therapy).
- Evidence of cardiovascular-related symptoms to include chest pain or discomfort, shortness of breath, change in exercise tolerance, and palpitations, felt to be cardiac in nature without alternate etiology.
- Chronic administration (defined as more than 14 days) of any dose level of immunosuppressants or other immune-modifying drugs during the study period and chronic daily use of inhaled steroids. Intermittent use of inhaled (<800 µg/day of beclomethasone dipropionate or equivalent) and topical steroids are allowed.
- Administration of any blood products during study surveillance including 4 weeks after malaria treatment.
- Systemic hypersensitivity reaction following administration of the Anopheles challenge. Severe (i.e., Grade III) local reactions will be evaluated to determine whether or not further study participation is warranted and whether local or systemic therapy is necessary

- Per the discretion of the PI in the event that circumstances arise preventing the subject's continuation in the study, (i.e. pregnancy, some SAEs, noncompliance).

Subjects are free to withdraw from participating in the study at any time upon their request.

A study subject will be discontinued from active participation in the study (and followed for safety) if:

- Any clinical adverse event (AE), laboratory abnormality, intercurrent illness, or other medical condition or situation occurs such that continued participation in the study would not be in the best interest of the subject.
- Development of any exclusion criteria may be cause for discontinuation.
- Development of a study product related SAE.

For the Repeat CHMI cohort:

- Development of severe intolerance to 2 or more of the anti-malarial medications used to treat Pf NF54 strain in CHMI.

Subjects are free to withdraw from participating in the study at any time upon request. Participants who elect to withdraw from the study will not be subjected to additional research portions of the study (research blood draws, qPCR, etc.) although they will be strongly advised to continue with the safety portions (i.e., blood smears, safety laboratories) of the study.

5.3.4 **Handling of Withdrawals**

Every effort will be made to collect safety and laboratory data on any participant discontinued because of an AE or SAE by continuing the safety follow-up procedures. If voluntary withdrawal occurs, the subject will be asked to continue scheduled evaluations and be given appropriate care under medical supervision until the symptoms of any malaria episode or AE resolve or the subject's condition becomes stable. If possible, subjects who leave the study area will be contacted by clinical investigators to collect safety follow-up data. Volunteers will be asked to carry identifying cards stating that they have been exposed to infective malaria parasites and listing contact information for study personnel, and will be contacted by cell phone (or beeper provided if they do not possess this item). If it is felt that inclusion of the study volunteer's data for analysis is compromised, the study volunteer will be terminated from the study and study data will not be included in analysis. This does not preclude the ethical responsibility of the investigators to ensure the safety of the volunteer and offer curative therapy for a malaria episode and to follow the volunteer for cardiac or other manifestations of disease. If the volunteer has already experienced a malaria episode and received curative therapy, continued inclusion in the study will be considered. Participants can be replaced in the event that they choose to withdraw from the study after the malaria challenge. Replacements can be recruited in the event that 2 or more CHMI remain in the overall study. Data will be cohorted and analyzed based upon sequence of CHMI (e.g., first infected CHMI results are included with replacement first CHMI results). Study non-compliance will be defined as missing 2 sequential outpatient evaluations or more than 24 hours (2 consecutive malaria smears) of contiguous in-patient evaluation without notification or explanation to allow for verbal follow-up. All data generated before withdrawal will be included in final study analysis,

and volunteers will be followed for safety. Events that occur and are not subject to participant control (e.g., death in the family) will be addressed on a case-by-case basis.

5.3.5 Termination of Study

The study may be discontinued due to the developments related to illness or at the discretion of the U.S. Food and Drug Administration, study investigators at Uniformed Services University of the Health Sciences, or the University of Maryland, IRBs, the SMC, and/or DMID or due to natural disaster (refer to **Sections** 14.6).

6 STUDY INTERVENTION/INVESTIGATIONAL PRODUCT

6.1 Study Product Description

The investigational challenge product, non-aseptic mosquitoes, infected with the NF54 strain of *P. falciparum*, have been reared and infected by Sanaria Inc., Rockville, MD.

6.1.1 **Acquisition**

Infected non-aseptic mosquitoes will be delivered to the University of Maryland, Division of Malaria Research/Center for Vaccine Development on the day prior to CHMI. Unused product will be returned or destroyed per instructions from DMID. Disposition and accountability records will be maintained at the CVD and kept in the study file.

6.1.2 **Formulation, Packaging, and Labeling**

Anopheles stephensi mosquitoes to be utilized in the challenge study are infected with *P. falciparum* parasites of the NF54 strain utilizing methods developed by Sanaria, Inc. Briefly, eggs from *A. stephensi* mosquitoes are placed in media for nutrition and growth. The eggs pass through a larval and pupae stage before emerging as adults. Adult female Anopheles mosquitoes are given a blood meal including erythrocytes infected with *Pf* NF54. Colonies will be monitored and containers of mosquitoes will be sampled 1-3 days prior to the CHMI for the percentage of mosquitoes with *P. falciparum* sporozoites in their salivary glands, and the mean numbers of sporozoites per infected mosquito. Only containers with a mean threshold of 10,000 sporozoites/mosquito and 40% or more infected mosquitoes will be used.

6.1.3 **Product Storage and Stability**

The test product for this study will be non-aseptic *A. stephensi* female mosquitoes infected with *P. falciparum* parasites of the NF54 strain. Upon transfer to the University of Maryland, Division of Malaria Research/Center for Vaccine Development the containers of mosquitoes will be placed in the insectary utilizing incubators that will maintain appropriate environmental conditions. The security of the insectary is critical and every effort will be made to restrict access to the insectary and provide for a secure location.

The University of Maryland has a protected suite devoted to an insectary, containment area, laboratory, and microscopy rooms, as well as incubation facilities for proper maintenance of temperature and humidity. The temperature can be maintained (generally at 28°C with humidity maintained between 70-80%). A hygrothermograph record of the insectary environment can be maintained and checked daily. Inside the insectary, photoperiods are regulated by sunrise/sunset simulators. A two-door barrier system along with negative pressure blowers (Dyneform Model ICL-48 (Windsor, CT)) prevent the migration of infected mosquitoes from the high containment challenge room to the waiting antechamber as well as from the challenge room and the adjacent laboratory space. An additional barrier door prevents mosquito migration into the clean molecular laboratory space. Thus, a total of two blowers and four barrier doors exist between the challenge

facility and the outside corridor. Access to the facility will be restricted to study personnel and escorted volunteers.

6.2 Dosage, Preparation and Administration of Study Intervention/Investigational Product

Malaria challenge is conducted by strapping an inoculation containment cage (a cardboard pint container) of *P. falciparum*-infected mosquitoes to the forearm of volunteers (note: the volunteer will be allowed to choose the arm for CHMI) and allowing the mosquitoes to completely engorge.[27,32,44,67] Containers with mosquitoes meeting pre-defined criteria (> 40% female infectivity) will be transported from the Sanaria facilities in Rockville, MD. In the past, the mosquito gland score has been the method of estimating the amount of sporozoites present in paired salivary glands of the mosquito upon dissection. The gland scores have been as follows: 0 (no sporozoites), 1 (1-10), 2 (11-100), 3 (101-1000), 4 (> 1000).[68] In virtually all previous studies, mosquitoes with a gland score of ≥ 2 (≥ 11-100 sporozoites per paired gland) were considered infectious.[32] It is thought that regardless of the density of sporozoites in the salivary glands, only a limited number of sporozoites can be inoculated during a 5-10 minute period (approximately 5-300),[69] because of the physical characteristics of the mosquito salivary duct they must traverse. Thus, from a theoretical perspective, it has not been expected that there will be many more sporozoites inoculated, regardless of the numbers of sporozoites per mosquito.

Prior to human challenge, the quantity of mosquitoes required for the volunteer (5 mosquitoes) will be removed from the container and placed in a pint, cylindrical cardboard containers with a nylon screen on the top of the container. After the mosquitoes are allowed to engage in a blood meal and engorge (typically for 5 minutes), the blooded mosquitoes that participated in feeding will be removed and dissected to determine 1) whether a blood meal was taken and 2) whether the mosquito had *Pf* sporozoites in her salivary glands. The total challenge event is anticipated to last 10-20 minutes. If necessary, additional mosquitoes will be obtained from the incubating containers in the insectary for use in additional feeds until the requisite numbers of infected mosquitoes (n = 5) with adequate sporozoite quantities (≥2 sporozoite gland count) have fed upon the participant within that cohort. This may extend the time of challenge past the predicted 10-20 minute time frame.

6.2.1 Storage and Stability (Shelf-life)

The non-aseptic Pf sporozoite-infected Mosquitoes will be transported to the clinical trial site on the evening prior to CHMI and stored in humidity and temperature regulated incubators which will be monitored electronically. Receipt of Pf sporozoite-infected Challenge Mosquitoes will be documented on a Tracking Log by study staff.

6.3 Modification of Study Intervention/Investigational Product for a Participant

Not applicable

6.4 Accountability Procedures for the Study Intervention/Investigational Product(s)

Accountability logs will be maintained at the CVD to register product receipt and dispensation for accurate inventory tracking. Unused study product will either be destroyed on site or returned to Sanaria Inc. as per Sponsor instructions. The infected mosquitoes will be delivered to the UMD CVD by Sanaria in a container designed to transport the mosquitoes. Only the investigators and laboratory technicians along with the entomology team will have access to the insectary. The insectary is maintained in a locked facility with guards stationed at the entry portal to ensure that only proper personnel are allowed into the Center for Vaccine Development. After bite administration, the mosquitoes used in the challenge will be dissected and results recorded into a dissection log to be maintained by the CVD. The remaining mosquitoes that were not used in the study will be destroyed at the site after notifying the sponsor. Typically, mosquitoes are destroyed by freezing at -20°C for 24 hours followed by appropriate disposal into Biohazard waste.

6.5 Assessment of Subject Compliance with Study Intervention/Investigational Product

Not applicable

6.6 Concomitant Medications/Treatments

At each study visit/contact, the investigator will question the participant about any medication taken or applied as a topical since the prior visit, including holistic/naturopathic medications. Concomitant medication, including vaccines, and any other medication relevant to the protocol, including any specifically contraindicated (e.g., relevant antibiotics or antimalarials within 14 days, oral or parenteral steroids, high-dose steroids, cimetidine, metoclopramide, antacids, kaolin, or immunosuppressive or cytotoxic drugs, experimental vaccine or agent) or administered during the period starting from one week before (with the exception of antibiotics and antimalarials) each malaria challenge and ending ~two months (Day 28 post-therapy) after will be recorded in the CRF with trade name and/or generic name of the medication, medical indication, start and end dates of treatment. In the event that an antibiotic is determined to have been administered, the assessment of study exclusionary criteria will be handled on a case-by-case basis. Activity against *Plasmodium spp.* is not a universal attribute of all antibiotics and ongoing participation in the trial will be determined by investigators in consultation on the basis of the product administered. If a volunteer has initiated an antacid or kaolin, any administration of Malarone will be timed to separate administration of antimalarial medication by more than 4 hours to prevent absorption interference.

7 STUDY SCHEDULE

7.1 Screening

7.1.1 Repeat CHMI Subjects

Days -56 to -1: Recruitment will be progressive until 10 individuals who fulfill the inclusion criteria are included. Initial screening will occur over 1-2 days.

7.1.2 Infectivity Control Subjects

Days -56 to -1: Prior to the **2nd, 3rd, 4th and 5th CHMI,** an additional 6 subjects per challenge who fulfill the inclusion criteria will be recruited to serve as infectivity controls. Replacements for core group withdrawals will be permitted prior to the 2nd and 3rd CHMI.

Volunteers will be recruited among adults 18-45 years of age who have been previously enrolled in CVD studies and reside in the greater Baltimore/Washington metropolitan area. Additionally, educational seminars will be offered from which interested parties may request information on participation if desired. After the study has been explained to the potential volunteers, they may leave and return later with their decision allowing time for careful consideration of their involvement in the study. The informed consent process will be conducted in a private room at the Center for Vaccine Development (CVD) to ensure confidentiality.

After community information is disseminated as described in **Section 5**, all interested individuals will be invited to visit the study clinic on a specific date. These individuals will receive oral and written explanation of the study, after which consent will be obtained from those willing to participate. All screening tests, medical history and examinations will be performed only after an informed consent is obtained. A volunteer quiz consisting of ~10 questions will be administered to participants and infectivity controls. A passing score of 70% will be required prior to enrollment (See Appendix A). Volunteers who score less than 70% will be re-educated and will be allowed to retake the quiz for a second time.

Upon screening, the Investigator will prepare a screening form for each participant. This screening form will later become part of the CRF for participants enrolled in the challenge trial. A unique identification number will be assigned to each study participant. Participants will provide a medical history, with special attention to any history of recurrent infections to suggest immune suppression, previous history of splenectomy and prior allergic reactions. In addition, cardiovascular disease risk (defined as > 10%, 5 year risk) will be screened for utilizing a non-laboratory based method. Risk factors include sex, age (years), systolic blood pressure (mm Hg), smoking status (current vs. past or never), BMI (kg/mm^2), reported diabetes status, current treatment for raised blood pressure.[66] Participants will also undergo physical examination and laboratory screening tests, which include: CBC (Hemoglobin, WBC, Platelet count), AST, ALT, creatinine, hepatitis and HIV serologies, sickle cell trait, and serum pregnancy analysis (if appropriate). The standard for processing blood in commercial laboratory settings and at the CVD is to practice Universal Precautions, and they will be followed. Pre- and post-test counseling will

be provided prior to HIV testing in accordance with Maryland law and CVD SOP 302.03. All blood will be drawn by experienced CVD personnel and will be placed in vacuum-sealed blood tubes for transportation to Garcia Laboratories (or the University of Maryland Medical Center (UMMC) as back-up). Finally, a 12-lead ECG will be performed. An abnormal ECG may be defined as showing pathologic Q waves and significant ST-T wave changes; left ventricular hypertrophy; any non-sinus rhythm excluding isolated premature atrial contractions; right or left bundle branch block; or advanced (secondary or tertiary) A-V heart block. Cardiology back-up may be obtained if questions arise as to the suitability of a candidate based upon the ECG results. A participant who meets any of the exclusion criteria will be excluded. All screening tests will be completed within the 56 days prior to entry into the study but volunteers who are screened prior to Days -56 will have CBCs, biochemistries, and serum or urine pregnancy tests repeated within 56 days of the challenge event so as to maintain accurate safety lab information. Laboratory studies may be conducted at other times during the course of the trial if the investigators judge it necessary for the safety of the participant. All screening and follow-up diagnostic laboratory testing will be drawn at the CVD in Baltimore and sent to commercial testing facilities for analysis. ECGs will be performed and interpreted at the University of Maryland. Recruitment will continue until 10 eligible participants have fulfilled all of the inclusion criteria and none of the exclusion criteria, and signed the study consent form. Should participants change their minds and decline to participate prior to the uninfectious or first infectious challenge; additional participants will be screened as alternates and will be on hand for the day of challenge to ensure enough participants are challenged to meet the requirement of the study. Prior to each repeated CHMI (#1-5) baseline laboratories will be performed. An HIV ELISA will be repeated annually. Eligibility may be affected if the baseline laboratories or HIV findings fail to meet eligibility criteria. Transient lab abnormalities will not result in study disqualification if screening values met enrollment criteria.

7.2 Enrollment/Baseline

After meeting eligibility requirements, individual participants will be educated as to parameters required of undergoing a CHMI. The eligibility criteria will be reviewed on each individual at enrollment to ensure that each individual was properly entered into the study. Repeat pregnancy tests (urine) will be required of all female participants in the absence of written medical proof of sterilization or amenorrhea (defined as $\geq$ 1 year in duration with medical evaluation) on Study Day 1 (CHMI event). The ramifications of becoming pregnant during the study will be discussed in the informed consent process. Baseline, pre-CHMI complete blood counts, creatinine, AST and ALT will be drawn for each infectious CHMI (*Note: Safety labs are not required for the uninfectious CHMI*). Study samples for lymphocyte immunophenotyping studies will be drawn (See **Section 8.2.2**).

7.2.1 **CHMI**

Repeat CHMI Subjects Day 1: Enrolled volunteers in the core group (n = 10) will undergo one uninfected challenge (mock) characterized by exposure of volunteers to the bite of mosquitoes not infected with Pf (Appendix C Tables C1 - C3). CHMI #1-5 will be conducted by the bite of 5 mosquitoes infected with the NF54 strain of *P. falciparum* to the forearm of a volunteer. Replacement volunteers are permitted assuming $\geq$ 2 CHMI remain in the study. After malaria

challenge, the participant will be observed for local and systemic reactions for a minimum of 30 minutes.

Naive infectivity Controls (n = 6) will be enrolled for each of CHMI #2-5 (naïve US volunteers will not require infectivity controls for CHMI #1) (Appendix C Table C4).

All volunteers will be monitored for a minimum of 30 minutes for any evidence of acute reactivity to the mosquito challenge. Anaphylaxis to sporozoites or salivary gland material within mosquito bites is an exceedingly rare event. Nevertheless, access to ACLS equipment and medications such as epinephrine and anti-histamines are available on site.

7.3 Follow-up

Days 1 to 56: Signs and symptoms will be solicited through use of a memory aid (Days 1-5) recorded by the study personnel and/or investigators. Participants will undergo surveillance and follow-up combining outpatient and inpatient (CHMI #1-4) or daily outpatient follow-ups (CHMI #5) approaches to maximize safety, resource utilization and volunteer compliance.

7.3.1 CHMI and outpatient follow-up

Following inoculation of *P. falciparum* parasites via mosquito bite, volunteers will informed of the symptoms to monitor for malaria infection. An emergency notification card with study information and contact numbers will be issued for the volunteers to carry in their wallets or on their person. If they notice any symptoms or evidence of fever, they will be instructed to contact study personnel immediately and will have been provided landline, beeper and cell phone contact numbers. In the event that the volunteer does not have a cell phone, a Trak® phone will be issued for the duration of the post-CHMI period of the study to ensure a means of volunteer contact. In the absence of symptoms, repeat CHMI subjects will be instructed to return for outpatient clinical evaluations on Study Days 2, 4, and 6 -8 (Appendix C Tables C1 & C2). Memory aids will collect data on Days 1-5 and will be reviewed by study personnel on Study Days 2 and 4 and collected on Day 6. After challenge, we will collect blood at defined periods of time to assess the immune responses (i.e., Study Days 2, 4 and every other day until 7 days after treatment or until Study Day 21 (+3) if aparasitemic).

7.3.2 Inpatient follow-up

On Study Day 9 (evening), all volunteers will be admitted to the inpatient ward of Pharmaron or The Lord Baltimore Hotel (Appendix C, Table C3, CHMI #1-4). Volunteers will be admitted as a cohort to only one designated location per CHMI. They will have the opportunity to leave the ward/hotel and are encouraged to attend to daily activities if they are symptom-free but will be expected to remain at the ward nightly from approximately ~7 PM to 7 AM (investigator discretion can enable alternate check in times for individuals with valid conflicts). However, ward/hotel rules will be implemented for volunteer safety (refer to MOP). Daily malaria smears will continue until a positive smear is obtained, at which time the volunteer will undergo directly monitored treatment and female subjects will have a urine pregnancy test. Inpatient diagnostic examination includes

daily malaria smears and PCR analysis. While they are blood smear positive, we instruct volunteers to remain on the unit/floor while they receive supervised therapy for three days. If symptoms develop that are consistent with malaria (i.e., headache, fever, etc.) in blood-smear-negative individuals, blood smears can be performed q 6-12 hours in order to heighten surveillance for the development of malaria. If a positive blood smear is noted, a CBC, creatinine, AST, and ALT will be performed. A CBC will be repeated on apheresis day. A final CBC, creatinine, AST, and ALT will be performed 4 weeks after treatment as a final visit or D29 (±2) if they remain aparasitemic. Three sequential smears (> 12 hours apart) will be recorded to affirm clearance of malaria. The volunteer can be discharged after the documentation of two sequential negative blood smears. The third smear can be obtained after the volunteer is discharged from the inpatient unit/floor.

7.3.3 **Outpatient daily follow-up**

On Study Day 9, volunteers will commence daily follow-up (Appendix C, Table C3). As per **Section 7.3.2**, volunteers will receive daily us-PCR analysis. Core group members will continue to have concomitant daily malaria smears. This will also be available to the control group members if required. If two positive sequential us qPCRs (for infectivity controls and separated by < 60 hours) or a single positive blood smear is noted, a CBC, creatinine, AST, and ALT will be performed. A CBC will be repeated on apheresis day (core group). A final CBC, creatinine, AST, and ALT will be performed 4 weeks after treatment as a final visit or D29 (±2) if they remain aparasitemic. Documentation of reversion to a negative us qPCR is not performed as this can remain positive for many days and is not indicative of ongoing parasitemia. However, three sequential negative blood smears (> 12 hours apart) will be recorded for the core group volunteers. Daily follow-up will cease after the 3rd negative smear (core group) or after daily directly observed thereapy (infectivity controls).

7.3.4 **Outpatient follow-up**

At each outpatient visit, volunteers will be questioned for the presence of cardiovascular-related signs or symptoms (chest pain or discomfort, shortness of breath, change in exercise tolerance, and palpitations). Any volunteer with cardiovascular symptoms will be referred to cardiology for evaluation. If a volunteer ***has not developed a positive malaria smear*** by Study Day 19 and ***remains symptom free***, he/she will be discharged from the inpatient/hotel/daily outpatient follow-up setting and will continue to be followed (Study Days 21 (+3), 29 (±2) and 28 (±3) days after therapy) after which he/she will be followed per protocol for repeat CHMI. Coartem® or Malarone® will be offered as first line therapy in the event of volunteer withdrawal from the study prior to onset of a clinical malaria episode. It is highly unlikely that a volunteer would be able to transmit malaria via the bite of a mosquito if they remain aparasitemic and asymptomatic. Transmission of malaria requires gametocytemia. Since the advent of modern CHMI in 1986, during which volunteers have been managed according to the procedures to be followed in this clinical trial, there have been no reported cases of volunteers developing gametocytemia.[37] In the field gametocytemia was detectable in approximately 2.4% of smear positive cases of uncomplicated *P. falciparum* malaria[70] and rises with environmental stressors such as malaria therapy. While a single schizont develops into either a gametocyte or an asexual stage parasite, the

mechanism for this pre-determination is unclear. Evidence of gametocytemia in the setting of a treated, uncomplicated malaria infection in the field is well-described, but as stated above gametocytemia has not been observed in the setting of CHMI managed according to our protocol. ***For volunteers who have not developed clinical malaria,*** clinical evaluations, malaria smears and/or PCR (PCR alone for infectivity controls of CHMI #5) will be continued at each visit until volunteers develop symptoms, a positive malaria test, or Study Day 29 (±2) is reached. A targeted clinical exam will be performed and a urine pregnancy test will be performed in women of childbearing potential on Study Day 29. Blood samples for RNA isolation will be collected every other day until apheresis. For study volunteers who develop clinical malaria, samples will be collected every other day until 7 (±1) days after treatment. Day 28 (±3) post-therapy (Days 37-56) is the last scheduled active clinical follow-up visit for each individual CHMI; however the core group of volunteers will remain in study follow-up until completion of the remaining sequential CHMI.

Every effort will be made to ensure compliance with visits and provide for the safety of the participant. If a participant does not appear for a scheduled clinic visit, attempts will be made to contact the participant and arrange transport to the clinic center. If a serious adverse event (SAE) has occurred, appropriate measures will be taken to notify the CVD independent safety monitor, DMID, and IRBs as described in **Section 9.3.1.**

7.3.5 Malaria Infection

Following CHMI, all Repeat CHMI volunteers will be followed as an outpatient on Study Days 2, 4, 6, 7, and 8 (Appendix C Tables C1 & C2). All (repeat CHMI and infectivity control #1-4) volunteers will be admitted for nightly observation to Pharmaron or The Lord Baltimore Hotel from Study Days 9-19 (Appendix C Table C3). Typical patency extends from 8-16 days after challenge and recent studies conducted at the CVD documented time to infection, defined as a positive blood smear, as being from 9-14 days after challenge (mean 10.5). Due to the repetitive CHMI, acquisition of immunity has been noted among the core group volunteers with pre-patent periods extending from 12-17 days (mean 14.25 days) after challenge, accompanied by very few symptoms during CHMI #4. Among the more than 1800 CHMIs (among naïve volunteers) during the past 25 years there have been several instances of longer pre-patent periods, but they have been extremely rare. The longest recorded patency by blood smear documented in the literature was 21 days.[6] One volunteer was documented to have a self-limited, blood-smear negative, positive malaria culture 21 days and 30 days following challenge.[44] Volunteers were admitted and daily smears and qPCR assays performed for CHMI #1-4. CHMI #5 will move to follow-up on an outpatient basis due to the dimunition of symptoms among the core volunteers. Participants will continue to be queried daily as to the presence of malaria-specific signs and symptoms (Malaise, chills/rigors, nausea, vomiting, dizziness, arthralgia, abdominal pain, fever, myalgia, and headache). If symptoms develop that are consistent with malaria in blood-smear-negative individuals, blood smears or us qPCR can be performed q 6-12 hours in order to heighten surveillance for the development of malaria. Volunteers will be questioned at all follow-ups for the presence of cardiovascular-related or other related signs or symptoms (shortness of breath, chest pain, change in exercise tolerance, and palpitations). We do not expect complications or severe events to occur as a result of an experimental malaria challenge but any volunteers who display any symptoms that require more aggressive monitoring can be transferred within the

UMMC. Pharmaron is located directly across the street from UMMC, while The Lord Baltimore Hotel is located five blocks away with 24/7 shuttle service. We would base transfer decisions upon the clinical stability of the patient. Labile vital signs including accelerated respirations indicative of acidosis, hypotension, hyperthermia, evidence of organ compromise such as symptomatic hepatic compromise with LFT's greater than 5X ULN, renal insufficiency or parasitemia unresponsive to anti-malarial therapy are all hypothetical reasons for transfer. Complete blood counts, creatinine, AST and ALT will be drawn on the first day of positive malaria smear, and at outpatient visit 28 days post-therapy (in the event that the volunteer does not acquire malaria and remains aparasitemic, the laboratory values will be repeated on Study Day 29 (±2). qPCR analysis will continue for 2 days after the smear reverts to negative or for the period of directly observed therapy in the case of CHMI #5. Participants will receive Coartem® or Malarone® as first line therapy or terminal prophylaxis over 3 days. For infectivity controls enrolled into CHMI #5, dosing will occur following two sequentially positive (> 12 hours apart but < 60 hours) us qPCR values, in order to adhere to outpatient follow-up standards utilized in previous trials. The clinical and patency data for these infectivity controls will not be combined with data from CHMI #1-4. Coartem dosing is four 20mg/120mg tablets as a single initial dose, four 20mg/120mg tablets again after 8 hours and then four 20mg/120mg tablets twice daily (morning and evening) for the following two days. Atovaquone/proguanil (Malarone®) is dosed at four 250/100mg tablets orally for three days, representing a total daily treatment dose of 1 gram/400mg. In the event that the volunteer is taking antacids or kaolin, the dosing will be spaced apart from the medication by 4 hours. Three sequential smears will be recorded to document clearance of parasitemia for core group volunteers. Reversion to negative us qPCR is not required for infectivity controls. The volunteer will be permitted to leave after the second negative smear (CHMI #1-4), if the full malaria treatment dose has been administered, and return the following day as an outpatient for acquisition of the third blood smear. Ibuprofen and/or acetaminophen will be offered for symptomatic relief of fever or body aches.

The safety of our volunteers is paramount. In the report of Epstein et al.[6], 62% of volunteers had symptoms for 12 hours before documentation of a positive blood smear. Similarly, studies at the CVD have documented that approximately 55% experience mild symptoms (e.g., malaise) prior to documentation of a positive blood smear (unpublished data). We intend to increase blood smear/us-qPCR assessments to every 6-12 hours when there are symptoms consistent with malaria, while closely monitoring for alternate causes of these symptoms (i.e., influenza, gastrointestinal illness, etc.). In individuals with positive symptoms, we will also increase the number of passes along the 1 cm axis from 5 to 10 to increase the sensitivity by 2-fold. When reading 10 passes the microscopist will be assessing approximately 1.0 µl blood, which means the microscopist will be able to detect 1-2 parasites/µl blood. Based on a normal value of 4×10^6 erythrocytes/µl blood, this means we will be able to detect *Pf* parasites in the bloodstream when 0.000025% of erythrocytes are parasitized (0.000025% parasitemia). Since the definition of severe malaria based on parasitemia is at a minimum > 3% parasitemia and often 10% parasitemia, this means our microscopists will be able detect a level of parasitemia at least 5 orders of magnitude below the cut-off value for severe malaria. In the case of persistent symptoms and negative blood smears (with increased numbers of blood smears per day and examination of blood quantity), we will weigh the risk to the volunteer, the extent of symptoms, and clinical stability of the volunteers on an individual basis before instituting anti-malarial therapy. The study objective is to reliably transmit malaria to 100% of volunteers and assess the immune response over time. A false-positive

diagnosis is of critical concern in our ability to accurately interpret the results of the challenge study. Ultra-sensitive qPCR which can be expected in most cases to precede a positive blood smear by 3-5 days will be used as an additional tool to confirm the diagnosis if necessary. It will not be used a first line diagnostic in this study to better simulate natural exposure and diagnostic practices in the field. The us-qPCR results will serve as a harbinger of impending smear positivity for core group volunteers to alert study personnel and slide reading team members, and will serve as the diagnostic test for treatment for CHMI #5 infectivity controls. Additionally, if the subject remains aparasitemic until +20 (+3) days after challenge, the qPCR results will be examined to determine if patency is imminent and team members will determine whether to delay leukapheresis to maximize plasmablast collection (+7 post-the inpatient PBMC collection).

7.3.6 **CHMI Conclusion Visit 20 (+28 days post-therapy (+/- 3 days)**

The CHMI conclusion visit will occur on +28 days (±3) post-therapy. It is unlikely that *P. falciparum* malaria will occur after this late time point or recur after treatment, but participants will receive information to contact study coordinators and local emergency care facilities should symptoms consistent with malaria infection recur subsequent to repeat CHMI or study termination. Queries as to antibiotic usage will be performed to assure that the participants have not consumed antibiotics such as trimethoprim-sulfamethoxazole, azithromycin or doxycycline that could result in suppression of the erythrocytic manifestations of malaria. Information regarding AEs will be solicited at the final scheduled study visit. Any ongoing related AEs will be followed to resolution or until a stable chronic condition has been established. Volunteers will be encouraged to contact study personnel with symptoms consistent with malaria for the entire interval between repeat malaria challenges.

7.4 Final Study Visit

For infectivity controls, the final study visit will occur on +28 days (±3) post-therapy. For repeat CHMI volunteers, the final study visit will occur on +28 days (±3) post-therapy of CHMI #5 (or the final CHMI for replacement core volunteers). It is unlikely that *P. falciparum* malaria will occur after this late time point or recur after treatment, but participants will receive information to contact study coordinators and local emergency care facilities should symptoms consistent with malaria infection recur subsequent to repeat CHMI or study termination. Queries as to antibiotic usage will be performed to assure that the participants have not consumed antibiotics such as trimethoprim-sulfamethoxazole, azithromycin or doxycycline that could result in suppression of the erythrocytic manifestations of malaria. Information regarding AEs will be solicited at the final scheduled study visit. Any ongoing related AEs will be followed to resolution or until a stable chronic condition has been established. Volunteers will be encouraged to contact study personnel with symptoms consistent with malaria for the entire interval between repeat malaria challenges.

7.5 Early Termination Visit

Every attempt will be made to retain the participants once the trial has commenced. Early termination has special concerns given that the primary outcome of this study is to assess the maturation of the immune response following repeated CHMI. While early termination is

ordinarily quite safe and early parasitemias will be caught before significant symptoms are allowed to develop, unmonitored infection can progress to serious illness. Proper participant education at enrollment and stringent follow-up requirements will impress upon the volunteers the seriousness of compliance. As previously stated, two forms of contact will be established as well as the information for a designated point of contact recommended by the volunteer. Volunteers with any question of reliability will be asked to remain on the inpatient ward for the duration of the in-patient portion of the study, including completion of drug therapy. Participants will carry identifiers to inform outside medical personnel that they have been infected with malaria. Volunteers who insist upon terminating the study prematurely will be asked to submit a malaria smear and a PCR analysis. They will be offered therapy as prophylaxis against a malaria infection (atovaquone/proguanil (Malarone®) as four 250/100 mg tablets orally for three days [representing a total daily treatment dose of 1 gram/400 mg]), which has been shown to have hepatic clearing capabilities. Coartem® will be available for those intolerant to Malarone®. Daily telephone calls will be made to volunteers who decide to terminate prematurely. Signs and symptoms consistent with a malaria infection will be elicited. Follow-up care will be offered regardless of study participation if so desired by the participant. In the event that the volunteer refuses drug treatment, he/she will be contacted daily to assess signs and symptoms related to malaria until Day 28 after which, weekly phone calls will be made until Day 56.

7.6 Unscheduled Visit

The participants will be followed on an outpatient basis during the high-risk period for malaria development (Study Days 6-8) with inpatient observation for malaria during Days 9-19. Outpatient follow-up will continue on Day 21 (+3) and 29 (±2) post-CHMI and +28 (±3) days post-therapy. There is a small to negligible risk that a volunteer will have a delayed parasitemia beyond Day 21. In the event that a participant remains smear-negative at Day 21 (+3), the qPCR results will be examined to determine if patency is imminent and team members will determine whether to delay leukapheresis to maximize plasmablast collection (Day 7 post-treatment). Participants will be given cards to inform outside medical personnel that they have been infected with *P. falciparum* malaria. Volunteers without cellular phones will be issued Trak® phones to facilitate communication between the investigator and the volunteers. Each volunteer will have two modes of contact solicited as well as a designated point of contact (friend, relative, etc.) who can be reached in the event that the volunteer does not respond. Unscheduled visits will likely represent visits related to the onset of symptom development and will be recorded as an unscheduled visit in the CRF. Malaria smears and/or us-qPCR and a clinical exam will be performed.

8 STUDY PROCEDURES/EVALUATIONS

8.1 Clinical Evaluations

The following study procedures will occur for the CHMI. Also refer to Appendix C Tables C1, C2, C3 & C4.

8.1.1 Screening and enrollment:

8.1.1.1 Day –56 to –1: Screening and inclusion of all participants.

- Eligible participants will be provided general information pertaining to the study including purpose and study procedures. Interested parties will be asked to complete an Assessment of Understanding and sign a consent form. The following procedures will follow only after a signed consent form is obtained.
- A review of inclusion and exclusion criteria will be performed.
- A general medical history including targeted questions as to previous allergic reactions, travel history, history of splenectomy and/or sickle cell trait and cardiovascular disease risk assessment will be performed.
- A screening 12-lead ECG will be performed
- A medical exam with height, weight, and vital signs will be performed
- All current medications will be reviewed by study personnel
- A 7 mL sample of venous blood for safety evaluation (CBC, biochemistries (creatinine, AST, ALT) will be obtained from all study participants. Results will be considered valid within 56 days of study enrollment and will be repeated in the event that the volunteer was initially screened prior to this time point.
- An 11 mL sample of venous blood will be obtained for viral serologies (HIV, HbsAg, HCV), and sickle cell trait.
- Serum will be collected on all females to perform a serum β-HCG pregnancy test (unless written medical confirmation of sterility or amenorrhea (defined as greater than 1 year with medical evaluation) is provided)
- A unique study alpha-numeric identifier will be assigned to each participant
- **Repeat CHMI Subjects, only**: a 30 ml sample of venous blood will be obtained for a baseline PBMC collection.
- Screening will occur over 1-2 visits.

8.1.1.2 Day -56 to -1: Re-screening of repeat CHMI subjects

- A review of inclusion and exclusion criteria will be performed.
- An updated history and a targeted physical will be performed.
- All current medications will be reviewed by study personnel
- A 7 mL sample of venous blood for safety evaluation (CBC, biochemistries (creatinine, AST, ALT) will be obtained from all study participants. Results will be considered valid within 56 days of the date of CHMI. Transient laboratory abnormalities will not result in study disqualification if screening values met enrollment criteria.
- An 8 mL sample of venous blood will be obtained for HIV testing if more than a year has passed since the original screen.
- A 30 ml sample of venous blood will be obtained for a baseline PBMC collection.

8.1.1.3 Day 1: Mosquito Challenge, uninfectious (mock) or infectious (CHMI)

Prior to the mosquito challenge (mock or CHMI)

- All screening laboratory tests and ECG interpretation(s) will be reviewed. A positive HIV test is exclusionary.
- Eligibility criteria will be reviewed
- Interim (previous 2 week) antibiotic usage will be reviewed.
- Urine will be collected on all females to perform a urine β-HCG pregnancy test (unless written medical confirmation of sterility or amenorrhea (defined as greater than 1 year with medical evaluation) is provided)
- A 7 mL sample of venous blood for safety evaluation (CBC, biochemistries (creatinine, AST, ALT) will be obtained from all study participants prior to administration of the infectious challenge *(Note: The uninfectious CHMI will not require safety samples).*
- **Repeat CHMI Subjects, only:** 83 mL of venous blood will be collected and archived for research purposes on the first infectious CHMI (this also applies to those replacement volunteers for the core volunteer group); composed of 3 mL for gene expression and 80 mL for PBMC preservation. 53 mL of venous blood will be collected and will be archived for research purposes for CHMI #2-4; composed of 3 mL for gene expression and 50 mL for PBMC preservation for the purposes of cellular studies.
- Vital signs will be recorded (including an oral temperature, blood pressure, heart rate and respiratory rate).
- An updated history and a targeted physical will be performed.

Mock challenge or CHMI will be administered (Day 1)

After the mosquito challenge

- After mosquito exposure, the participants will be observed for a minimum of 30 minutes.

- Oral temperature, blood pressure, and pulse will be recorded.
- All solicited and unsolicited AE will be recorded.
- Participants will be instructed as to the symptoms of malaria and instructed to contact study personnel immediately should they manifest and signs or symptoms they perceive as serious.
- Participants will receive and be instructed on the completion of Memory aids.
- Participants will be counseled on mosquito avoidance behavior.
- Participants will be issued identification cards explaining that they have been exposed to malaria. If they do not own a cell phone, a “Trak phone” will be issued to facilitate contacting study personnel.

8.1.2 Subject follow-up:

8.1.2.1 Outpatient Post-Uninfected (Mock) Malaria Challenge Surveillance (Repeat CHMI Subjects, only)

- Participants will follow-up in clinic on Study Days 2, 4, 6, 8, 10, 13 and 21. Participants will be interviewed. Participants will use a Memory Aid on days 1-5, and solicited and unsolicited signs and symptoms will be recorded by Study Staff.
    - A review of all current medications (focusing on antibiotics) will be conducted
- Assessment of AE and SAE that have occurred since the last visit.
    - 3 mL of venous blood will be collected daily for gene expression studies.
    - 3 mL of venous blood will be collected for a CBC on Day 21 prior to leukopheresis
- 30 mL of venous blood will be collected on Days 6, 8, 13 (±1) and 21 (± 2) for PBMC separation

8.1.2.2 Outpatient Post-Infected Malaria Challenge Surveillance (Days 2, 4 and 6-8)

Repeat CHMI Subjects, only

- Participants will follow-up in clinic on Days 2, 4, and 6-8. Participants will be interviewed. Participants will use a Memory Aid on days 1-5, and solicited and unsolicited signs and symptoms will be recorded
- A review of all current medications (focusing on antibiotics) will be conducted
- Assessment of AE and SAE that have occurred since the last visit.
- 3 mL of venous blood will be collected on Days 6-8 for malaria diagnostics (PCR analysis)
- 3 mL of venous blood will be collected on Days 2, 4, 6 and 8 for gene expression studies
- 30 mL of venous blood will be collected on Days 6 and 8 (±1) for PBMC separation

Infectivity controls

- Participants will follow-up in clinic on Days 2, 4, and 6-8. Participants will be interviewed. Participants will use a Memory Aid on days 1-5, and solicited and unsolicited signs and symptoms will be recorded
- A review of all current medications (focusing on antibiotics) will be conducted
- Assessment of AE and SAE that have occurred since the last visit.
- 3 mL of venous blood will be collected on Days 6-8 for malaria diagnostics (PCR analysis)

All Volunteers, Repeat CHMI Subjects and Infectivity controls

- Daily clinical evaluations will begin on Day 9. Vital signs and a targeted clinical exam will be conducted.
- Participants will be reminded to contact study personnel immediately should they manifest any signs or symptoms they perceive as serious.
- Participants will be counseled on mosquito avoidance behavior.

8.1.2.3 **Days 9-19, Inpatient (or daily outpatient follow-up) Daily Post-malaria Challenge Surveillance**

All Volunteers, Repeat CHMI Subjects and Infectivity Controls

- Participants (CHMI #1-4) will be admitted for nightly observation during the period of time they are most at risk for the development of falciparum malaria
- The admission will be open in that participants will be able to leave the ward after blood draws from ~7 AM to 7 PM to attend to daily activities. They will be instructed to sleep on the ward. Participants will be interviewed. Assessment of signs and symptoms of malaria, AE and SAE that have occurred since the last visit will be recorded. Signs and symptoms attributable to malaria, cardiovascular symptoms (chest pain and shortness of breath, and unsolicited signs and symptoms will be recorded.
- Participants of CHMI #5 will continue to be followed on a daily outpatient basis.
- A review of all current medications (focusing on antibiotics) will be conducted.
- Vital signs and a targeted clinical exam will be conducted.
- A small quantity of blood (3 mL) will be obtained daily for malaria diagnostics (malaria blood smear and PCR analysis (for CHMI #1-4) and continue after treatment is initiated until 3 sequential malaria blood smears separated by 12 hours are documented as negative. Symptomatic individuals will have smears performed q 6-12 hours for the purposes of malaria diagnosis.
- For participants of CHMI #5, a small quantity of blood will be obtained daily for malaria diagnostics (malaria blood smear and us-qPCR analysis for core group volunteers and us-qPCR alone for infectivity controls). Due to the attenuation of malaria symptoms among the malaria-experienced volunteers and the lower threshold of detection and treatment for infectivity controls, it is unlikely that significant malaria symptoms will be detected. Symptomatic

individuals can continue to have diagnostics performed q 6-12 hours for the purposes of malaria diagnosis.

- Urine will be collected on all females to perform a urine β-HCG pregnancy test (unless written medical confirmation of sterility or amenorrhea (defined as greater than 1 year with medical evaluation) is provided) on the day of the first positive malaria smear.

If by Day 19, the study volunteer has not contracted malaria and is free of signs and symptoms of malaria, they will be discharged from the daily follow-up and will continue with scheduled outpatient clinical evaluations (**Section 8.1.2.5**below). Participants will be asked to contact study personnel if signs or symptoms consistent with malaria develop.

Repeat CHMI Subjects, only

- 3 mL of venous blood will be collected every other day (e.g., Study Days 10, 12, 14, 16, 18 until 7 days after the malaria-associated PBMC collection) for gene expression studies
- 30 mL of venous blood will be collected for the purpose of PBMC separation treatment day (±1). Ideally the blood will be collected prior to treatment, but for the safety of the volunteer if the blood collection is delayed treatment will still be started. -If the subject has not been treated by D13 then the timing of the PBMC collection will be based on their PCR results.
  - If they have not had a positive PCR by D13 then the PBMC collection should be done on D13.
  - If they have had a positive PCR (any detectable Ct measurement) by D13 then the PBMC collection should not be done until the person is smear positive (±1) or discharged from daily follow-up (±1) .

8.1.2.4 Malaria infection (Predicted to occur between Days 9-19)

All Volunteers, Repeat CHMI Subjects and Infectivity Controls

- Participants will be interviewed. Solicited and unsolicited signs and symptoms will be recorded.
- A review of all current medications (focusing on antibiotics) will be conducted.
- Vital signs and a targeted clinical exam will be conducted. Vital signs will be monitored q 4-6 hours.
- 10 mL of venous blood will be collected. The samples (7 mL) will be used to perform CBC, Biochemistries (creatinine, AST, and ALT), for malaria diagnostics (3 mL: malaria blood smear and PCR analysis). These diagnostic laboratories will be drawn on a daily basis as long as the malaria smear remains positive or for the duration of malaria therapy if the parasitemia becomes undetectable.
- The PCR analysis will be performed daily for the duration of the positive malaria smear and continue for two days after resolution of the visually detectable blood-stage parasitemia, or during directly observed thereapy in the case of infectivity controls for CHMI #5.
- Urine will be collected on all females at the time of malaria smear positivity to perform a β-HCG pregnancy test.

- Participants will receive Coartem® as therapy over 3 days (Four 20mg/120mg tablets as a single initial dose, four 20mg/120mg tablets again after 8 hours and then four 20mg/120mg tablets twice daily (morning and evening) for the following two days. Alternate therapy will be atovaquone/proguanil (Malarone®) as malaria treatment at four 250/100mg tablets orally for three days, representing a total daily treatment dose of 1 gram/400 mg.
- Ibuprofen at 200-600 mg q 6 hours and/or acetaminophen at 325-500 mg orally q 4-6 hours will be administered as needed to relieve symptoms such as fever, headache and body aches.
- Study participants will be discharged (from daily follow-up) after completion of oral antimalarial therapy x 3 days (for Infectivity Controls alone, CHMI #5) and the documentation of two sequentially negative malaria smears (with a 3rd completed as an outpatient, CHMI #1-4), or three sequentially negative malaria smears for core group volunteers after CHMI #5.
  - **Repeat CHMI subjects** will be asked to follow-up 7 days (+3) after the malaria-associated PBMC collection, and Day 29 (±2) and again +28 (±3) days following treatment dosing.
  - **Infectivity controls** will be asked to follow-up on Day 21 (±1), and Day 29 (±2) and again +28 (±3) days following treatment dosing.

- Malaria events and clinical malaria infection signs and symptoms: The malaria event is defined as the presence of a positive blood smear (or us-qPCR for infectivity controls in CHMI #5). Malaria signs and symptoms will be defined as the following: Malaise, chills/rigors, nausea, vomiting, dizziness, arthralgia, abdominal pain, fever, myalgia, and headache. These represent the top ten manifestations related to malaria illness. A comprehensive list would be beyond the scope of this trial. These events as well as any abnormalities of safety laboratory studies with onset up to 24 hours before or 96 hours after a positive malaria smear will not be captured as AEs. Smear-positive (either symptomatic or asymptomatic) malaria infection is a primary outcome measure of this study and is expected to produce a variety of systemic signs and symptoms. One of the secondary objectives is to determine whether the signs and symptoms of clinical malaria infections decrease in intensity and duration following repeat CHMI. Subjects who are malaria smear positive during the period of Day 6 through Day 29 (+/- 2) will be assessed regarding clinical malaria infection signs and symptoms. Documentation will follow the definitions described in Appendix B.

8.1.2.5 Outpatient Follow-up post-Inpatient Surveillance

Repeat CHMI Subjects

- Participants will follow-up in clinic 7 (+3) days after the malaria-associated PBMC collection or Day 21 (+3) if they remain aparasitemic on Day 19, Day 29 (±2) and +28 (±3) days post-therapy. Participants will be interviewed. Solicited and unsolicited signs symptoms (for those who do not acquire malaria) and symptoms will be recorded
- Leukapheresis will be conducted on all study participants +7 (+3) days after the malaria-associated PBMC collection or on Day 21 (+3) following CHMI (uninfected and infected) if aparasitemic on day 19. See Section 8.1.2.6 below.

Infectivity Controls

- Participants will follow-up in clinic Day 21 (±1), Day 29 (±2) and +28 (±3) days post-therapy. Participants will be interviewed. Solicited and unsolicited signs symptoms (for those who do not acquire malaria) and symptoms will be recorded

All Volunteers

- A review of all current medications (focusing on antibiotics) will be conducted.
- Assessment of signs and symptoms of malaria, AE and SAE that have occurred since the last visit.
- Vital signs and a targeted clinical exam will be conducted.
- A small quantity of venous blood (3 mL) will be obtained for malaria diagnostics (malaria blood smear and PCR analysis) on Days 21 (+3) repeat CHMI or 21(±1) infectivity controls and 29 (±2) for those who remain malaria-free.
- Urine will be collected on Study Day 29 (± 2) on all females to perform a urine β-HCG pregnancy test (unless written confirmation of sterility or amenorrhea (defined as greater than 1 year) is provided).
- 7 mL of venous blood will be collected on Day 28 (±3) days post-therapy. The sample will be used to perform a CBC and Biochemistries (creatinine, AST, and ALT). If the volunteer remains aparasitemic, this CBC and Biochemistry will occur on Day 29 (±2).

8.1.2.6 Outpatient Leukapheresis Procedure

Repeat CHMI Subjects, only

- Participants will follow-up in clinic +7 (+3) days after the malaria-associated PBMC collection or on Day 21 (+3) if still aparasitemic on Day 19 for planned leukapheresis
- A review of all current medications (focusing on antibiotics) will be conducted.
- Assessment of signs and symptoms of malaria, AE and SAE that have occurred since the last visit.
- Vital signs and a targeted clinical exam will be conducted.
- Venous blood (3 mL) will be drawn for the purposes obtaining a CBC.
- Leukapheresis criteria will be reviewed with the following inclusionary criteria. Volunteers who fail these criteria can continue in the study and be included in the analysis population providing they maintain adequate safety laboratories pre-CHMI.

  Adequate peripheral venous access

  No need for sedation

  Weight greater than 50 kg

  Hemoglobin > 9 g/dL for males and > 9 g/dL for females

  Platelet count > 100,000 /uL

- Vital signs will be monitored during leukapheresis procedures

8.2 Laboratory Evaluations

8.2.1 Clinical Laboratory Evaluations

Screening:

All Volunteers, Repeat CHMI Subjects and Infectivity Controls

The following laboratory tests will be performed at the time of participant screening and will be reviewed prior to enrollment into the protocol. Each laboratory result will be compared to upper and lower limit norms for the CVD's contracted laboratory system (Garcia Laboratories) (Appendix B). Results will be categorized as normal meaning that the values were within standard limits determined based on population results for the laboratories, or abnormal/positive based on outlying values beyond the upper or lower limit of normality. The Garcia Laboratories, Jackson, MI (or University of Maryland Reference Facility as a back-up) will perform these assays. It is estimated that 7 mL will be sufficient to complete the biochemical and hematological analysis. Additionally, 10 mL will be sufficient to complete viral serologies and sickle cell trait analysis.

- **Hematology:** hemoglobin, white blood cells (WBC) and, platelet count [3.0 mL]
- **Biochemistry:** Renal function test (creatinine), and liver enzymes (AST [Aspartate aminotransferase], ALT [Alanine aminotransferase]) [4.0mL]
- **Viral serologies:** Anti-Hepatitis C virus (HCV), Hepatitis B surface antigen (HbsAg) and Human Immunodeficiency Virus (HIV) will be recorded at baseline [8.0 mL]
- **Sickle Cell Trait:** Hemoglobin electrophoresis screening for sickle cell trait will be recorded at baseline [3 mL]

Enrollment:

All Volunteers, Repeat CHMI Subjects and Infectivity Controls

In addition to hematology and biochemistry that will be repeated at scheduled intervals; the following assays will be performed and repeated according to the schedule outlined in Tables (Appendix C Table C1-C4.

- **Pregnancy Test:** A serum pregnancy test will be performed at the clinical trial study site at the time of screening on all female participants regardless of age unless written medical demonstration of sterility or amenorrhea (defined as one year with medical evaluation) can be provided. A Food and Drug Administration (FDA)-approved testing kit for urine β-hCG pregnancy testing will be performed at the clinical trial study site on all female participants within 24 hours of the malaria challenge study or for re-screening. In addition, testing will be performed on the first day of a positive malaria smear and again on Visit 20 (+28 (±3) days after treatment) at the conclusion of active surveillance.

- **Blood malaria smear**: A small aliquot of blood (10 µL) will be placed upon microscope slides for the creation of thick malaria smears. The thick smear will be allowed to dry, lysed with distilled water and stained with Giemsa for analysis of intra-erythrocytic ring forms consistent with malaria. Trained investigators will examine five separate passes along the 1 cm axis of a blood smear using the 100x oil immersion lens of calibrated microscopes. This will be doubled to ten passes for symptomatic individuals. Ten passes performed by microscopists examines a total of 0.9-1.1 µL of blood (refer to standard operating procedures in the study manual of procedures (MOP) for full details). See **Table 2** for quantification of parasitemia. The peripheral blood smear provides comprehensive information on the stages, and the density of parasitemia with a sensitivity of 5 to 20 parasites/µL of blood for an experienced laboratory professional although this level can be much higher taking into account reader variability. These assays will be performed on-site in the Malaria Laboratory and Insectary of the CVD and the collective experience of the CVD staff is a sensitivity of 2 parasites/µL. The minimum acceptance criteria is NO false positive reads on thick smears and a positive smear will be defined as two unquestionable parasites present on smear. The above technique has proven to be highly effective and accurate with ~45% of individuals being diagnosed prior to malaria symptoms (personal experience, UMD). The slides will be stored for future review as necessary

Table 2: Parasitemia determined by light microscopy as adapted from Hanscheid. [71]

Parasitemia[a]	Parasites/µL[b]	Correlation
0.0001-0.0004%	2-20	Examine 300 HPF[c] to assure 0.75 µL is reviewed.
0.002%	100	Threshold at which clinical symptoms can be seen
0.2%	10,000	
2%	100,000	
2-5%	100,000-250,000	Hyperparasitemia, severe malaria
10%	500,000	Exchange transfusion may be considered

[a] Determined by thin smear analysis
[b] Determined by thick smear analysis. UMD sensitivity is 2 parasites/□L.
[c] HPF = High power field

8.2.2 Special Assays or Procedures

All Volunteers, Repeat CHMI Subjects and Infectivity Controls

- **12-lead ECG:** A 12-lead ECG will be performed at screening prior to enrollment for the purposes of evaluating for occult cardiovascular disease. All ECGs will be evaluated first by clinical investigators and subsequently by a board certified cardiologist at the University of Maryland. Screening ECGs will be interpreted by cardiology prior to challenge. If an abnormality is detected, cardiology interpretation and consultation will be sought. An abnormal ECG will be defined as follows: pathologic Q waves and significant ST-T wave changes; left ventricular hypertrophy; any non-sinus rhythm excluding isolated premature atrial contractions; right or left bundle branch block; or advanced (secondary or tertiary) A-V heart block.
- **PCR analysis:** A sensitive real-time ultra-sensitive, quantitative polymerase chain reaction (us qPCR) will be used for the detection of *P. falciparum* parasites. As we intend on quantifying parasitemia, us qPCR will be run off venous blood acquired contemporaneously with blood smears in EDTA tubes. PCR results will be reported as the Cycle Threshold (Ct) at which parasitemia is detected to standardize across all challenges. Optimization experiments have established a high degree of sensitivity. PCR primers will be based on the published sequence of the highly conserved,[72] stage specific [73] *P. falciparum* 18S ribosomal RNA gene. Primer sequences are identical to the corresponding sequence of the NF54 strain. Samples will be blinded and assays run daily. Each sample will be run in triplicate along with a water control. The data will be analyzed using the Applied Biosystem 7300 or 7500 Absolute Quantification Software. For the purposes of CHMI #5 alone, infectivity controls will be treated based upon two sequential us-qPCR positive values (> 12 hours apart but < 60 hours).

Repeat CHMI Subjects, only

- **Research Peripheral Blood Mononuclear Cells (PBMC):** An aliquot of blood will be drawn at the time of enrollment (Screening visit 1 or 2) and at periodic time points (30 mL on Study Days 6, 8, and treatment day (±1) and 7 (+3) days later. Ideally, the PBMC blood draw will be done prior to treatment, but if there is a delay in collection treatment will not be delayed for the safety of the volunteer. If a volunteer is aparasitemic until D13 a PBMC sample will be drawn then and again on D21 (+3) if they continue to remain aparasitemic. PBMC will be separated and cryopreserved. Additionally, venous blood will be saved for gene expression analysis to be performed on Days 1, 2 and every other day until leukapheresis. These studies will be done for research purposes only and will have no bearing upon the clinical management of volunteers who contract malaria. Samples will remain and be stored in liquid nitrogen vapor phase at facilities at the CVD. PBMC will be analyzed for immunophenotyping.
- **Leukapheresis:** In order to obtain sufficient plasma cells for adequate antibody diversity recognition, it will be necessary to obtain larger quantities of leukocytes than can be safely obtained by simple phlebotomy. These components can be safely obtained using leukapheresis procedures performed in the Apheresis Clinic of the Department of Transfusion Medicine in the UMB.
    - Methodology - The procedure will be carried out by trained members of the Apheresis Clinic. A CBC will be obtained within 24 hours of apheresis. Study investigators will supervise the procedure while investigators from the Department of Transfusion Medicine will be available for consultation. Leukocytes for *in* vitro investigation will be obtained using automated continuous flow leukapheresis technique, which requires two

venipunctures. During leukapheresis, whole blood is withdrawn from an antecubital vein and channeled into a cell separator where cellular and plasma fractions are separated by centrifugation. Leukocytes are directed into a collection bag and the red cells (and platelets and plasma) are returned to the donor. The extracorporeal circuit is anticoagulated with citrate (ACD-A) at a whole blood to anticoagulant ratio of 10:1 to 12:1. Maximum extracorporeal blood volume ranges from 300 to 600 mL, depending on the device used.

- The continuous flow leukapheresis procedure is performed, which requires two venipunctures. As a general rule, approximately 1 x 10^9 leukocytes are collected for each liter of whole blood processed during the procedure. From this, approximately 1 x 10^6 plasma cells are obtained. Approximately two to three hours are required for a 2-4 pass leukapheresis to collect 1-2 x 10^9 leukocytes
- The interval between successive leukapheresis donations will be at least 8 weeks, which is more than the 3-week interval required of most leukapheresis studies. No more than 7 total leukapheresis sessions will be conducted. These frequencies are less than the frequency of donations allowed by the Research Donor Program at the Clinical Center Department of Transfusion Medicine and substantially more conservative than that allowed by AABB and FDA standards, which limit the number of leukapheresis procedures to 24 per year. Exclusionary criteria will be reviewed (See Section 8.1.2.6). Other exclusions for leukapheresis are typically cardiovascular instability, severe anemia, severe coagulation disorder, and pregnancy, which are excluded under the current protocol.

8.2.3 Specimen Preparation, Handling, and Shipping

Blood will be collected from study participants by venipuncture up to 25 times during each of the 5 CHMI studies, including screening (in the event that a participant does not acquire malaria). The maximum amount of blood requested from any participant for standard collection during the study for research purposes will not exceed 550 mL over an eight-week period. However, additional blood may be obtained as deemed necessary by the investigators or clinicians to evaluate any illness or condition.

8.2.3.1 Instructions for Specimen Preparation, Handling, and Storage

Blood will be obtained by research staff as part of the clinical evaluation of the participant. Assays will be sent to the University of Maryland central laboratory processing or onto Garcia diagnostics depending on the nature of the testing. Research samples will be prepared, handled and stored according to the study manual of procedures (MOP). US-qPCR assays will be run at the Center for Vaccine Development.

8.2.3.2 Specimen Shipment

Specimen collected during the course of the study will be shipped according to the study MOP to Uniformed Services University of the Health Sciences, Bethesda MD, 20814. Specimen labeling requirements will also be included in the study MOP.

9 ASSESSMENT OF SAFETY

9.1 Specification of Safety Parameters

9.1.1 Primary safety measurements:

1. Reactogenicity events related to administration of CHMI will be collected Days 1-5.
2. Adverse Events (AEs) will be collected Days 1-Day 37-56 (i.e. Day 28 post-therapy)
3. The number of serious adverse events (SAEs) throughout the study period of Day 1-Day 56. Special attention will be paid to cardiac signs and symptoms post-CHMI that are assessed as serious.

9.1.2 Adverse Events

ICH E6 Good Clinical Practice Guidelines defines an Adverse Event (AE) as any untoward medical occurrence in a patient or clinical investigation subject administered a pharmaceutical product regardless of its causal relationship to the study treatment. An AE can therefore be any unfavorable and unintended sign (including an abnormal laboratory finding), symptom, or disease temporally associated with the use of medicinal (investigational) product. AEs can also be an exacerbation of pre-existing conditions or events, intercurrent illnesses, or drug interaction. The occurrence of an adverse event may come to the attention of study personnel during study visits and interviews or by a study recipient presenting for medical care. Adverse events will be documented in terms of a medical diagnosis. When this is not possible, the adverse event will be documented in terms of signs and/or symptoms observed by the investigator or reported by the subject at each study visit. AEs occurring while on study will be documented appropriately regardless of relationship. Pre-existing conditions or signs and/or symptoms (including any which are not recognized at study entry but are recognized during the study period) present in a participant prior to the start of the study will be recorded on the participant's CRF and will be recorded as an AE if deterioration or exacerbation in the condition occurs during the study. Any hospitalization other than the planned inpatient evaluation for the malaria event will be considered a serious adverse event. Information to be collected include event description, date of onset, clinician's assessment of severity, relationship to study product (assessed only by those with the training and authority to make a diagnosis), and date of resolution/stabilization of the event. All AEs will be followed to adequate resolution or stabilization.

All AEs must be graded for severity and relationship to study product.

NOTE: Positive malaria blood smears and clinical malaria infection signs and symptoms are NOT collected as adverse events (see Section 8.1.2.4).

Severity of Adverse Events: AEs will be assessed by a licensed study physician listed on the Form FDA 1572 as the site principal investigator or appropriate sub-investigator and graded for severity using the toxicity table in Appendix B and if the event is not listed in the toxicity table, the grading will be done according to the functional scale for "all other conditions" which is:

- Mild (Grade 1): No interference with activity.
- Moderate (Grade 2): Some interference with activity not requiring medical intervention.
- Severe (Grade 3): Prevents daily activity and requires medical intervention.

Changes in the severity of an AE should be documented to allow an assessment of the duration of the event at each level of intensity to be performed. Adverse events characterized as intermittent require documentation of onset and duration of each episode.

9.1.3 Relationship to CHMI

The degree of certainty with which an adverse event can be attributed to the administration of *P. falciparum* during the malaria challenge (or alternative causes, e.g., concomitant medications, etc.) will be determined by how well the event can be understood in terms of one or more of the following:

- The incident having often been reported in literature from previous malaria challenge events.
- The incident being temporally associated with malaria challenge.

Relationship to study products: The clinician's assessment of an AEs relationship to test article (Pf infected mosquitoes) is part of the documentation process, but it is not a factor in determining what is or is not reported in the study. If there is any doubt as to whether a clinical observation is an AE, the event should be reported. All AEs must have their relationship to study product assessed using the terms: related or not related. In a clinical trial, the study product must always be suspect. To help assess, the following guidelines are used.

- Related– there is a reasonable possibility that the study product caused the adverse event. Reasonable possibility means that there is evidence to suggest a causal relationship between the study product and the adverse event.
- Not Related– there is not a reasonable possibility that the administration of the study product caused the event.

In order to accommodate the industry partner, all AEs will also be graded according to a grade scale. Details on the scale will be found in the MOP. The grade scale will not be used for any decision making in this trial, nor affect the conduct of the trial in any way. Before database lock and after all data has been collected and monitored, the data center will conduct a one-time reconciliation between the two grading systems

9.1.4 Reactogenicity – solicited events related to administration of CHMI

Reactogenicity events related to the administration of the malaria challenge Day 2 through Day 5 and will be graded in severity by the clinician using the toxicity table in Appendix B. For signs or symptoms not listed in the table, the following functional scale will be used to grade the severity:

Mild (Grade 1) - No interference with activity

Moderate (Grade 2) - Some interference with activity not requiring medical intervention

Severe (Grade 3) - Prevents daily activity and requires medical intervention

9.1.5 Malaria events and Clinical malaria infections

The malaria event is defined as the presence of a positive blood smear. These events and clinical malaria infections will not be captured as AEs or SAEs. Smear-positive (either symptomatic or asymptomatic) malaria infection is a primary outcome measure of this study and is expected to produce a variety of systemic signs and symptoms. One of the secondary objectives is to determine whether the signs and symptoms of clinical malaria infections will decrease in intensity and duration following repeat CHMI. Volunteers who are malaria smear positive during the period of Day 6 through Day 28 will be assessed regarding clinical malaria infection using the toxicity table in Appendix B.

Due to the overlap of the surveillance period between CHMI and the expected time to developing a malaria event, signs and symptoms that develop after the first 5 days will likely be ascribed to the malaria event (positive blood smear).

9.1.6 Serious Adverse Events

An adverse event or suspected adverse reaction is considered "serious" if, in the view of either the clinical site principal investigator or sponsor, it results in any of the following outcomes:

1. *Death*:
2. *A life threatening adverse event**:
3. *Inpatient hospitalization or prolongation of existing hospitalization:* Defined as an event requiring hospitalization other than the inpatient observation already outlined as part of the protocol (Days 9-19).
4. *A persistent or significant incapacity or substantial disruption of the ability to conduct normal life functions.*
5. A congenital anomaly/birth defect:
6. Important medical events that may not result in death, be life threatening, or require hospitalization, may be considered serious when, based upon appropriate medical judgment, the event may jeopardize the patient or subject and may require medical or surgical intervention to prevent one of the outcomes listed above. Examples of such medical events include allergic bronchospasm requiring intensive treatment in an emergency room or at home, blood dyscrasias or convulsions that do not result in inpatient hospitalization, or the development of drug dependency or drug abuse.

* Life-threatening adverse event. An adverse event is considered "life-threatening" if, in the view of either the investigator or sponsor, its occurrence places the patient or subject at

immediate risk of death. It does not include an adverse event that, had it occurred in a more severe form, might have caused death.

All SAEs will be:

- Assessed for severity and relationship to study product and alternate etiology (if not related to study product) by a licensed study physician listed on the Form FDA 1572 as the site principal investigator or sub-investigator Day 1 through Day 56.
- Recorded on the appropriate SAE form and eCRF.
- Followed through resolution or stabilization by a licensed study physician listed on the Form FDA 1572 as the site principal investigator or sub-investigator.
- Reviewed and evaluated by an SMC, Independent Safety Monitor (ISM), DMID, and the IRB, if indicated.

9.1.7 **Abnormal Laboratory Test Values**

Collection of laboratory data will be limited to those laboratory parameters that are relevant to safety, study outcome measures, and/or clinical outcome. Garcia Laboratories of Jackson, MI will be responsible for most testing results (with University of Maryland and Pharmaron laboratories as a back-up). In the event of a laboratory abnormality, the laboratory test may be repeated once to ensure accurate levels. Laboratory test abnormalities will be analyzed based on the grading scale in the toxicity table in Appendix B.

9.2 Timing for Assessing, Recording, and Analyzing Safety Parameters

9.2.1 **Uninfected mosquito challenge (repeat CHMI cohort)**

- Day 2 (post-CHMI challenge) through Day 5 – collection of reactogenicity related to administration which includes local site pain, tenderness, pruritus, edema, erythema and systemic event of fever.
- Day 1 through Day 56 – collection of unsolicited adverse events and SAEs.

9.2.2 **Infected mosquito challenge periods (repeat CHMI and Control Cohorts)**

- Day 2 (post-CHMI challenge) through Day 5 – collection of reactogenicity events related to the administration of the CHMI in all subjects.
- Day 1 through Day 37-56 (+ 28 days post-therapy) – collection of unsolicited adverse events and SAEs in all subjects.

9.3 Reporting Procedures

The Principal Investigator will ensure documentation of solicited reactogenicity Study Day 2-5 and unsolicited AEs and SAEs from the first administration of study product Study Day 1 through Study Day 56. SAEs will be reported by telephone, E-mail or fax within 24 hours of site awareness to DMID PVG and to the ISM. The IRBs listed below will be notified within 48 hours. In addition, Grade 3 AEs that meet the halting criteria for the study will be reported to the ISM (See **Section 9.5**). A written report will follow within 3 working days of the event. SAEs will be reported by the PI or co-investigator to the following:

- Human Research Protections Program Office, Uniformed Services University of the Health Sciences, 4301 Jones Bridge Road, Room A2051, Bethesda, MD 20814, Phone: (301) 295-9534, E-mail: leodayan.bojanowski@usuhs.edu
- Human Research Protections Office (University of Maryland IRB): Tel: (410) 706-5037, Fax: (410) 706-4189, email: HRPO@som.umaryland.edu.

9.3.1 Reporting of Serious Adverse Events

Any AE that meets a protocol-defined serious criterion must be submitted within 24 hours of site awareness on an SAE form to the DMID Pharmacovigilance Group, at the following address:

DMID Pharmacovigilance Group
Clinical Research Operations and Management Support (CROMS)
6500 Rock Spring Dr. Suite 650
Bethesda, MD 20817, USA
SAE Hot Line: 1-800-537-9979 (US) or 1-301-897-1709 (outside US)
SAE FAX Phone Number: 1-800-275-7619 (US) or 1-301-897-1710 (outside US)
SAE Email Address: PVG@dmidcroms.com

Other supporting documentation of the event may be requested by the DMID Pharmacovigilance Group and should be provided as soon as possible.

The DMID medical monitor and clinical protocol manager will be notified of the SAE by the DMID Pharmacovigilance Group. The DMID medical monitor will review and assess the SAE for regulatory reporting and potential impact on study subject safety and protocol conduct.

At any time after completion of the study, if the investigator becomes aware of an SAE that is suspected to be related to study product, the investigator will report the event to the DMID Pharmacovigilance Group.

9.3.2 Regulatory Reporting for Studies Conducted Under DMID-Sponsored IND

Following notification from the investigator, DMID, the IND sponsor, will report any suspected adverse reaction that is both serious and unexpected. DMID will report an adverse event as a suspected adverse reaction only if there is evidence to suggest a causal relationship between the drug/biologic and the adverse event. DMID will notify FDA and all participating investigators (i.e., all investigators to whom the sponsor is providing drug/biologic under its INDs or under any investigator's IND) in an IND safety report of potential serious risks from clinical trials or any other source, as soon as possible, but in no case later than 15 calendar days after the sponsor determines that the information qualifies for reporting as specified in 21 CFR 312.32. DMID will also notify FDA of any unexpected fatal or life-threatening suspected adverse reaction as soon as possible but in no case later than 7 calendar days after the sponsor's initial receipt of the information. Relevant follow-up information to an IND safety report will be submitted as soon as the information is available. Upon request from FDA, DMID will submit to FDA any additional data or information that the agency deems necessary, as soon as possible, but in no case later than 15 calendar days after receiving the request.

All serious events designated as "not related" to study product(s) will be reported to the FDA at least annually in a summary format.

9.3.3 Regulatory Reporting for Studies Not Conducted Under DMID-Sponsored IND

Not applicable

9.3.4 Other Adverse Events (if applicable)

Not applicable

9.3.5 Reporting of Pregnancy

All pregnancies that develop in participants within the 29-day window of the CHMI event (day of challenge plus 28 days) will be reported by the Investigator to the Sponsor and IRBs within 24 hours of learning of its occurrence. The pregnancy will be documented on the Pregnancy Reporting Form. All study-mandated blood samples will be obtained and the participant will continue in follow-up for safety events. Pregnancies will be followed until 30 days after delivery to determine outcome, including spontaneous or voluntary termination, details of the birth, and the presence or absence of any birth defects, congenital abnormalities, or maternal and/or newborn complications.

9.4 Type and Duration of Follow-up of Subjects after Adverse Events

Participants who experience AEs that are related to the study product will be followed to resolution or stabilization.

9.5 Halting Rules

Participant safety data will be reviewed on an ongoing basis. A decision to proceed or to terminate enrollment, CHMI, or the trial will be made in consultation, NIH/NIAID/DMID, and the study investigators. It is expected that Grade 3 laboratory abnormalities will occur in the setting of some malaria events. This is particularly relevant for a reduction in platelets or transient elevations in liver function tests. These laboratory abnormalities generally resolve quickly and without sequelae. Halting rules will not carry over from CHMI to CHMI but instead will reset for each subsequent challenge through Day 56 and will be applied accordingly.

1. One or more participants experience a SAE (as defined in **Section 9.1.6.**) that is determined to be related to the study product administration
2. Two or more participants experience anaphylaxis as determined by the study site physician to be categorized as level 1, 2, or 3 of the Brighton "anaphylaxis" Case Definition within 24 hr of the mosquito challenge[74] (See **Appendix D**).
3. Two or more participants experience an unsolicited Grade 3 local skin adverse event characterized by ulceration, abscess, or necrosis associated with the malaria challenge.
4. Occurrence of the same Grade 3 systemic AE in two or more volunteers that is determined to be related to the malaria challenge (but not to a smear-positive or subsequent smear-positive malaria event deemed to be the etiology of symptoms) and cannot be explained by another diagnosis unrelated to challenge.
5. Two or more participants experience the same Grade 3 laboratory abnormality in the absence of a diagnosis of malaria (or closely aligned subsequent positive smear) that is determined to be related to study product administration.
6. The Investigator may, using discretion, ask for the study to be placed on hold and a meeting with the ISM be held for any single event or combination of multiple events which, in professional opinion, jeopardize the safety of the subjects or the reliability of the data.

If any of the halting rules are met following any volunteer receipt of any study product, then this trial will not continue with the remaining enrollments or study product administration without a review by and recommendation from the SMC to proceed. The communications from the ISM will subsequently be forwarded by the investigators to the respective IRBs. DMID retains the authority to suspend additional enrollment and study interventions/ administration of study product during the entire trial, as applicable.

During clinic visit, the study personnel will ascertain that the reported severity of an AE by the participant meets the predefined criteria of the study of grading the severity of AEs.

If halting rules are triggered and the Internet is not available, then an immediate report will be made by phone and FAX.

If the study is halted, enrollment will also be halted. Should the study be halted, study-related procedures to measure safety and immunogenicity will continue as scheduled, but further administration of study product (i.e., malaria challenge) will not continue.

9.6 Safety Oversight (ISM plus SMC)

9.6.1 Safety Monitoring Committee (SMC)

This clinical trial will utilize an SMC, which is an independent group of experts that advises DMID and the study investigators. The primary responsibilities of the SMC will be to 1) periodically review and evaluate the accumulated study data for subject safety, study conduct and progress and 2) make recommendations to DMID concerning the continuation, modification or termination of the trial. The SMC will be composed of at least three voting members. Procedures for SMC data reviews will be defined in a SMC charter that will include membership, responsibilities, and the scope and frequency of data reviews. The SMC will operate on a conflict-free basis independently of the study team. DMID or the SMC may convene ad hoc meetings according to protocol criteria or if there are concerns that arise during the study. The SMC will have access to unblinded data during its closed session. After its assessment, the SMC will recommend continuation, modification, or termination of the clinical trial. The SMC will conduct reviews at the following time points:

- One of the secondary objectives is to determine whether clinical malaria infections decrease in intensity and duration following repeat CHMI. The SMC will review available safety data (including clinical malaria infections) for repeat CHMI and control groups, prior to CHMI 3 and CHMI 4, to monitor for unexpected worsening of clinical malaria infections or increase in the occurrence of hypersensitivity reactions with repeated exposures. As a dimunition in symptoms and prolonged parasitemia has been seen with CHMI #4, a fifth challenge is being added to the schedule to assess if full protection can be acquired after a 5th CHMI event.

- Final review meeting: 6 to 8 months after clinical database lock to review the cumulative unblinded safety data for the study. The data will be provided in a standard summary format. The SMC may be asked to provide recommendations in response to questions posed by DMID.

- Ad hoc review: may be in response to an anticipated safety issue such as a halting rule being met.

9.6.2 Independent Safety Monitor (ISM)

The ISM is a physician with relevant expertise whose primary responsibility is to provide DMID as safety assessment in a timely fashion.

Participation is for the duration of the DMID study and is a voluntary position that does not receive payment.

The ISM:

• Is in close proximity to the study site and has the authority and ability to readily access study participant records in real time.

• May be a member of the participating institution's staff but preferably be from a different organizational group within the institution.

• Should not be in a direct supervisory relationship with the investigator.

• Should have no direct involvement in the conduct of the study.

The ISM will:

• Sign a COI certification at the time they are asked to participate and provide updates to this information as needed.

• Receive reports of Serious Adverse Events (SAEs) from the site investigator and will be notified by email when DMID is notified of the SAE.

• Evaluate the SAE and report their clinical assessment to DMID, through DMID-CROMS SOCS in a timely manner using the attached report form and email the report to DMID-CROMS SOCS

• Communicate with the investigator at the participating site as needed.

• Review additional safety related events at the request of DMID.

• Provide additional information to DMID and/or the SMC by teleconference as requested.

10 CLINICAL MONITORING

In general, site monitoring is conducted to ensure that:

- human subjects' rights and well-being are protected;
- data are accurate, complete, and verifiable from source documents;
- the study complies with the protocol/amendment(s), International Conference on Harmonization (ICH) Good Clinical Practice (GCP) 5.18 and FDA 21 CFR 312.50, guidelines, and applicable regulatory requirements.

In order to ensure protocol compliance, monitoring will be conducted according to internal UMB clinical monitoring guidelines.

10.1 Site Monitoring Plan

Site monitoring is conducted to ensure that the human subject protections, study and laboratory procedures, study intervention administration, and data collection processes are of high quality and meet sponsor, ICH/GCP guidelines and applicable regulations, and that the study is conducted in accordance with the protocol, protocol-specific MOP and applicable sponsor standard operating procedures. DMID, the sponsoring agency, or its designee will conduct site-monitoring visits as detailed in the clinical monitoring plan.

Site visits will be made at standard intervals as defined by DMID and may be made more frequently as directed by DMID. Monitoring visits will include, but are not limited to, review of regulatory files, accountability records, eCRFs, informed consent forms, medical and laboratory reports, and protocol and GCP compliance. Site monitors will have access to the study site, study personnel, and all study documentation according to the DMID-approved site monitoring plan. Study monitors will meet with site principal investigators to discuss any problems and actions to be taken and document visit findings and discussions.

11 STATISTICAL CONSIDERATIONS

11.1 Study Hypotheses

This is a pilot observational study to determine if repeat CHMI induces immunity to the parasite and clinical symptoms of malaria and identify the immune responses associated with protection. We hypothesize that 1) repeat CHMI using the same *P. falciparum* strain will reduce pre-patent periods, parasitemia and clinical symptomatology during successive malaria episodes and 2) there will be a decrease in the inflammatory response due to the maturation of an effective adaptive immune response over the course of 2 to 3 CHMI that effectively eliminates the production of blood stage parasites.

11.2 Sample Size Considerations

The sample size was selected to retain 80% power assuming 7 of the 10 experimental subjects complete all 4 *P. falciparum* challenges and a 0.7 probability of protection in the experimental subjects and zero probability of protection in the control subjects. Under these conditions there is a 0.87 probability of correctly rejecting the null hypothesis that the protective efficacy for experimental and control subjects are equal under 2-sided Type I error of 0.05 11.2 using Boschloo's test.

11.3 Planned Interim Analyses (if applicable)

11.3.1 Safety Review

Interim review of safety data will be conducted at least annually as specified in Section 9.6.1. This review may include enrollment and demographic information, medical history, concomitant medications, summaries of solicited and unsolicited adverse events, malaria symptoms, clinical laboratory values, and physical assessments. Serious adverse events, if any, will be described. Halting rules will be assessed on an ongoing basis.

In addition, safety data will be summarized after each CHMI for investigator review. These reports will also include the efficacy analyses described in Section 11.4.3 and will be prepared after 28 days of CHMI follow-up.

11.3.2 Efficacy Analysis

Efficacy analyses will be conducted 28 days after each infectious CHMI, starting with CHMI 2. Boschloo's test will be used to test for a difference in the proportion of subjects testing positive for malaria between control subjects and those who previously received CHMI. At each of the three analysis time points, the test will be done twice, once for each measure (blood smear or qPCR).

Results from these interim analyses will not be used to halt the trial. As the emphasis of this Phase I trial is exploratory rather than confirmatory, adjustment for multiple testing will not be performed.

11.4 Final Analysis Plan

11.4.1 Demographic Summaries

Demographic information (age, gender, race, and ethnicity) will be numerically summarized overall and by study arm. The mean age (plus minimum, maximum, and standard deviation) will be summarized for enrolled subjects, overall and by study arm.

11.4.2 Safety Analysis

Adverse events will be classified by MedDRA® System Organ Class and Preferred Term, severity, and relationship to study treatment. Malaria signs and symptoms will be summarized, but will not be considered adverse events for subjects testing positive for malaria since malaria is the expected outcome of the intervention. Serious adverse events will be described. A complete listing of adverse events for each subject will provide details including severity, relationship to study product, onset, duration, and outcome. Laboratory results will be summarized by laboratory parameter, severity, study day, and study arm. A series of graphical displays will summarize clinical signs and symptoms of malaria by maximum severity, study day, CHMI, and/or symptom to illustrate whether these symptoms decrease in intensity and duration following repeat CHMI.

11.4.3 Protection against *P. falciparum* parasites (Primay Objective)

Volunteers will be monitored for the presence of blood stage *P. falciparum,* parasites daily from days 5-18 after each CHMI to determine if repeat CHMI induces protection against the parasite. Parasites will be quantitated by Giemsa-stained blood smear and quantitative-PCR using 18S ribosomal probe[35,43]. The results of the RT-qPCR will be reported as the Ct value at which a detectable response is measured. A significant reduction in the number of volunteers that become parasite positive following a repeat CHMI in comparison with the infectivity controls would indicate a protective response. The comparison will be performed using Boschloo's test. Boschloo's test is an unconditional exact test appropriate for two-by-two contingency tables. Unlike Fisher's exact test, Boschloo's test does not condition on the row and column margins of the table, and it is uniformly more powerful than Fisher's exact test.[75,76] Three comparisons will be performed using Boschloo's test, one each for the second, third, and fourth infectious CHMIs. In addition, the mean time to positive qPCR and mean time to positive blood smear will be presented separately for each group along with standard deviation for each sequential CHMI. The first day a positive qPCR or blood smear is detected and the growth pattern of blood stage parasite as

quantified by qPCR will also be compared for each sequential CHMI. Graphs will compare time trends in parasitemia between subjects receiving repeat CHMI and controls. A separate graph will be created for each sequential CHMI. The parasitemia data will be correlated with oral temperatures and reported clinical symptoms following each subsequent CHMI and compared with the response of the infectivity controls.

11.4.4 Immune response analysis (Secondary Objective)

In contrast to the parasite protection analysis the focus of the immune analysis is to evaluate the maturation of an individual's immune response following each sequential CHMI and compare that to the same individual's pattern of parasitemia and clinical symptoms. Additional exploratory studies will include whole blood transcriptional and plasmablast Ig repertoire analysis. For the immune response analysis each volunteer will serve as their own control and changes will be monitored following the sequential challenges. Response patterns will also be compared between volunteers to identify general trends.

Antibody reactivity agasinst Pf pre-erythrocytic antigen, CSP, and Pf liver and erythrocytic stage antigen, GLURP (Secondary Objective). The development of antibodies that recognize the pre-erythrocytic and erythrocytic parasite stages will be assessed in plasma samples obtained over the course of the clinical trial. Specifically, plasma samples will be tested for the presence of antibodies that bind synthetic peptides representing the Circumsporozoite protein (CSP) expressed on the surface of the Pf sporozoite or Glutamate-rich protein (GLURP) expressed by both liver- and asexual blood-stage parasites [63,64]. The CSP peptide will be based on the repeat region of CSP with the amino acid sequence (NANP)8 (GenScript) and the GLURP peptide will be CGDKNEKGQHEIVEVEEILPEGC amino acid sequence (GenScript) of the R2 repeat region[63,64]. From previous CHMIs these have been found to be sensitive markers of parasite exposure. Antibody reactivity will be tested by ELISA using horseradish peroxidase (HRP)- labeled goat anti-human IgG and HRP- labeled goat anti-human IgM. Antibody reactivity will be defined as the optical density at 410 nM (OD_{410}) of a 1/300 dilution of the volunteers plasma sample minus the OD_{410} of a 1/300 dilution of the volunteer's baseline sample. The plasma sample obtained prior to the volunteer's first mosquito challenge will be used as the baseline sample. The resulting normalized OD_{410} will be tabulated per subject and time point as well as per time point across subjects using the minimum, Q1, median, 95% bootstrap CI of the median, Q3, and maximum. Results will be visualized using time trend plots for individual subject results and across subjects using the medians and 95% bootstrap CIs. Sequential CHMI time point results will be compared using a two-sided Wilcoxon signed-rank test to identify general trends in the data. ELISA summaries will be reported among the humoral immunogenicity analysis population, defined as those who received 3 or more infectious challenges.

Exploratory Objectives

PBMC immunophenotyping, RNA transcript analysis and plasmablast Ig repertoire analysis will be performed after the conclusion of this study. To facilitate direct comparison

of the samples obtained at different times, the samples will be stored until the study is completed and analyzed as a set. Due to rapidly advancing technology for sequence analysis it is possible that by the end of the study novel methods will be available to evaluate the samples. However, the current plan is to determine the PBMC immunophenotyping using flow cytometry, RNA expression profile using the Illumina Human HT-12 Expression BeadChip and the plasma blast Ig repertoire will be determined by sequencing heavy and light chain variable regions after amplification using reverse transcriptase PCR. Prior to receipt of the exploratory data, a Statistical Analysis Plan that specifies all planned exploratory analyses will be developed. In the following an abbreviated version of the planned exploratory analysis is provided.

Analysis of percentage of activated PBMCs The PBMC immunophenotyping will provide an overview of the response of peripheral blood leukocytes to the transition of the parasite from the skin to the vasculature and liver and then back to the blood stream. Importantly, the results will provide the first analysis of the timing and maturation of these responses in an individual through defined repetitive parasite exposures. We hypothesize that each person's response will change with subsequent parasite exposures, leading ultimately to a decrease in proinflammatory cytokine production and thus a decrease in fever. The immunophenotyping results reported as percentage of activated PBMCs (B-cell, T-cell, NK-cell, monocyte and dendritic cells) will be obtained using Flowjo analytical software (Flowjo Enterprise, www.flowjo.com) or similar software and compared with the clinical data.[77] Resulting percentages will be tabulated per subject and time point as well as per time point across subjects using the minimum, Q1, median, 95% bootstrap CI of the median, Q3, and maximum. Results will be visualized using time trend plots for individual subject results and across subjects using the medians and 95% bootstrap CIs. Sequential CHMI time points results will be compared using a two-sided Wilcoxon signed-rank test to identify general trends in the data.

Particular attention will be paid to the timing of the first evidence of activation of the dendritic and monocyte populations, as well as the CD8+ T-cells and CD4+ T-cells and the appearance of circulating plasmablasts using SPICE or similar software[78]. In subsequent follow up studies plasma isolated before and after a volunteer has reduced susceptibility to fever and/or parasites will also be functionally evaluated for the ability to enhance phagocytosis or complement-mediated lysis or inhibit adhesion to human microvascular endothelial cells[79,80] or growth as assayed by the growth inhibition assay (GIA).[81]

Plasmablast immunoglobuline gene repertoire (Exploratory Objective) To evaluate the Ig repertoire, the Ig variable region sequence data from each plasmablast sample will first be filtered for the presence of the primer sequence on both ends and then sorted into groups based on the primer sets used for the different types of heavy and light chain V exons. The sequences in each group will be compared to determine the number of identical reads, and these sequences will be grouped to calculate the number of identical reads for each sequence. Alignment of rearranged IgH or IgL sequences to germ line V, D and J or V and J segments, respectively and determination of V-D and D-J junctions or V-J junctions, respectively, will be done using the IgBLAST algorithm (Ye, Jian, et al. "IgBLAST: an

immunoglobulin variable domain sequence analysis tool." Nucleic acids research 41.W1 (2013): W34-W40.). The distinct nucleotide and corresponding amino acid sequences will be aligned against the IMGT reference database using IMGT/HighV-QUEST (Lefranc, Marie-Paule, et al. "IMGT, the international ImMunoGeneTics database." Nucleic acids research 27.1 (1999): 209-212) to evaluate V, D and J allele usage and identify differences with the germ line sequence. This information will allow the determination of the sequence of hypervariable complementary binding region 3 and other regions modified by somatic hypermutation. Unique sequences will be identified using a combination of V, D, J, VD junction and DJ or VJ junction bases. Relative abundance of each sequence will be tracked across time points. Nonrandom representation of pairs of V, D or J segments in the IgH sequence data sets will be evaluated by comparing the frequency of pair wise combinations of segments to the product of the individual segment frequencies.Sequence data will be analysed using the online VDJ software (www.VDJserver.org, Shugay M, et al. (2015) VDJtools: Unifying Post-analysis of T Cell Receptor Repertoires. PLoS Comput Biol 11(11): e1004503) . The extent of amino acid variability in samples collected before and after sequential malaria exposure will also be evaluated by calculating the alignment relative entropies, which measure the difference between two probability distributions at these positions. In this case, one distribution represents the sequence distribution in the different samples at the variable positions, and the other represents the sequence distribution from the random background, which can be derived from proteins in IMGT and the SWISS-PROT database (Boeckmann, Brigitte, et al. "The SWISS-PROT protein knowledgebase and its supplement TrEMBL in 2003." Nucleic acids research 31.1 (2003): 365-370). Replicate samples of known sequence composition as well as independent PCR reactions from the plasma cell library will also provide important controls for intra-sample variability data. Regions with large differences in relative entropy will be analyzed statistically to see if there is a significant difference in frequencies for each presenting sequence motif.

Gene Expression Profiling (Exploratory Objective) For expression profiling RNA transcripts with significant expression will be identified using the statistical program SAM (Significance Analysis of Microarrays) or similar[82], with the false-discovery rate (FDR) <0.05%, and the genes will be organized by annotation to identify overrepresented signaling/transcription pathways or gene families such as cytokines. Unsupervised hierarchical cluster analysis will be used to group genes with similar gene expression patterns over time and to compare the expression data with parasitemia and fever measurements. Gene clusters will also be examined for enrichment in known cellular pathways using Gene Set Enrichment Analysis (GSAE) or other similar pathway enrichment tools. TheMolecular Signatures Database (*MSigDB*) will be used as pathway reference database including KEGG, Reactome, Gene Ontology, and Immunologic Signature Sets. The data will be compared with the PBMC population profiles to determine the cells involved in the response. Transcript increases that precede changes in cell populations will be targeted for further analyzed for a possible regulatory role. Together, the data should provide an overview of the stimulation and maturation of the immune response following repeated parasite infections. Those responses temporally associated with a decrease in fever or blood stage parasitemia will be targeted for further analysis to

determine their role in protective immunity. RNA expression patterns will also be compared between volunteers to determine whether there are common responses to the parasite. The small number of volunteers in this discovery study may limit the strength of this analysis, but if similar specific pathways are stimulated in several different volunteers, these pathways could be targeted for further evaluation in a larger study. For example, if a subset of volunteers has a marked increase in TGFβ, as found in a previous study of cytokine expression after a single Pf challenge[57], then the course of subsequent infections in these volunteers would be compared. If this is associated with higher parasitemia or a distinct immune response pattern throughout the study, then this pattern could be evaluated more directly in future challenge or field studies. In these future studies, genetic analysis of the volunteers with distinct response patterns could be included to identify possible underlying mechanisms for the differences.

12 SOURCE DOCUMENTS AND ACCESS TO SOURCE DATA/DOCUMENTS

The site will maintain appropriate medical and research records for this trial, in compliance with ICH E6, Section 4.9, and regulatory and institutional requirements for the protection of confidentiality of subjects. As part of participating in a DMID-sponsored, DMID-affiliated, or manufacturer-sponsored study, the site will permit authorized representatives of the DMID, its designees, and appropriate regulatory agencies to examine (and when required by applicable law, to copy) clinical study records for the purposes of quality assurance reviews, audits, monitoring and evaluation of the study safety and progress. These representatives will be permitted access to all source data, which include, but are not limited to, hospital records, clinical and office charts, laboratory notes, memoranda, evaluation checklists, pharmacy dispensing records, recorded data from automated instruments, copies or transcriptions certified after verification as being accurate and complete, microfiches, photographic negatives, microfilm or magnetic media, x-rays, and subject files and records kept at the pharmacy, at the laboratories, and medico-technical departments involved in the clinical trial. Source data are all information, original records of clinical findings, observations, or other activitie*s in a clinical trial necessary for the reconstruction and evaluation of the trial. Data collection* forms will be derived from the eCRFs and be provided by the SDCC.

13 QUALITY CONTROL AND QUALITY ASSURANCE

The Quality Management plan will comply with DMID Clinical Quality Management Plan (CQMP) policy; and the implementation of that plan benefits the internal site audits by:

- Supporting substantive performance measurements/findings/corrective actions, as required, and
- Providing data to support reporting requirements, as applicable.

The University of Maryland Center for Vaccine Development core Quality Management (QM) Plan is accepted by DMID quality management oversight and is in place onsite, and available upon request. As defined in the core QM plan, a separate protocol-specific clinical research QM plan outlining the sample size, priority of protocol review, frequency of quality assurance audits, communication of findings, and annual review will be prepared for DMID QMP reviewers.

Clinical site monitors will verify that the clinical trial is conducted and data are generated, documented (recorded), and reported in compliance with the protocol, GCP, and the applicable regulatory requirements. These monitoring visit reports will be submitted to DMID.

The investigational sites will provide direct access to all trial-related sites, source data/documents, and reports for the purpose of monitoring and auditing by the sponsor, and inspection by local and regulatory authorities.

14 ETHICS/PROTECTION OF HUMAN SUBJECTS

14.1 Ethical Standard

The investigators will ensure that this study is conducted in full conformity with the Declaration of Helsinki, or with the ICH GCP regulations and guidelines, whichever affords the greater protection to the participant.

The investigators will also ensure that this study is conducted in full conformity with the principles of the Belmont Report: Ethical Principles and Guidelines for the Protection of Human Subjects of Research of the National Commission for the Protection of Human Subjects of Biomedical and Behavioral Research (April 18, 1979) and codified in 45 CFR 50 and 56. The Institution will hold a current FWA issued by OHRP for federally funded research.

14.2 Institutional Review Board

Prior to enrollment of participants into this trial, the protocol and protocol-related documents including the informed consent form will be reviewed and approved by the Uniformed Services University of the Health Sciences and University of Maryland IRBs

The responsible official for the IRB will sign the IRB letter of approval of the protocol prior to the start of this trial and a copy will be provided to DMID. Notification of the IRB's composition and the institutions Federal Wide Assurance number will be provided to DMID. The University of Maryland IRB currently holds and will maintain a U.S. FWA issued by OHRP for the entirety of this study.

Should amendments to the protocol be required, the amendments will be written by the sponsor and provided to the investigator for submission to the IRB.

Participants will be compensated for their participation in this study. Compensation will be in accordance with the local IRB's policies and procedures, and requires IRB approval.

14.3 Informed Consent Process

The Investigator will choose participants in accordance with the eligibility criteria detailed previously. The investigator will not exercise selectivity so that bias is prevented. All participants must sign an informed consent form that complies with the requirements of both 21 CFR Part 50 and Health Insurance Portability and Accountability Act (HIPAA) before entering the trial. A consent form that complies with the requirements of 21 CFR Part 50 will be used.

Prior to the trial, participants will receive a comprehensive explanation of the proposed parenteral challenge product, including the nature and risks of the trial, any known adverse events associated with the trial product, the investigational status of the components, the

procedure and risks of malaria challenge, and the other elements that are part of obtaining proper informed consent. Participants will also receive a detailed explanation of the proposed use and disclosure of their protected health information, including specifically their biological specimens. Participants will be allowed sufficient time to consider participation in the trial, after having the nature and risks of the trial explained to them. The consent form must not include any exculpatory statements.

Sanaria will provide the investigator, in writing, any new information that bears significantly on the participants' risk to receive the investigational product. This new information will be communicated by the investigator to participants who consent to participate in the trial in accordance with IRB requirements. The informed consent document will be updated and participants will be re-consented, if necessary.

Site staff may employ recruitment efforts prior to the participant consenting; however, before any protocol-specific procedures are performed to determine protocol eligibility, an informed consent form must be signed. Participants will be given a copy of all consent forms that they sign.

The informed consent process will be initiated before a volunteer agrees to participate in the study and should continue throughout the individual's study participation. The subject will sign the informed consent document before any procedures are undertaken for the study. A copy of the signed informed consent/assent document will be given to the subject or the legal guardian for their records. The consent will explain that subjects may withdraw consent at any time throughout the course of the trial. Extensive explanation and discussion of risks and possible benefits of this investigation will be provided to the subjects in understandable language. Adequate time will be provided to ensure that the subject has time to consider and discuss participation in the protocol.

The consent form will describe in detail the study interventions/products/procedures and risks/benefits associated with participation in the study. By signing the informed consent form, the participant agrees to complete all evaluations required by the trial, unless the participant withdraws voluntarily or is terminated from the trial for any reason. Volunteers who concluded study operations after CHMI #4, but express interest in participation within CHMI #5 (i.e., core group volunteers), will be re-consented. Accordingly, the age distribution has also been increased to accommodate volunteers who enrolled at age 45 years, inclusive, but have aged beyond this time limit (no expectation that this will increase risk to the volunteers as recruitment to age 50 years is commonly used in malaria vaccine trials.

14.3.1 Informed Consent/Assent Process (in Case of a Minor)

Not applicable

14.4 Exclusion of Women, Minorities, and Children (Special Populations)

All healthy adults between the ages of 18 to 50 years of age, who meet the inclusion/exclusion criteria, regardless of religion, sex, or ethnic background, will be included in the study. Due to the nature of the study, and the fact that no benefit exists to the U.S. malaria-naïve volunteer, children will not be enrolled at this time. As per **Section 2.3.1.5**, Malaria infection during pregnancy can have adverse effects on both mother and fetus, including maternal anemia, fetal loss, premature delivery, intrauterine growth retardation and delivery of low birth-weight infants. As such, women who are pregnant or plan to become pregnant during the study period and persons <18 years of age are excluded from the study.

14.5 Subject Confidentiality

Subject confidentiality is held strictly in trust by the participating investigators, their staff, and the sponsor(s) and their agents. This confidentiality is extended to cover testing of biological samples in addition to the clinical information relating to participating subjects. Participants will be assigned a unique study number. All results will be keyed to this number. No information concerning the study or the data will be released to any unauthorized third party without prior written approval of the sponsor.

Study records will only be available to staff members and will be kept locked at the study site conforming to the investigators' SOPs. All computer entry will be done by coded number only, and all local databases will be secured with password-protected access systems. The study monitor or other authorized representatives of the sponsor may inspect all documents and records required to be maintained by the investigator, including but not limited to, medical records (office, clinic, or hospital) and pharmacy records for the subjects in this study. The clinical study site will permit access to such records.

14.6 Study Discontinuation

In the event that the study is discontinued prior to completion, all volunteers who received a malaria challenge and did not develop clinical symptoms of malaria, will be offered Coartem® or Malarone® malaria treatment doses to prevent erythrocytic-stage *P. falciparum* parasite development. Treatment doses are outlined in Section 8.1.2.4. The participants will be asked to follow up with the study team and complete Surveillance at day 28 (±3) post therapy to assure that malaria therapy is taken reliably and that malaria does not develop. In the event that the study volunteer has developed malaria with eradication of the parasite after protocol-specified treatment with Coartem® or Malarone®, the participant will be asked to follow up with the study team at the day 28 (±3) post therapy surveillance follow-up for debriefing. In the event that the study volunteer refuses drug treatment, attempts will be made to convince them to return to the CVD for daily malaria smears. If they refuse, daily contact with maintenance of solicited and unsolicited symptoms will be recorded. If a volunteer refuses therapy and symptoms of malaria develop, he/she will be asked to notify study personnel immediately and

treatment will be offered through the University of Maryland medical system. Study team personnel will be available for questions or follow-up should evaluation be needed prior to Surveillance day 28 (±3) post-therapy. Study team personnel will also be available for passive follow-up should symptoms arise relating to malaria.

14.7 Future Use of Stored Specimens

If residual plasma, and PBMC are available following the assays described in this protocol, additional immunological or microbiological assays may be performed on those samples. The residual specimens can be shared with other investigators listed on the protocol, and will be linked to the participant using their study identification number only if the participant grants permission for samples to be linked to their study information. If a participant requests that samples be de-identified before further studies are conducted, then these samples will be de-identified for future studies. Residual specimens may be maintained at the CVD in Baltimore, Maryland or at Uniformed Services University of the Health Sciences. Future studies utilizing these samples must first be approved by the University of Maryland and Uniformed Services University of the Health Sciences IRBs. No increased risk to the study participants is expected in association with the storage of residual specimens. Subjects must agree to the storage and use of their residual specimens and will state their preference on the informed consent form (refer to study informed consent document). Subjects will not be re-contacted for additional information or to receive the results of future studies.

15 DATA HANDLING AND RECORD KEEPING

15.1 Data Management Responsibilities

The principal investigator is responsible to ensure the accuracy, completeness, legibility, and timeliness of the data reported.

According to ICH Good Clinical Practice Guidance E6 (1.51, 1.52) source documents are "original documents, data and records…" These original records, including certified copies, contain information on "clinical findings, observations, or other activities in a clinical trial necessary for the reconstruction and evaluation of the trial. Source data are contained in source documents."

Forms for use as source documents will be developed by investigators and maintained on site at the University of Maryland, to record and maintain data for each participant enrolled in the study. All source documents should be completed in a neat, legible manner to ensure accurate interpretation of data. Black or blue ink is required to ensure clarity of reproduced copies. When making a change or correction, cross out the original entry with a single line and initial and date the change. Do not erase, overwrite, or use correction fluid or tape on the original. If a form is modified by the site, indicate in the footer the site name/code next to the version number and date (e.g., v1.0; 10JUL2011 UMD).

All source documents and laboratory reports must be reviewed by the clinical team and data entry staff, who will ensure that they are accurate and complete. Adverse events must be graded, assessed for seriousness, severity and causal relationship, and reviewed by the site principal investigator or designee. Data collection is the responsibility of the clinical trial staff at the site under the supervision of the site principal investigator. During the study, the investigator must maintain complete and accurate documentation for the study. The EMMES Corporation, The SDCC for this study, will be responsible for data management, quality review, analysis, and reporting of the study data.

It is the investigator's responsibility to follow written internal quality management activities to ensure accuracy and completion of study data pertaining to:

- Source documentation,
- CRFs, including how they are derived,
- Instructions for completing forms,
- Data handling, security, and monitoring,
- Maintaining subject confidentiality,
- Record retention per the sponsor's requirements

15.2 Data Capture Methods

Clinical data (including AEs, concomitant medications, and reactogenicity data) and clinical laboratory data will be entered into source documents. Clinical (including, but not limited to, AE/SAEs, concomitant medications, medical history, physical assessments, and clinical laboratory values), data will be entered into the 21 CFR 11-compliant Internet Data Entry System provided by the EMMES Corporation. The data system includes password protection and internal quality checks, such as automatic range checks, to identify data that appear inconsistent, incomplete, or inaccurate.

15.3 Types of Data

Data for this study will include safety, laboratory (clinical safety and immunologic), and efficacy outcome measures (e.g. reactogenicity, immunogenicity, and PCR quantification).

15.4 Timing/Reports

Study documents will be retained for a minimum of 2 years after the last approval of a marketing application and until there are no pending or contemplated marketing applications, or when at least 2 years have elapsed since the formal discontinuation of clinical development of an investigational product. These documents will be retained for a longer period, however, if required by local regulations. No record will be destroyed without the written consent of the sponsor.

The Clinical Study Report (CSR) will be completed when all primary and secondary endpoint data are available. Results for exploratory endpoint data analysis will be included in an addendum to the CSR .

15.5 Study Records Retention

Study records and reports, including, but not limited to, case report forms (CRFs), source documents, informed consent forms, laboratory test results, and medication inventory records, shall be retained for a minimum of 2 years, as required by the FDA, after the last CHMI follow up visit. The site must contact DMID for authorization prior to the destruction of any study records. No records will be destroyed without the written consent of Sanaria and DMID. It is the responsibility of Sanaria and DMID to inform the investigator when these documents no longer need to be retained.

15.6 Protocol Deviations

A protocol deviation is any noncompliance with the clinical trial protocol, Good Clinical Practice (GCP), or Manual of Procedures requirements. The noncompliance may be either on the part of the subject, the investigator, or the study site staff. As a result of deviations, corrective actions are to be developed by the site and implemented promptly.

It is the responsibility of the site to use continuous vigilance to identify and report deviations within 5 working days of identification of the protocol deviation or within 5 working days of the scheduled protocol-required activity. All deviations must be promptly reported to DMID.

These practices are consistent with ICH E6:

4.5 Compliance with Protocol, Sections 4.5.1, 4.5.2, and 4.5.3

5.1 Quality Assurance and Quality Control, Section 5.1.1

5.20 Noncompliance, Sections 5.20.1, and 5.20.2

All deviations from the protocol must be addressed in study subject source documents. A completed copy of the DMID Protocol Deviation Form (TRI/ICON DMID-CROMS or IDES form) must be maintained in the regulatory file, as well as in the subject's source document. Protocol deviations must be sent to the local IRB/IEC per their guidelines. The study site PI/study staff is responsible for knowing and adhering to their IRB/IEC requirements.

16 PUBLICATION POLICY

Following completion of the study, the investigator may publish the results of this research in a scientific journal. The International Committee of Medical Journal Editors (ICMJE) member journals have adopted a trials-registration policy as a condition for publication. This policy requires that all clinical trials be registered in a public trials registry such as ClinicalTrials.gov, which is sponsored by the National Library of Medicine (NLM). Other biomedical journals are considering adopting similar policies. It is the responsibility of DMID to register this trial in an acceptable registry. In compliance with Public Law 110-85, the Food and Drug Administration Amendments Act of 2007 (FDAAA), DMID will also post the results of the trial in accordance to the legal requirements.

The ICMJE defines a clinical trial as any research project that prospectively assigns human participants to intervention or comparison groups to study the cause-and-effect relationship between a medical intervention and a health outcome. Studies designed for other purposes, such as to study pharmacokinetics or major toxicity (*e.g.*, Phase 1 trials), would be exempt from this policy. As a result, this study can but is not required to be registered in the NLM registry, ClinicalTrials.gov.

All investigators funded by the NIH must submit or have submitted for them to the National Library of Medicine's PubMed Central an electronic version of their final, peer-reviewed manuscripts upon acceptance for publication, to be made publicly available no later than 12 months after the official date of publication. The NIH Public Access Policy ensures the public has access to the published results of NIH funded research. It requires investigators to submit final peer-reviewed journal manuscripts that arise from NIH funds to the digital archive PubMed Central upon acceptance for publication. Further, the policy stipulates that these papers must be accessible to the public on PubMed Central no later than 12 months after publication.

17 LITERATURE REFERENCES

Reference List

1. Stoute JA, Slaoui M, Heppner DG et al. A preliminary evaluation of a recombinant circumsporozoite protein vaccine against *Plasmodium falciparum* malaria. RTS,S Malaria Vaccine Evaluation Group. N Engl J Med 1997;336(2):86-91.

2. Moorthy VS, Ballou WR. Immunological mechanisms underlying protection mediated by RTS,S: a review of the available data. Malar J 2009;8:312.

3. Gordon DM, McGovern TW, Krzych U et al. Safety, immunogenicity, and efficacy of a recombinantly produced *Plasmodium falciparum* circumsporozoite protein-hepatitis B surface antigen subunit vaccine. J Infect Dis 1995;171(6):1576-1585.

4. Moorthy VS, Diggs C, Ferro S et al. Report of a consultation on the optimization of clinical challenge trials for evaluation of candidate blood stage malaria vaccines, 18-19 March 2009, Bethesda, MD, USA. Vaccine 2009;27(42):5719-5725.

5. Standardization of Design and Conduct of *P. falciparum* Sporozoite Challenge Trials. 10 A.D. Jun; 2011.

6. Epstein JE, Rao S, Williams F et al. Safety and clinical outcome of experimental challenge of human volunteers with *Plasmodium falciparum*-infected mosquitoes: an update. J Infect Dis 2007;196(1):145-154.

7. Lyke KE, Laurens M, Adams M et al. *Plasmodium falciparum* malaria challenge by the bite of aseptic *Anopheles stephensi* mosquitoes: results of a randomized infectivity trial. PLoS One 2010;5(10):e13490.

8. Collins WE, JEFFERY GM. A retrospective examination of secondary sporozoite- and trophozoite-induced infections with Plasmodium falciparum: development of parasitologic and clinical immunity following secondary infection. Am J Trop Med Hyg 1999;61(1 Suppl):20-35.

9. Coggeshall LT, Maier J, Best CA. The effectiveness of two new types of chemotherapeutic agents in malari. JAMA 1941;117:1077-1081.

10. Fairley NH. Sidelights on malaria in man obtained by subinoculation experiments. Trans R Soc Trop Med Hyg 1947;40:621-676.

11. Cooper WC, Coatney GR. Studies in human malaria iii. the therapeutic effect of a phenanthrene amino alcohol, nih-204 (sn-1796), in vivax, falciparum, and quartan malaria. Am J Hyg 1947;46:119-130.

12. MILLER MJ. Suppression of malaria by monthly drug administration. Am J Trop Med Hyg 1955;4(5):790-799.

13. DAVEY DG, ROBERTSON GI. Experiments with antimalarial drugs in man. IV. An experiment to investigate the prophylactic value of proguanil against a strain of *Plasmodium falciparum* known to be resistant to therapeutic treatment. Trans R Soc Trop Med Hyg 1957;51(5):463-466.

14. JEFFERY GM. Infectivity to mosquitoes of *Plasmodium vivax* following treatment with chloroquine and other antimalarials. Am J Trop Med Hyg 1958;7(2):207-211.

15. JEFFERY GM, YOUNG MD, BURGESS RW, EYLES DE. Early activity in sporozoite-induced *Plasmodium falciparum* infections. Ann Trop Med Parasitol 1959;53(1):51-58.

16. Laing AB. Antimalarial effects of sulphormethoxine, diaphenylsulphone and separate combinations of these with pyrimethamine: a review of preliminary investigations carried out in Tanzania. J Trop Med Hyg 1968;71(2):27-35.

17. Powell RD, McNamara JV. Infection with chloroquine-resistant Plasmodium falciparum in man: prepatent periods, incubation periods, and relationships between parasitemia and the onset of fever in nonimmune persons. Ann N Y Acad Sci 1970;174(2):1027-1041.

18. Collins WE, JEFFERY GM. A retrospective examination of sporozoite- and trophozoite-induced infections with Plasmodium falciparum: development of parasitologic and clinical immunity during primary infection. Am J Trop Med Hyg 1999;61(1 Suppl):4-19.

19. Chulay JD, Schneider I, Cosgriff TM et al. Malaria transmitted to humans by mosquitoes infected from cultured *Plasmodium falciparum*. Am J Trop Med Hyg 1986;35(1):66-68.

20. Herrington DA, Clyde DF, Losonsky G et al. Safety and immunogenicity in man of a synthetic peptide malaria vaccine against *Plasmodium falciparum* sporozoites. Nature 1987;328(6127):257-259.

21. Ballou WR, Hoffman SL, Sherwood JA et al. Safety and efficacy of a recombinant DNA *Plasmodium falciparum* sporozoite vaccine. Lancet 1987;329(8545):1277-1281.

22. Herrington DA, Clyde DF, Murphy JR et al. A model for *Plasmodium falciparum* sporozoite challenge and very early therapy of parasitaemia for efficacy studies of sporozoite vaccines. Trop Geogr Med 1988;40(2):124-127.

23. Clyde DF, McCarthy VC, Miller RM, Hornick RB. Specificity of protection of man immunized against sporozoite-induced falciparum malaria. Am J Med Sci 1973;266(6):398-403.

24. Nussenzweig RS, Vanderberg J, Most H, Orton C. Protective immunity produced by the injection of x-irradiated sporozoites of plasmodium berghei. Nature 1967;216(111):160-162.

25. Clyde DF. Immunization of man against falciparum and vivax malaria by use of attenuated sporozoites. Am J Trop Med Hyg 1975;24(3):397-401.

26. Rieckmann KH, Beaudoin RL, Cassells JS, Sell KW. Use of attenuated sporozoites in the immunization of human volunteers against falciparum malaria. Bull World Health Organ 1979;57 Suppl 1:261-265.

27. Herrington D, Davis J, Nardin E et al. Successful immunization of humans with irradiated malaria sporozoites: humoral and cellular responses of the protected individuals. Am J Trop Med Hyg 1991;45(5):539-547.

28. Hoffman SL, Isenbarger D, Long GW et al. Sporozoite vaccine induces genetically restricted T cell elimination of malaria from hepatocytes. Science 1989;244(4908):1078-1081.

29. Wang R, Doolan DL, Le TP et al. Induction of antigen-specific cytotoxic T lymphocytes in humans by a malaria DNA vaccine. Science 1998;282(5388):476-480.

30. Kester KE, McKinney DA, Tornieporth N et al. Efficacy of recombinant circumsporozoite protein vaccine regimens against experimental *Plasmodium falciparum* malaria. J Infect Dis 2001;183(4):640-647.

31. Church LW, Le TP, Bryan JP et al. Clinical manifestations of *Plasmodium falciparum* malaria experimentally induced by mosquito challenge. J Infect Dis 1997;175(4):915-920.

32. Hoffman SL, Goh LM, Luke TC et al. Protection of humans against malaria by immunization with radiation-attenuated *Plasmodium falciparum* sporozoites. J Infect Dis 2002;185(8):1155-1164.

33. McCarthy VC, Clyde DF. *Plasmodium vivax*: correlation of circumsporozoite precipitation (CSP) reaction with sporozoite-induced protective immunity in man. Exp Parasitol 1977;41(1):167-171.

34. Epstein JE, Tewari K, Lyke KE et al. Live Attenuated Malaria Vaccine Designed to Protect through Hepatic CD8+ T Cell Immunity. Science 2011;334(6055):425-80.

35. Seder RA, Chang LJ, Enama ME et al. Protection Against Malaria by Intravenous Immunization with a Nonreplicating Sporozoite Vaccine. Science 2013;341(6152):1359-1365.

36. McConkey SJ, Reece WH, Moorthy VS et al. Enhanced T-cell immunogenicity of plasmid DNA vaccines boosted by recombinant modified vaccinia virus Ankara in humans. Nat Med 2003;9(6):729-735.

37. Hermsen CC, de Vlas SJ, van Gemert GJ, Telgt DS, Verhage DF, Sauerwein RW. Testing vaccines in human experimental malaria: statistical analysis of parasitemia

measured by a quantitative real-time polymerase chain reaction. Am J Trop Med Hyg 2004;71(2):196-201.

38. Hoffman SL, Edelman R, Bryan JP et al. Safety, immunogenicity, and efficacy of a malaria sporozoite vaccine administered with monophosphoryl lipid A, cell wall skeleton of mycobacteria, and squalane as adjuvant. Am J Trop Med Hyg 1994;51(5):603-612.

39. Verhage DF, Telgt DS, Bousema JT et al. Clinical outcome of experimental human malaria induced by *Plasmodium falciparum*-infected mosquitoes. Neth J Med 2005;63(2):52-58.

40. Nieman AE, de MQ, Roestenberg M et al. Cardiac complication after experimental human malaria infection: a case report. Malar J 2009;8:277.

41. Taylor TE, Fu WJ, Carr RA et al. Differentiating the pathologies of cerebral malaria by postmortem parasite counts. Nat Med 2004;10(2):143-145.

42. Seydel KB, Milner DA, Jr., Kamiza SB, Molyneux ME, Taylor TE. The distribution and intensity of parasite sequestration in comatose Malawian children. J Infect Dis 2006;194(2):208-5.

43. Laurens MB, Duncan CJ, Epstein JE et al. A consultation on the optimization of controlled human malaria infection by mosquito bite for evaluation of candidate malaria vaccines. Vaccine 2012;30(36):5302-5304.

44. Edelman R, Hoffman SL, Davis JR et al. Long-term persistence of sterile immunity in a volunteer immunized with X-irradiated *Plasmodium falciparum* sporozoites. J Infect Dis 1993;168(4):1066-1070.

45. Langhorne J, Ndungu FM, Sponaas AM, Marsh K. Immunity to malaria: more questions than answers. Nat Immunol 2008;9(7):725-732.

46. Butler NS, Moebius J, Pewe LL et al. Therapeutic blockade of PD-L1 and LAG-3 rapidly clears established blood-stage Plasmodium infection. Nat Immunol 2012;13(2):188-195.

47. Marsh K, Kinyanjui S. Immune effector mechanisms in malaria. Parasite Immunol 2006;28(1-2):51-60.

48. Bouharoun-Tayoun H, Attanath P, Sabchareon A, Chongsuphajaisiddhi T, Druilhe P. Antibodies that protect humans against Plasmodium falciparum blood stages do not on their own inhibit parasite growth and invasion in vitro, but act in cooperation with monocytes. J Exp Med 1990;172(6):1633-1641.

49. Cohen S, McGregor IA, CARRINGTON S. Gamma-globulin and acquired immunity to human malaria. Nature 1961;192:733-737.

50. Schwenk RJ, Richie TL. Protective immunity to pre-erythrocytic stage malaria. Trends Parasitol 2011;27(7):306-314.

51. Weiss GE, Traore B, Kayentao K et al. The *Plasmodium falciparum*-specific human memory B cell compartment expands gradually with repeated malaria infections. PLoS Pathog 2010;6(5):e1000912.

52. Crompton PD, Pierce SK, Miller LH. Advances and challenges in malaria vaccine development. J Clin Invest 2010;120(12):4168-4178.

53. Crompton PD, Kayala MA, Traore B et al. A prospective analysis of the Ab response to Plasmodium falciparum before and after a malaria season by protein microarray. Proc Natl Acad Sci U S A 2010;107(15):6958-6963.

54. Walther M, Jeffries D, Finney OC et al. Distinct roles for FOXP3 and FOXP3 CD4 T cells in regulating cellular immunity to uncomplicated and severe Plasmodium falciparum malaria. PLoS Pathog 2009;5(4):e1000364.

55. Freitas do Rosario AP, Lamb T, Spence P et al. IL-27 promotes IL-10 production by effector Th1 CD4+ T cells: a critical mechanism for protection from severe immunopathology during malaria infection. J Immunol 2012;188(3):1178-1190.

56. Couper KN, Blount DG, Riley EM. IL-10: the master regulator of immunity to infection. J Immunol 2008;180(9):5771-5777.

57. Walther M, Woodruff J, Edele F et al. Innate immune responses to human malaria: heterogeneous cytokine responses to blood-stage Plasmodium falciparum correlate with parasitological and clinical outcomes. J Immunol 2006;177(8):5736-5745.

58. Ockenhouse CF, Hu WC, Kester KE et al. Common and divergent immune response signaling pathways discovered in peripheral blood mononuclear cell gene expression patterns in presymptomatic and clinically apparent malaria. Infect Immun 2006;74(10):5561-5573.

59. McGregor IA, Carrington SP, Cohen S. Treatment of East African *Plasmodium falciparum* with West African human gammaglobulin. Trans R Soc Trop Med Hyg 1963;57:170-175.

60. Reddy ST, Ge X, Miklos AE et al. Monoclonal antibodies isolated without screening by analyzing the variable-gene repertoire of plasma cells. Nat Biotechnol 2010;28(9):965-969.

61. Boyd SD, Marshall EL, Merker JD et al. Measurement and clinical monitoring of human lymphocyte clonality by massively parallel VDJ pyrosequencing. Sci Transl Med 2009;1(12):12ra23.

62. Doolan DL, Mu Y, Unal B et al. Profiling humoral immune responses to P. falciparum infection with protein microarrays. Proteomics 2008;8(22):4680-4694.

63. Rts, S. C. T. P. Efficacy and safety of the RTS,S/AS01 malaria vaccine during 18 months after vaccination: a phase 3 randomized, controlled trial in children and young infants at 11 African sites. PLoS Med 2014;11:e1001685.

64. Turner L, Wang CW, Lavstsen, SB. Antibodies agasint PfEMP1, RIFIN, MSP3, GLURP are acquired during controlled Plasmodium falciparum malaria infections in naïve volunteers. PLoS One 2011;6(12):e29025.

65. Severe falciparum malaria. World Health Organization, Communicable Diseases Cluster. Trans R Soc Trop Med Hyg 2000;94 Suppl 1:S1-90.

66. Gaziano TA, Young CR, Fitzmaurice G, Atwood S, Gaziano JM. Laboratory-based versus non-laboratory-based method for assessment of cardiovascular disease risk: the NHANES I Follow-up Study cohort. Lancet 2008;371(9616):923-931.

67. Davis JR. Laboratory methods for the conduct of experimental malaria challenge of volunteers. Vaccine 1994;12(4):321-327.

68. Beier JC, Davis JR, Vaughan JA, Noden BH, Beier MS. Quantitation of *Plasmodium falciparum* sporozoites transmitted in vitro by experimentally infected *Anopheles gambiae* and *Anopheles stephensi*. Am J Trop Med Hyg 1991;44(5):564-570.

69. Jin Y, Kebaier C, Vanderberg J. Direct microscopic quantification of dynamics of *Plasmodium berghei* sporozoite transmission from mosquitoes to mice. Infect Immun 2007;75(11):5532-5539.

70. Price R, Nosten F, Simpson JA et al. Risk factors for gametocyte carriage in uncomplicated falciparum malaria. Am J Trop Med Hyg 1999;60(6):1019-1023.

71. Hanscheid T. Diagnosis of malaria: a review of alternatives to conventional microscopy. Clin Lab Haematol 1999;21(4):235-245.

72. McCutchan TF, de lC, V, Lal AA, Gunderson JH, Elwood HJ, Sogin ML. Primary sequences of two small subunit ribosomal RNA genes from *Plasmodium falciparum*. Mol Biochem Parasitol 1988;28(1):63-68.

73. Gunderson JH, Sogin ML, Wollett G et al. Structurally distinct, stage-specific ribosomes occur in Plasmodium. Science 1987;238(4829):933-937.

74. Ruggeberg JU, Gold MS, Bayas JM et al. Anaphylaxis: case definition and guidelines for data collection, analysis, and presentation of immunization safety data. Vaccine 2007;25(31):5675-5684.

75. Lydersen S, Fagerland MW, Laake P. Recommended tests for association in 2 x 2 tables. Stat Med 2009;28(7):1159-1175.

76. Mehrotra DV, Chan IS, Berger RL. A cautionary note on exact unconditional inference for a difference between two independent binomial proportions. Biometrics 2003;59(2):441-450.

77. Maecker HT, McCoy JP, Nussenblatt R. Standardizing immunophenotyping for the Human Immunology Project. Nat Rev Immunol 2012;12(3):191-200.

78. Roederer M, Nozzi JL, Nason MC. SPICE: exploration and analysis of post-cytometric complex multivariate datasets. Cytometry A 2011;79(2):167-174.

79. Gallo V, Skorokhod OA, Schwarzer E, Arese P. Simultaneous determination of phagocytosis of Plasmodium falciparum-parasitized and non-parasitized red blood cells by flow cytometry. Malar J 2012;11:428.

80. Hill DL, Eriksson EM, Carmagnac AB et al. Efficient measurement of opsonising antibodies to Plasmodium falciparum merozoites. PLoS One 2012;7(12):e51692.

81. Crompton PD, Miura K, Traore B et al. In vitro growth-inhibitory activity and malaria risk in a cohort study in mali. Infect Immun 2010;78(2):737-745.

82. Tusher VG, Tibshirani R, Chu G. Significance analysis of microarrays applied to the ionizing radiation response. Proc Natl Acad Sci U S A 2001;98(9):5116-5121.

SUPPLEMENTS/APPENDICES

APPENDIX A: INFORMED CONSENT QUIZ

Repetitive *Plasmodium falciparum* (Pf) Malaria of the NF54 strain Controlled Human Challenge Infection (CHMI) Study in Malaria-Naïve Adults

I understand that the purpose of this written examination is to test my knowledge of certain aspects of this study. It will help to ensure that my consent to volunteer for the study is informed (in other words that I understand what the study is about). I realize that I must answer 7 of 10 questions correctly (70%) in order to be allowed to participate as a volunteer.

Signature of Volunteer: ________________________________ **Date:** ___________________

Witness: ____

Exam Score	Incorrect answers reviewed with volunteer?	
	☐ Yes ☐ No	Initial & Date

Please circle ONE answer only for each question:

1. If you participate in this study, you will receive an investigational product designed to test which of the following diseases?
   A. Malaria
   B. Chicken Pox
   C. Leprosy

2. Is it possible to get malaria from the mosquitoes used in this study?
   A. Yes
   B. No

3. Medical screening for this study will include which of the following?
   A. Laboratory tests (including an HIV test)
   B. Physical examination
   C. Review of medical history

D. All of the above

4. By participating in this study, you may develop malaria.

A. True

B. False

5. What are common findings associated with malaria infection?

A. Fever

B. Chills

C. Headache

D. All of the above

E. None of the above

6. If you participate in this study, for how long will you be unable to donate blood?

A. 3 years

B. 25 years

C. Never able to donate blood again

7. If you participate in this study, you may:

A. Withdraw voluntarily at any time.

B. Never withdraw from the study.

8. Which of the following are true regarding pregnancy and participation in this study?

A. Pregnant women may participate in this study.

B. Women should not get pregnant for 12 months after getting malaria.

C. An effective method of birth control is required for women, who are not surgically sterile, until day 28 post-therapy while participating in this study.

9. How long will this study last?

A. 1 month

B. 1 year

C. 3-4 years

D. 5 years

10. Which of the following is a component of this study?

A. Following up for up to 20 outpatient visits, including daily visits from Day 6-19 during the challenge phase

B. Staying overnight in a hospital for up to a month during the challenge phase

C. Coming and going as I please throughout the study

APPENDIX B: TOXICITY TABLE

**Local Reactogenicity Assessments**			
	**Grade**	**Intensity Definition**	
Pain	0	Absent	
	1	Does not interfere with activity	
	2	Repeated use of non-narcotic pain reliever >24 hours OR interferes with activity	
	3	Any use of narcotic pain reliever OR prevents daily activity	
Tenderness	0	Absent	
	1	Discomfort only to touch	
	2	Discomfort with movement	
	3	Significant discomfort at rest	
Erythema/redness	0	Absent	
	1	2.5 - 5 cm	
	2	5.1 - 10 cm	
	3	>10 cm	
Induration/Swelling	0	Absent	
	1	2.5 - 5 cm and does not interfere with activity	
	2	5.1 - 10 cm OR interferes with activity	
	3	>10 cm OR prevents daily activity	
Pruritus	0	Absent	
	1	Slight itching a mosquito bite site	
	2	Moderate itching at mosquito bite site	
	3	Itching over entire body	
**Systemic Symptoms Assessment**			
Malaise	0	Absent	
	1	Malaise that is easily tolerated	
	2	Malaise that interferes with daily activity	
	3	Malaise that prevents daily activity	
Chills/rigors	0	Absent	
	1	Symptoms that are easily tolerated	
	2	Symptoms that interfere with daily activity	
	3	Symptoms that prevent daily activity	
Nausea	0	Absent	
	1	Nausea that is easily tolerated	
	2	Nausea that interferes with daily activity	
	3	Nausea that prevents daily activity	
Vomiting	0	Absent	
	1	No interference with activity OR 1 - 2 episodes/24 hours	
	2	Some interference with activity OR >2 episodes/24 hours	
	3	Prevents daily activity OR requires IV hydration OR requires medical intervention	
Fever	0	< 38.0°C	< 100.4°F
	1	38.0 - 38.4°C	100.4 – 101.12°F
	2	>38.4 - 38.9°C	>101.12 – 102.0°F
	3	> 38.9°C	> 102.0°F
Myalgia	0	Absent	
	1	Muscular pain that is easily tolerated	
	2	Muscular pain that interferes with daily activity	
	3	Muscular pain that prevents daily activity	
Headache	0	Absent	

	1	No interference with activity
	2	Repeated use of non-narcotic pain reliever >24 hours OR some interference with activity
	3	Significant; any use of narcotic pain reliever OR prevents daily activity OR requires triptans
All Other Conditions	0	Absent
	1	No interference with activity
	2	Some interference with activity not requiring medical intervention
	3	Prevents daily activity and requires medical intervention

Laboratory Reference Ranges and Toxicity Grading*

Laboratory	Normal	Mild (Grade 1)	Moderate (Grade 2)	Severe (Grade 3)
Hemoglobin (♀) –gm/dL	12.0-16.0	11.0 – 11.9	9.0 – 10.9	≤ 8.9
Hemoglobin (♂) –gm/dL	13.5-17.5	12.5 – 13.4	9.5 – 12.4	≤ 9.4
WBC – x 10^3 cells/mm^3 (Leukocytosis)	3.5-10.0	>10.0 & < 15.0	≥ 15 & < 20	≥ 20
WBC – x 10^3 cells/mm^3 (Leukopenia)	3.5-10.0	<3.5 & ≥ 2.5	< 2.5 & ≥ 1.5	<1.5
Platelets – x 10^3 cell/mm^3	150-415	120-149	89-119	<89
ASTs (increase by factor) (♀) IU/L	0-30	>1.0 & <2.5 x ULN	≥ 2.5 & <5 x ULN	≥5.0 x ULN
ASTs (increase by factor) (♂) IU/L	0-40	>1.0 & <2.5 x ULN	≥ 2.5 & <5 x ULN	≥5.0 x ULN
ALTs (increase by factor) IU/L	0-45	>1.0 & <2.5 x ULN	≥ 2.5 & <5 x ULN	≥ 5x ULN*
Serum creatinine – mg/dL	0.6-1.4	1.5– 1.7	1.8 – 2.5	>2.5

ULN – Upper Limit of Normal, LLN – Lower Limit of Normal
*Modified to adhere to local laboratory reference ranges.

Vital Signs			
**Vital Signs**	**Mild (Grade 1)**	**Moderate (Grade 2)**	**Severe (Grade 3)**
**Fever °C (°F)***	38.0-38.4<br>(100.4 – 101.12)	>38.4-38.9<br>(>101.12 – 102.0)	> 38.9<br>(>102.0)
**Hypertension (systolic) mm Hg ** **	141-150	151-160	>160
**Hypertension (diastolic) mm Hg**	91-95	96-100	>100
**Hypotension (systolic) mm Hg**	85-89	80-84	<80
**Bradycardia – beats per minute*** **	50-54<br>or 45-50 if baseline <60	45-49<br>or 40-44 if baseline <60	<45<br>or <40 if baseline <60
**Tachycardia – beats per minute**	101-115	116-130	>130<br>or ventricular dysrhythmia
**Tachypnea – respirations per minute**	17-20	21-25	>25

* Oral temperature; no recent hot or cold beverages or smoking

** Assuming supine position, 10 minutes at rest conditions, not sleeping subjects, measurements on the same arm and several concordant results

*** When resting heart rate is between 60 – 100 beats per minute. Use clinical judgment when characterizing bradycardia among some healthy subject populations, for example, conditioned athletes.

APPENDIX C: SCHEDULE OF EVENTS

Table C1: Schedule of Events and Procedures for Study Volunteers with Repeat Pf CHMI – uninfected challenge and CHMI #1

			**Uninfected Mosquito Challenge**								**Infected Mosquito Challenge Cycle 1**							
Visit Number		00-00A	01	02	03	04	05	06	07	08	IC 01	IC 02	IC 03	IC 04-06	IC 07-17	IC 18	IC 19	IC 20
Study Day[a]		-56 to -1	1	2	4	6	8	10	13	21	1	2	4	6-8	9-19[b,c,d]	21[m]	29 [m]	37-56[m]
**Clinical Evaluations**	Tube										CHMI							
AofU & Study Consent		X																
PE, ht/wt/BMI/vitals at screen; vital signs and targeted exam[e] other visits		X	X							X	X			X	X	X	X	X
Eligibility Criteria Reviewed		X	X								X							
Medical History and Med Review		X	X	X	X	X	X	X	X	X	X	X	X	X	X	X	X	X
AE/SAE assessment[f,g]			X	X	X	X	X	X	X	X	X	X	X	X	**See Table C3**	X	X	
ECG – 12-lead		X																
Preg. test: urine or serum[g]		X	X								X						X	
CBC	EDTA	3								3	3					3		3
AST, ALT and creatinine	SST	4									4							4
HIV ELISA[h]/hepatitis serology [other tests, if needed]	SST	8									8							
Sickle cell test	EDTA	3																
Malaria diagnostic evaluation (smear and qPCR)[i]	EDTA													3		3[l]	3[l]	
**Research Samples**																		
PBMC and plasma storage	EDTA	30	50			30	30		30	30	80			30[j]		30		
Intracellular RNA	PAX		3	3	3	3	3	3	3	3	3	3	3	3[j]		3		
Leukapheresis										X						X[k]		
**Daily Volume (mL)**		**48**	**53**	**3**	**3**	**33**	**33**	**3**	**33**	**36**	**98**	**3**	**3**	**69**	**75-84**	**36**	**3**	**7**
**Max. Cumula tive Volume (mL)**		**48**	**101**	**104**	**107**	**140**	**173**	**176**	**209**	**245**	**98**	**93**	**96**	**165**	**240-249**	**276-285**	**279-288**	**283-295**

[a] The day of each challenge or CHMI is Study Day 1. Screening will occur over 1-2 visits.
[b] If malaria develops between Days 9-19, the patient will be treated as an inpatient x 3 days and discharged with outpatient
[d] Urine pregnancy test will be done on the day of the first positive malaria smear.
[e] Targeted exam including vital signs (heart rate, blood pressure, respiratory rate, temperature)
(qPCR will commence on D6 and smear on D9).
[j] To occur on Days 6 and 8.
[k] To occur 7 days (+3 days) after inpatient PBMC collection or on Day 21 (+3 days) if aparasitemic on day 13.

follow-up having met a primary endpoint. In the absence of a positive malaria smear and symptoms by surveillance day 19, participants will be discharged for follow-up as an outpatient.

[c] Complete blood count (CBC), creatinine, AST, ALT will be drawn on day of positive malaria smear and +28 days post-therapy or if aparasitemic D29 (±2).

[f] Includes Memory Aid (D1-5) and cardiovascular signs and symptoms

[g] Serum pregnancy test for first screen, urine pregnancy tests for all subsequent tests.

[h] HIV will be tested annually

[i] Study samples for malaria diagnosis will occur daily on D6-19

[l] Smear and PCR are to be done in those individuals who do not develop malaria.

[m] Outpatient f/u will correspond to apheresis day for repeat CHMI or 21±1 for infectivity controls, 29 (±2) and + 28 days post-therapy (±3) for all volunteers. Day 28 post-therapy represents D37-56.

Table C2: Schedule of Events and Procedures for Study Volunteers with Repeat Pf CHMI – CHMI #2-5

			**Infected Mosquito Challenge Cycles 2-4**							
Visit Number		00	IC 01	IC 02	IC 03	IC 04-06	IC 07-17	IC 18	IC 19	IC 20
Study Day[a]		-56 to -1	1	2	4	6-8	9-19[b,c,d]	21[l]	29[l]	37-56[l]
**Clinical Evaluations**	Tube		CHMI							
Confirm Study Consent		X								
Vital signs and targeted exam[e]		X	X			X	X	X	X	X
Eligibility Criteria Reviewed		X	X							
Medical History and Med Review		X	X	X	X	X	X	X	X	X
AE/SAE assessment[f]			X	X	X	X		X	X	
Pregnancy test: urine		X	X						X	
CBC	EDTA	3	3					3		3[l]
AST, ALT and creatinine	SST	4	4							4[l]
HIV ELISA[g] [other tests, if needed]	SST	8					**See Table C3**			
Malaria diagnostic evaluation (smear and qPCR)[h]	EDTA					3		3[k]	3[k]	
**Research Samples**										
PBMC and plasma storage	EDTA	30	50			30[i]		30		
Intracellular RNA	PAX		3	3	3	3		3		
Leukapheresis								X[j]		
**Daily Volume (mL)**		**45**	**60**	**3**	**3**	**69**	**75-84**	**36**	**3**	**7**
**Max. Cumulative Volume (mL)**		**45**	**105**	**108**	**111**	**180**	**255-264**	**291-300**	**294-303**	**301-310**

[a] The day of CHMI is Study Day 1.

[b] If malaria develops between Days 9-19, the patient will be treated x 3 days and discharged from daily follow-up having met a primary endpoint. In the absence of a positive malaria smear and symptoms by surveillance day 19, participants will be continue follow-up as an outpatient.

[c] Complete blood count (CBC), creatinine, AST, and ALT will be drawn on day of positive malaria smear (or PCR for controls of CHMI #5) and +28 days post-therapy.

[d] Urine pregnancy test will be done on the day of the first positive malaria smear. (or PCR for controls of CHMI 5)

[e] Targeted exam including vital signs (heart rate, blood pressure, respiratory rate, temperature)

[f] Includes Memory Aid (D1-5) and cardiovascular signs and symptoms

[g] HIV will be tested annually

[h] Study samples for malaria diagnosis will occur daily on D6-19 (qPCR will commence on D6 and smear on D9).

[i] To occur on days 6 and 8.

[j] To occur 7 days (+3) after the malaria-associated PBMC collection..

[k] Smear and PCR are to be done in those individuals who do not develop malaria.

[l] Outpatient f/u will correspond to Days 21 (+3), 29 (±2) and Days 28 post-therapy (±3). Day 28 post-therapy represents D37-56. Since 29 (±2) is the last visit for aparasitemic individuals they will have a CBC and biochemistry at that time.

Table C3: Schedule of Inpatient Stay and Malaria Event

Event	Inpatient (CHMI #2-4) or Daily Outpatient (CHMI #5) Surveillance[a]											
**Study Visit**	**07**	**08**	**09**	**10**	**11**	**12**	**13**	**14**	**15**	**16**	**17**	**Date of first positive malaria smear**	**Outpatient F/u[l]**
**Study Day[b]**	**9**	**10**	**11**	**12**	**13**	**14**	**15**	**16**	**17**	**18**	**19[c]**		
Daily Follow-up	X												
History/Med Review	X	X	X	X	X	X	X	X	X	X	X	X	X
Assesment of Malaria signs and symptoms, adverse events including cardiovascular	X	X	X	X	X	X	X	X	X	X	X	X	X
Physical Exam[d]	X	X	X	X	X	X	X	X	X	X	X	X	X
Hematology[e]												X	X
Safety[f]												X	X
Pregnancy Test[g]												X	X
Malaria Smear[h]	X	X	X	X	X	X	X	X	X	X	X	X	X
qPCR Analysis[i]	X	X	X	X	X	X	X	X	X	X	X	X	X
RNA Samples[j]		X		X		X		X		X			
PBMC/Plasma Sample[k]					X[k]								
Total Blood (mL)	3	6	3	6	33	6	3	6	3	6	3	7[m]	Refer to Table C1-2
Cumulative Blood (mL)	3	6	9	15	48	54	57	63	66	72	75	75-82[m]	

[a] If malaria develops between Days 9-19, the patient will be treated with Coartem or Malarone x 3 days and continue with follow-up having met a primary endpoint.

[b] Study Day 1 is the day of CHMI

[c] In the absence of a positive malaria diagnostic and symptoms by surveillance day 19, participants will discontinue daily follow-up.

[d] Targeted exam including vital signs (heart rate, blood pressure, respiratory rate, temperature)

[e] Complete blood count (CBC) will also be drawn on D1 of positive malaria smear and D28 (±3) post-therapy.

[f] Creatinine, AST and ALT will be drawn D1 of positive diagnostic (malaria smear or us-qPCR for infectivity controls of CHMI #5). They will also be drawn D28 (±3) post-therapy.

[g] Urine pregnancy test will be done on the day of the first positive malaria smear

[h] Malaria smear refers to thick blood smears to be drawn once daily (D9 until treatment or D19 if aparasitemic), and to continue on Days 21 (-1/+3) and 29 (±2). Symptomatic volunteers will have q 6-12 hour smears/us-qPCR to assess for malaria.

i Blood samples for research parasite DNA qPCR will commence on D6 and end after the second negative malaria smear or following anti-malarial therapy initiation (controls CHMI #5).

[j] RNA samples refers to research blood samples gene expression analysis (3ml) will be drawn every other day until leukapheresis.

[k] PBMC/plasma sample refers to research blood samples drawn (30 mL) collected just prior to treatment (±1 day). If the subject has not had a positive PCR by day 13 then the PBMC collection will be on day 13+1.[l] Outpatient f/u will correspond to apheresis day for repeat CHMI or Day 21 (±1) for infectivity controls, as well as Day 29 (±2) and Day 28 post-therapy (±3). Smear and PCR are to be done in those individuals who do not develop malaria. In addition, a CBC,biochemistries will be performed on apheresis day and Day 28 post therapy..

[m] Blood samples will be taken for a CBC, creatinine, AST, and ALT only if a subject has a positive malaria smear (or us-qPCR for controls in CHMI #5).

Table C4: Schedule of Events and Procedures for Naïve, Infectivity Controls

			Infected Mosquito Challenge Cycle							
Visit Number		00	IC 01	IC 02	IC 03	IC 04-06	IC 07-17	IC 18	IC 19	IC 20
Day of Study[a]		-56 to -1	1	2	4	6-8	9-19[b,c,d]	21[j]	29[j]	37-56[j]
**Clinical Evaluations**	Tube									
Assess. of Understanding and Study Consent		X								
PE, ht/wt/BMI/vitals at screen; vital signs and targeted exam[e] other visits		X	X			X	X	X	X	
Eligibility Criteria Reviewed		X	X							
Medical History and Med Review		X	X	X	X	X	X	X	X	X
AE/SAE assessment[f]			X	X	X	X	X	X	X	
EKG – 12-lead		X					**See Table C3**			
Preg. test:[g]		X	X						X	
CBC	EDTA	3	3							3
AST, ALT and creatinine	SST	4	4							4
HIV ELISA and hepatitis serologies [other tests, if needed]	SST	8								
Sickle cell test	EDTA	3								
Malaria diagnostic evaluation (smear and qPCR)[h]	EDTA					3	3	3[i]	3[i]	
**Research Samples**										
PBMC and plasma storage	EDTA									
Intracellular RNA	PAX									
Leukapheresis										
**Daily Volume (mL)**		**18**	**7**			**9**	**33**	**3**	**3**	**7**
**Max. Cumulative Volume (mL)**		**18**	**25**	**25**	**25**	**34**	**67**	**70**	**73**	**80**

[a] The day of CHMI is Study Day 1.

[b] If malaria develops between Days 9-19, the patient will be treated x 3 days and continue with follow-up having met a primary endpoint. In the absence of a positive malaria diagnostic and symptoms by surveillance day 19, participants will discontinue daily follow-up.

[c] Complete blood count (CBC), creatinine, AST, and ALT will be drawn on day of positive malaria smear (or 2nd us-qPCR for controls) and +28 days post-therapy

[d] Urine pregnancy test will be done on the day of the first positive malaria smear.

[e] Targeted exam including vital signs (heart rate, blood pressure, respiratory rate, temperature)

[f] Includes Memory Aid (D1-5) and cardiovascular signs and symptoms

[g] Serum pregnancy test for first screen, urine pregnancy tests for all subsequent tests.

[h] Study samples for malaria diagnosis will occur daily (D6-19 (qPCR will commence on D6 and smear on D9), Day, 21 () and 29 (±2). Smears will not be performed for CHMI #5.

[i] Smear and PCR are to be done in those individuals who do not develop malaria.

[j] Outpatient f/u will correspond to study Days 21 (±1), 29 (±2) and 28 days post-therapy (±3). If the volunteer remains aparasitemic, a CBC and Biochemistry will be performed on Study Day 29 (±2). +28 days post-therapy represents D37-56.

Table C5: Estimated time line for repeat challenge participants

1st Study Year						
	month	Day pCHMI	Study day			
	-2		-56	Recruitment		IRB/IND
	-1					
	0	1	1	**uninfectious feed**		
		13	13	outpatient every other day until day 13		
		21	21	day 21 apheresis-		
	1					
	2	1	61	**1st infectious feed**		
		8	68	outpatient every other day until day 8		
		~15	75	inpatient until 3 days post treatment		
		21	81	7 days post inpatient PBMC collection--apheresis		
	3	28	91	Day 29 check up		
	4	56	121	1 month follow up visit		
	5		151			
	6		181			
	7	Day pCHMI	211			
	8	1	241	**2nd infectious feed**		
		8	248	outpatient every other day until day 8		
		15	256	inpatient until 3 days post treatment		
		21	263	7 days post inpatient PBMC collection --apheresis		
	9	28	271	completed		
	10	56	301	1 month follow up visit		
	11	56	331			
2nd study year						
	Month	Day pCHMI				
	14-22	1	636	**3rd infectious feed**		
		8	643	outpatient every other day until day 8		
		15	650	inpatient until 3 days post treatment		
		21	656	7 days post in patient PBMC collection--apheresis		
	15-23	28	666	completed		
	16-24	56	696	1 month follow up visit		
3rd study year						
	Month	Day pCHMI				
	20-32	1	1001	**4th infectious feed**		
		8	1008	outpatient every other day until day 8		
		15	1015	inpatient until 3 days post treatment		

		21	1021	7 days post in patient PBMC collection--apheresis
	21-33	28	1031	Completed
	22-34	56	1061	1 month follow up visit
4th study year				
	Month	Day pCHMI		
	34-46	1	1436	**5th infectious feed**
		8	1444	outpatient every other day until day 8
		15	1451	Outpatient daily follow-up until 3 days post treatment
		21	1457	7 days post malaria-associated PBMC collection--apheresis
	35-47	28	1464	Completed
	36-48	56	1492	1 month follow up visit

APPENDIX D. BRIGHTON CASE DEFINITION OF ANAPHYLAXIS[74]

Anaphylaxis shall be defined when there is no clear alternative diagnosis to account for a combination of symptoms that are consistent with the case definition.

Diagnostic certainty

All levels	Sudden onset AND<br>Rapid progression of signs and symptoms AND<br>Involving multiple (>2) organ systems
Level 1	≥1 major dermatological AND<br>≥1 major cardiovascular AND/OR ≥1 major respiratory criterion
Level 2	≥1 major cardiovascular AND ≥1 major respiratory criterion<br>- OR -<br>≥1 major cardiovascular OR ≥1 major respiratory criterion AND<br>≥1 minor criterion involving ≥1 different system<br>- OR -<br>>1 major dermatologic AND ≥1 minor cardiovascular AND/OR ≥1 minor respiratory criterion
Level 3	≥1 minor cardiovascular OR respiratory criterion AND<br>≥1 minor criterion from each of ≥2 different systems/categories

Major criteria

Dermatologic or mucosal	• Generalized urticarial (hives) or generalized erythema<br>• Angioedema (not hereditary), localized or generalized<br>• Generalized pruritus with skin rash
Cardio-vascular	• Measured hypotension<br>• Clinical diagnosis of uncompensated shock, indicated by >3 of:<br>tachycardia, cap refill >3s, reduced central pulse volume, decreased level or loss of consciousness
Respiratory	• Bilateral wheeze (bronchospasm)<br>• Stridor<br>• Upper airway swelling (lip, tongue, throat, uvula, or larynx)<br>• Respiratory distress, indicated by >2 of:<br>Tachypnea, increased use of accessory muscles, recession, cyanosis, grunting

Minor criteria

Dermatologic or mucosal	• Generalized pruritus without skin rash<br>• Generalized prickle sensation<br>• Localized injection site urticaria<br>• Red and itchy eyes
Cardio-vascular	• Reduced peripheral circulation, indicated by ≥2 of:<br>Tachycardia, cap refill time >3s without hypotension, decreased level of consciousness
Respiratory	• Persistent dry cough<br>• Hoarse voice<br>• Difficulty breathing without wheeze or stridor<br>• Sensation of throat closure<br>• Sneezing, rhinorrhea
GI	• Diarrhea<br>• Abdominal pain<br>• Nausea<br>• Vomiting
Laboratory	• Mast cell tryptase elevation > upper normal limit
