# Supplementary material for: Repeat controlled human Plasmodium falciparum infections delay bloodstream patency and reduce symptoms
Source: Nat Commun. 2024 Jun 18;15:5194. doi: 10.1038/s41467-024-49041-2 (PMC11189388; doi:10.1038/s41467-024-49041-2)
Supplement: Supplementary file 3 — Reporting Summary [file 41467_2024_49041_MOESM3_ESM.pdf]

Corresponding author(s): Kim C Williamson and Kirsten E. Lyke

Last updated by author(s): May 1, 2024

Reporting Summary

Nature Portfolio wishes to improve the reproducibility of the work that we publish. This form provides structure for consistency and transparency in reporting. For further information on Nature Portfolio policies, see our Editorial Policies and the Editorial Policy Checklist.

Statistics

For all statistical analyses, confirm that the following items are present in the figure legend, table legend, main text, or Methods section.

n/a	Confirmed	
☐	☒	The exact sample size (*n*) for each experimental group/condition, given as a discrete number and unit of measurement
☐	☒	A statement on whether measurements were taken from distinct samples or whether the same sample was measured repeatedly
☐	☒	The statistical test(s) used AND whether they are one- or two-sided<br>*Only common tests should be described solely by name; describe more complex techniques in the Methods section.*
☐	☒	A description of all covariates tested
☐	☒	A description of any assumptions or corrections, such as tests of normality and adjustment for multiple comparisons
☐	☒	A full description of the statistical parameters including central tendency (e.g. means) or other basic estimates (e.g. regression coefficient) AND variation (e.g. standard deviation) or associated estimates of uncertainty (e.g. confidence intervals)
☐	☒	For null hypothesis testing, the test statistic (e.g. *F*, *t*, *r*) with confidence intervals, effect sizes, degrees of freedom and *P* value noted<br>*Give P values as exact values whenever suitable.*
☒	☐	For Bayesian analysis, information on the choice of priors and Markov chain Monte Carlo settings
☒	☐	For hierarchical and complex designs, identification of the appropriate level for tests and full reporting of outcomes
☐	☒	Estimates of effect sizes (e.g. Cohen's *d*, Pearson's *r*), indicating how they were calculated

Our web collection on statistics for biologists contains articles on many of the points above.

Software and code

Policy information about availability of computer code

Data collection	Bio-Rad Bio-Plex<br>BD Biosciences LSRII cytometer<br>Biotek Citation 5 plate reader
Data analysis	GraphPad Prism version 9·5·1<br>FlowJo software version 10.8.2

For manuscripts utilizing custom algorithms or software that are central to the research but not yet described in published literature, software must be made available to editors and reviewers. We strongly encourage code deposition in a community repository (e.g. GitHub). See the Nature Portfolio guidelines for submitting code & software for further information.

Data

Policy information about availability of data

All manuscripts must include a data availability statement. This statement should provide the following information, where applicable:

- Accession codes, unique identifiers, or web links for publicly available datasets
- A description of any restrictions on data availability
- For clinical datasets or third party data, please ensure that the statement adheres to our policy

De-identified data reporting the study's primary and secondary protocol-specified objectives are shown in Figures 3 and 4 and the source data for all the figures are provided as a Source Data file. The study protocol and inclusion exclusion criteria are included in the Supplementary Information, pgs 15-126 and available on ClinicalTrials.gov # NCT03014258. For investigators whose proposed use of the data have been approved by the UMB IRB, additional de-identified data will be made available within a month following a request to the corresponding authors.

Research involving human participants, their data, or biological material

Policy information about studies with human participants or human data. See also policy information about sex, gender (identity/presentation), and sexual orientation and race, ethnicity and racism.

Reporting on sex and gender	27 individuals were enrolled, 3 self-reported females and 24 self-reported males. Both male and female participants were recruited, but only males completed the repeat CHMIs. Therefore, the data reported for the repeat CHMIs was from male participants. The infectivity controls that only participated in one mosquito challenge included both females (n=3 and males (n=16).
Reporting on race, ethnicity, or other socially relevant groupings	Participants self-reported to be black (n=12), white (n-10), Asian (n=2) or multiracial (n=3)
Population characteristics	Healthy, malaria-naïve, U.S. participants ages 18-50 years, inclusive, from the greater Baltimore-Washington area
Recruitment	Participants were recruited from the greater Baltimore/Washington metropolitan area and from a panel of participants from previous non-malaria studies. Participants were recruited during the two months prior to the first CHMI, and subsequent infectivity control participants were recruited during the two months prior to each subsequent CHMI. All participants gave informed consent and were compensated for their time and inconvenience in the form of checks, as described below. Transportation reimbursement was also be provided. Participants were compensated up to $ 1,125-2,000 for each single malaria exposure.
Ethics oversight	The clinical trial protocol was reviewed and approved by the UMB Institutional Review Board (IRB) through a reliance agreement established with the USU IRB.

Note that full information on the approval of the study protocol must also be provided in the manuscript.

Field-specific reporting

Please select the one below that is the best fit for your research. If you are not sure, read the appropriate sections before making your selection.

[x] Life sciences [ ] Behavioural & social sciences [ ] Ecological, evolutionary & environmental sciences

For a reference copy of the document with all sections, see nature.com/documents/nr-reporting-summary-flat.pdf

Life sciences study design

All studies must disclose on these points even when the disclosure is negative.

Sample size	The sample size was selected to retain 80% power assuming 7 of the 10 experimental subjects complete all 4 P. falciparum challenges and a 0.7 probability of protection in the experimental subjects and zero probability of protection in the control subjects. Under these conditions there is a 0.87 probability of correctly rejecting the null hypothesis that the protective efficacy for experimental and control subjects are equal under 2-sided Type I error of 0.05 11.2 using Boschloo's test
Data exclusions	One participant had a very low CD4 T cell count and was not included in the calculating the CD4 T cell percentages. The two participants that did not participate in the mock were excluded from the flow cytometry analysis. The number of participants included in each analysis are indicated in the corresponding figure legend.
Replication	The multi-plex cytokine assays were done in duplicate and repeated 3 times. The ELISAs were done in duplicate and the baseline samples were repeated twice. All attempts to repeat experiments were successful. Due to the limited number of PBMCs available the immunophenotyping was not repeated, the B cell and T cell flow panels were performed on samples from each participant at each time point.
Randomization	Ten participants (with 2-4 alternates) were be recruited sequentially for participation into the core group for repeated exposure to Pf malaria by the bite of mosquito. They were not randomized.

Blinding	All participants had sequential exposures to P. falciparum infected mosquitoes. The samples were de-identified and coded prior to the humoral and cellular assays.

Reporting for specific materials, systems and methods

We require information from authors about some types of materials, experimental systems and methods used in many studies. Here, indicate whether each material, system or method listed is relevant to your study. If you are not sure if a list item applies to your research, read the appropriate section before selecting a response.

Materials & experimental systems

n/a	Involved in the study	
☐	☒	Antibodies
☒	☐	Eukaryotic cell lines
☒	☐	Palaeontology and archaeology
☒	☐	Animals and other organisms
☐	☒	Clinical data
☒	☐	Dual use research of concern
☒	☐	Plants

Methods

n/a	Involved in the study	
☒	☐	ChIP-seq
☐	☒	Flow cytometry
☒	☐	MRI-based neuroimaging

Antibodies

Antibodies used	For the B cell panel, 20 µl CD21-PE, 5µl CD24-PE-CF594, 5µl CD19-APC, 5µl IgG-APC-Cy7, 5µl CD27-BV421, 5µl CD10-BV605, 5µl CD38-BV711 and 5µl CD20-BUV395 (BD Biosciences) were used. For the T cell panel, 20 µl CD3-FITC (BD Biosciences), 5 µl CXCR6-PE (Biolegend), 5 µl CD69-PE-CF594 (BD Biosciences), 20 ul PD-1-APC (BD Biosciences), 5 µl CD8-APC-Cy7 (BD Biosciences), 5 µl CD45RO-BV421 (BD Biosciences), 5 µl CD4-BV510 (BD Biosciences), 5 µl CXCR5-BV605 (Biolegend), 5 µl CCR7-BV650 (Biolegend) and 5 µl CXCR3-BV711 (BD Biosciences) were used.
Validation	For the CSP ELISA an anti-CSP mAb from BEI as well as plasma from malaria exposed and naive donors for positive and negative controls were used.<br>The flow cytometry antibodies were first tested against PBMC obtained from a healthy volunteer.<br>The Multiplex assay used the positive and negative standards provided with the kit.

Clinical data

Policy information about clinical studies

All manuscripts should comply with the ICMJEguidelines for publication of clinical research and a completedCONSORT checklist must be included with all submissions.

Clinical trial registration

NCT03014258

Study protocol

ClinicalTrials.gov and a copy is included in the supplemental information.

Data collection

This single site clinical trial was based at the Center for Vaccine Development and Global Health within the University of Maryland School of Medicine, where recruitment and enrollment, malaria challenge events, and outpatient follow-up occurred. A mock and four infective CHMI's occurred from July 2017 to April 2020 whereupon the study was halted due to the SARS-CoV-2 pandemic, truncating CHMI #5. Overnight/hotel stays for each CHMI took place at an external hotel in Baltimore. Participants were recruited from the greater Baltimore/Washington metropolitan area and from a panel of participants from previous non-malaria studies studies. Participants were recruited during the two months prior to the first CHMI, and subsequent infectivity control participants were recruited during the two months prior to each subsequent CHMI. Males and females were recruited, but fewer females enrolled. Females participated in either the mock uninfected mosquito challenge or a single CHMI, but only male participants (n=8) completed the ~2 year course of repeat CHMIs.

Outcomes

Primary Outcome

Determine whether protective immunity against parasite infection develops following repeat CHMI

Measurements

1a. Occurrence of a positive malaria smear during the surveillance period after infected CHMI (+ 8-18, 20, and 28 days after challenge or until malaria detection (corresponding to Study Days 9-19, 21, and 29). For infectivity controls enrolled into infective CHMI #5, malaria detection would have been assessed upon us-qPCR to ensure safety while conducting this portion of the study as an outpatient.

1b. Occurrence and quantification of parasites using real-time quantitative PCR during the surveillance period after infected CHMI (+ 5-18, 20, 28 days after challenge or until malaria detection (Study Days 6-19, 21, 29).

Secondary Outcome

1. Determine whether clinical signs and symptoms of malaria decrease in intensity and duration following repeat CHMI.

2. Track the production of antibodies that react with P. falciparum sporozoites and blood stage parasites on Study days 6 and 8, treatment day and 7 days post treatment initiation or, if aparasitemic, Study Days 13 and 21 to compare responses over time and to sequential CHMI.

Measurements

1a. Occurrence of fever (>38oC) attributable to malaria during the surveillance period (Study Days 6-29 (±2 days) post-CHMI).

1b. Occurrence of signs and symptoms attributable to malaria (malaise, chills/rigors, nausea, vomiting, dizziness, arthralgia, abdominal pain, myalgia, and headache; see section 8.1.2.4) during the surveillance period (Study Days 6-29).

2. Antibody reactivity against Pf pre-erythrocytic antigen, CSP, and Pf liver- and erythrocytic-stage antigen, GLURP, measured using plasma obtained during the surveillance period Study Day 6, Study Day 8, treatment day or Study Day 13 if aparasitemic, and 7 days after treatment initiation or Study Day 21 if aparasitemic.

Plants

Seed stocks

not applicable

Novel plant genotypes

Describe the methods by which all novel plant genotypes were produced. This includes those generated by transgenic approaches, gene editing, chemical/radiation-based mutagenesis and hybridization. For transgenic lines, describe the transformation method, the number of independent lines analyzed and the generation upon which experiments were performed. For gene-edited lines, describe the editor used, the endogenous sequence targeted for editing, the targeting guide RNA sequence (if applicable) and how the editor was applied.

Authentication

Describe any authentication procedures for each seed stock used or novel genotype generated. Describe any experiments used to assess the effect of a mutation and, where applicable, how potential secondary effects (e.g. second site T-DNA insertions, mosiacism, off-target gene editing) were examined.

Flow Cytometry

Plots

Confirm that:

- [x] The axis labels state the marker and fluorochrome used (e.g. CD4-FITC).
- [x] The axis scales are clearly visible. Include numbers along axes only for bottom left plot of group (a 'group' is an analysis of identical markers).
- [x] All plots are contour plots with outliers or pseudocolor plots.
- [x] A numerical value for number of cells or percentage (with statistics) is provided.

Methodology

Sample preparation	Multi-parameter flow cytometry was used to assess unstimulated T and B cells from peripheral blood mononuclear cells (PBMC) isolated 7 days after treatment (DRx + 7) for each CHMI, as described above. For use in this assay, cryovials of PBMC samples were thawed for 60 s at 37°C. One milliliter of warm 1:19 diluted BSA stain buffer was added dropwise at a rate of 1 ml/5 s and mixed by inversion twice. Samples were transferred to 5 ml of warm BSA stain buffer and centrifuged at 3000 g for 5 m (brake 2) before washing with 3 ml of BSA stain buffer and centrifugation at 300 x g for 5 m (brake 2). Cells were then resuspended in 1 ml of BSA stain buffer, counted , and 1 x 106 cells were placed in each flow tube.<br>For the Live/Dead stain tube, cells were washed once with 1 ml of 1X PBS (pH 7.2, KD Medical), centrifuged at 3000 g for 5 m (brake 2) and the cell pellet resuspended in 1 ml 1X PBS and 1 ul reconstituted fluorescent reactive dye. After incubation on ice for 30 m protected from light, the cells were washed with BSA stain buffer (3 ml) and centrifuged at 3000 x g for 5 m (brake 2) and resuspended in BSA stain buffer (100 µl). For the B cell panel, non-specific Fc-mediated interactions were inhibited by adding 2.5 µg of Fc block (BD Biosciences) per tube of 1 x 106 PBMC and incubating at RT for 10 m. The fluorescent antibodies for the B-cell and T-cell panels listed above were added and mixed well. All flow tubes were incubated at RT for 25 m protected from light, then washed with BSA stain buffer (3 ml) and centrifuged at 3000 x g for 5 m (brake 2). The cell pellet was incubated in 500 ul of Biolegend Fluorofix Buffer at RT for 30 m, then washed with BSA stain buffer (3 ml) and resuspended in 500 ul of BSA stain buffer.
Instrument	LSRII cytometer (BD Biosciences)
Software	FlowJo software version 10.8.2 and GraphPad Prism version 9·5·1
Cell population abundance	The PBMC population included 12.5% B cells (CD19+) and 62.6% CD3+ T cells made up of 27% CD8+ and 47% CD4
Gating strategy	FSC-H vs FSC-A was used to select single cells and FSC-A and SSC-A was used to select lymphocytes which were tested for viability. Viable cell populations expressing CD19+CD3- were selected to assess B cell populations and CD3+CD20- cells were selected to assess T cell populations. The gating strategies are shown in Supplementary figure 10.

- [x] Tick this box to confirm that a figure exemplifying the gating strategy is provided in the Supplementary Information.
